# Unraveling the Wettability of Gold Surfaces: A Reactive Molecular Dynamics and Experimental Approach

**DOI:** 10.1021/acs.jpcc.6c04449

**Published:** 2026-09-28

**Authors:** Emdadul Haque Chowdhury, Md Shahed Hossain Sohan, Evan Buckwalter, Seong H. Kim, Adri C.T. van Duin, Bladimir Ramos-Alvarado

**Affiliations:** † Department of Mechanical Engineering, 8082 The Pennsylvania State University, University Park, Pennsylvania 16802, United States; ‡ Department of Chemical Engineering, The Pennsylvania State University, University Park, Pennsylvania 16802, United States

## Abstract

While early experimental work established that Au is intrinsically hydrophilic, meaning that the bare metal-water interface is strongly wetting in the absence of adsorbed contaminants, a knowledge gap between computational predictions and experimental measurements persists. Classical nonreactive force fields lack the accuracy to model this interface correctly, predicting contact angles that range from complete wetting to artificial hydrophobicity depending entirely on arbitrary interaction parameters or incompatible optimization objectives. To bridge this computational-experimental gap, we developed a ReaxFF reactive force field for the Au-water interface, combining established bulk parameter sets with solid–liquid interactions trained strictly against van der Waals-corrected DFT data. A benchmark of nine nonreactive pairwise potentials revealed that nearly all predict complete droplet spreading, rendering macroscopic contact angles insufficient for model validation. By contrast, our ReaxFF/ACKS2 model, which corrects the spurious water-to-Au charge transfer produced by the default EEM and QEq charge equilibration schemes, resolved finite contact angles of 14.0° for Au(111), 18.1° for Au(001), and 24.1° for Au(110). Complementing these simulations, goniometry on freshly cleaned polycrystalline Au yielded contact angles near 30° that gradually increased to 75° over time in ambient air. By accounting for airborne contamination, the reactive model and the experimental data are brought into quantitative consistency, bracketing the intrinsic wettability of Au from the clean-surface and contaminated-surface sides.

## Graphical abstract

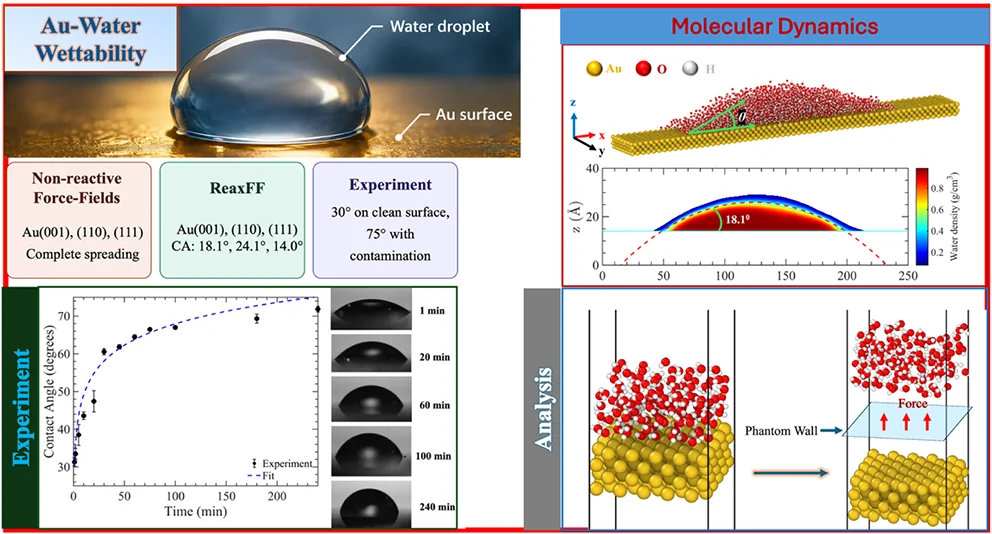

## 1 Introduction

The water wettability of Au has been studied for nearly a century through macroscopic experiments and atomistic simulations, yet a gap between computations and experiments remains. On the experimental side, the historically reported contact angle (CA) spread reflects differences in surface cleanliness, while in simulations it arises from the choice of force-field parameterization, with contributions from droplet size and temperature. The calculated CAs span a wide range from complete wetting (CA ≈ 0°) to hydrophobic behavior (CA > 140°).

Early work by Erb^1^ reported a CA ∼ 62° in 1968, placing Au in the hydrophilic category based on the conventional criterion of CA > 90° for hydrophobicity. Two years later, Bernett and Zisman^2^ reported spontaneous spreading of water on freshly cleaned Au, reaching a different conclusion. In 1980, Smith^3^ compiled twenty-six studies from 1934 to 1980, of which 18 reported hydrophilic behavior (30° < CA < 80°) and eight were hydrophobic outliers. Smith^3^ resolved this disagreement by performing contact angle measurements on a gold film produced via physical vapor deposition (PVD) at room temperature, with surface cleanliness verified by Auger electron spectroscopy before and after each measurement. Results demonstrated that, in Ar environment, the clean PVD film of Au is completely wetted by water; furthermore, a quantitative link between carbonaceous surface contamination and contact angle was introduced: the Auger peak-height C/Au ratio correlated with the measured angle, and less than a monolayer of adsorbed carbonaceous species raised the contact angle from near-zero to approximately 50°. Exposure of clean Au to atmospheric air increased the contact angle to 30–40° within 10 min and to 50–60° within 90 min, confirming that the higher values reported by Erb and others arose from adventitious carbonaceous contamination. This dependence of wettability on the surface density of adsorbed airborne hydrocarbons, an effect known as the “hydrocarbon hypothesis,” has since been confirmed for other materials, including graphene and PTFE,^4,5^ and the more general effect of preadsorbed species competing for surface sites and altering the effective affinity of a solid surface has been documented in confined and porous systems as well.^6^ Chai and Klein^7^ in 2007 reported that template-stripped Au(111) surfaces exhibited initial contact angles of 20–25°. Within minutes of air exposure, the CA rose, presumably due to airborne carbonaceous contaminants adsorbed onto the surface, consistent with Smith’s finding. Turetta et al.^8^ extended this picture in 2021 through a multiscale characterization of template-stripped Au(111) on mica, also tracking contact angle, work function, XPS surface composition, and AFM topography as a function of air exposure time. The CA rose from near-zero on a freshly exposed surface to ∼80° within 2 h, with an increase in the formation of an adventitious carbon adsorption layer. These studies show that the reported range of contact angles on Au under ambient conditions reflects the degree of carbonaceous surface contamination at the time of measurement, rather than the wettability of the bare metal surface.

Throughout this work, we distinguish the intrinsic wettability of Au from its effective wettability. By intrinsic wettability we mean the contact angle that would be established on a bare, atomically ordered Au surface with no adsorbed species present. This is a property of the Au-water interface itself and is the quantity our simulations compute. By effective wettability, we mean the contact angle measured on a real surface, which is set by the combined response of the underlying metal and any adsorbates, steps, grain boundaries, and roughness that the surface carries at the moment of measurement. Every reported experimental contact angle on Au is an effective value. The intrinsic value is not directly measurable and can only be approached by reducing contamination and extrapolating.

On the simulation side, interface modeling is the main challenge to close the computational-experimental gap in Au wettability, and first-principles calculations provide the roadmap to what a model must capture. *Ab initio* molecular dynamics (MD) simulations of the Au(111)-water interface suggest that clean Au is hydrophilic because of the metallic electronic response (enhanced by thermal motion), accompanied by a small oxygen-to-Au charge donation rather than by conventional hydrogen bonding.^9^ Conversely, a recent study combining sum-frequency generation spectroscopy with *ab initio* MD concluded that Au is neither hydrophilic nor hydrophobic in the classical sense.^10^ Its macroscopic wettability arises from orientation-dependent electronic polarization and charge transfer at the interface. Classical nonreactive force fields do not treat the Au-water interface as a single physical system, but as an assembly of three separately developed pieces: a model for the metal, usually an embedded-atom method (EAM) or Lennard-Jones (LJ) description optimized to mimic Au properties;^11^ a separate fixed-charge water model such as SPC/E or TIP4P, parameterized to reproduce bulk liquid water properties;^12,13^ and a cross-interaction term for Au-water, frequently obtained by applying a mixing rule such as Lorentz–Berthelot to the metal and water parameters.^14^ This construction is problematic in two ways. First, the use of mixing rules to set the cross term is itself questionable, since combining rules derived for simple van der Waals gases transfer poorly to metal-water interfaces. Second, stitching three independently developed models together is awkward to implement and difficult to keep internally consistent, and these compounding inconsistencies are a recognized source of the scatter in predicted wettability.^15^ Table 1 summarizes recent computational work using the nonreactive modeling approach. These contributions exhibit a massive variability in predicted contact angles, spanning more than 140° (from 0° to 145°). While physical factors like droplet size, temperature, and initial geometry play a role, we can identify that the source of the Au-water cross-interaction parameters and how they are derived is the dominant factor driving the inconsistencies in the literature.

**Table 1:** Summary of Recent Findings on the Water Wettability of Au Using Nonreactive MD

Study (Year)	Water Model	Au Potential and Cross-Interactions	System Details	CA	Key Findings
Cordeiro and Desai[Bibr ref16] (2017)	TIP3P	CHARMM (Braun et al.^100^); CHARMM combining rules	4 nm (1108 molecules) and 10 nm (17,267 molecules) droplets; 293 K and 373 K	117° (4 nm, 293 K), 145° (10 nm, 293 K), 127° (10 nm, 373 K)	Highly hydrophobic; smaller droplets and higher temperatures decrease CA due to surface-to-volume ratio and H-bond breaking
Foroutan et al.[Bibr ref17] (2017)	TIP4P	EAM (Au–Au); 12–6 LJ (Cross-interactions)	500 molecules 300 K; Au(100)	18°	Highly hydrophilic. Interfacial water matched the Au(100) lattice symmetry with high density
Terzyk et al.[Bibr ref4] (2019)	TIP4*P*/2005	LJ (Heinz et al.^14^); Recalculated LJ for consistency	6000 molecules (cubic lattice); 300 K; Au(100)	0° (Clean), ∼110° (Monolayer *n*-alkane)	Complete wetting on clean Au. Successfully reproduced the contamination-driven transition to hydrophobicity using *n*-alkanes
Aksoy and Bayazitoglu[Bibr ref18] (2024)	PCFF	PCFF 9–6 LJ; Sixth-power mixing rules	1000 molecules; 300 K; Au(100)	67.2° ± 2.7°	Produced an intermediate, partially wetting contact angle utilizing polymer-consistent parameters

A more practical approach is to describe Au, water, and their interaction within a single unified mathematical framework rather than assembling three separate models. The reactive force field ReaxFF embodies this while capturing the charge transfer and polarization that fixed-charge models omit, and that first-principles studies identify as central to the Au-water interface.^19,20^ It is worth clarifying that we adopt ReaxFF here not for its ability to describe bond breaking and formation, since no chemical reactions occur at the Au-water interface under the conditions studied, but for the two features that fixed-charge models lack: a single consistent description of gold, water, and their coupling, and geometry-dependent charges that respond to the local environment. ReaxFF has been used to model Au^21^ and water,^22,23^ and its formulation allows for merging and optimizing these parameter sets for the metal-water interactions. Reactive potentials of this kind have been identified as a promising direction for wettability studies but have not yet been applied systematically to Au.^15^

Because the macroscopic contact angle of water on Au is geometrically unresolved, classical nonreactive force fields lack the mathematical infrastructure to capture the critical quantum-level details of this interface. By benchmarking several established pairwise potentials, we demonstrated the extreme sensitivity of predicted wettability to potential selection, with models yielding solid–liquid adhesion energies that varied by up to a factor of 3 despite all producing full droplet spreading. To resolve this, we developed a ReaxFF parameter set built bottom-up from van der Waals-corrected DFT data, capturing water monomer binding energies, site preferences across the low-index facets, charge transfer, and macroscopic bulk properties of water and Au. This reactive model successfully mimicked the interfacial water structure predicted by *ab initio* MD and yielded resolvable, highly hydrophilic contact angles. Complementing these simulations, contact angle measurements on freshly cleaned Au, taken within one minute of removal from the cleaning bath, minimized adventitious hydrocarbon contamination and placed the effective contact angle near 30°, an upper bound consistent with the clean-facet values from simulation. Ultimately, this bottom-up reactive framework successfully bridges the computational-experimental divide in Au’s wettability.

## 2 Computational Methods

### 2.1 Au-Water Atomistic Model Description

Every droplet wettability MD simulation in this study used the same basic arrangement: an Au slab with a film of water resting on top, placed inside a periodic simulation cell. The slabs were cut from the face-centered cubic (FCC) structure of bulk Au along three low-index planes, exposing the (001), (110), and (111) surfaces. Working with all three facets allows us to investigate the previously reported wettability anisotropy.^24–26^ Figure 1 depicts the Au(001) surface, with the starting configuration in panel (a) and the relaxed droplet after 4 ns of simulation in panel (b). The same Au-water models were used for both the nonreactive force-field calculations in Section 2.2 and the ReaxFF calculations in Section 2.3.

**Figure 1:**
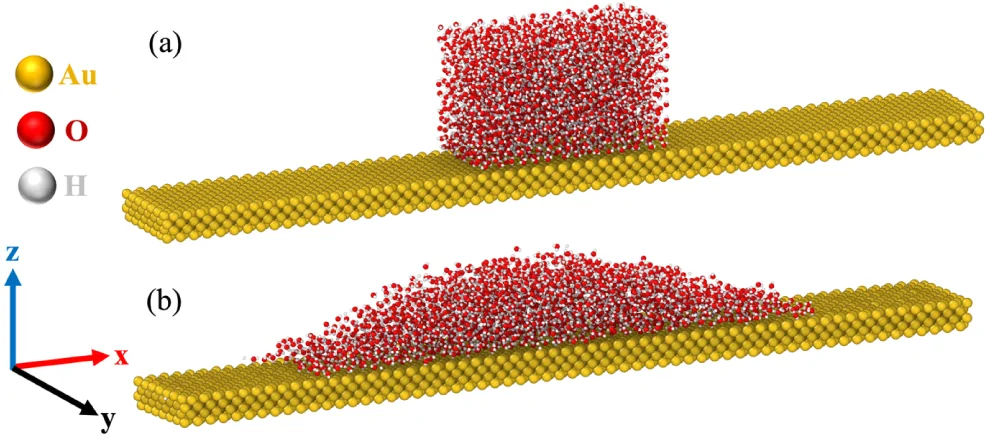
Representative atomistic model of the Au(001)-water system used for the contact-angle simulations. (a) Initial configuration and (b) the relaxed cylindrical droplet after 4 ns of ReaxFF MD simulation at 300 K. The Au slab measures approximately 250 × 29 × 8.2 Å along *x, y*, and *z*.

Each slab is four atomic layers thick; the box dimensions and atom counts differ between the three facets because each plane has a different surface atomic density and interlayer spacing; see Table 2 for details. In all three cases, the slab is roughly 250 Å long in the *x-*direction, close to 30 Å wide in the *y*-direction, and nearly 7 to 8 Å thick. The large length in the *x*-direction allows the droplet to spread and form a stable cap, while the short *y*-dimension promotes the formation of a straight contact line (see Figure 1 (b)). During the simulations, the slab’s bottommost atomic layer was anchored to prevent drifting or deformation. This setup is a cylindrical droplet model, and it has several advantages over the alternative spherical nanodroplet. Because the droplet relaxes into a periodic cylinder, the contact line stays straight (infinite radius of curvature), and the line-tension correction to Young’s equation vanishes. Consequently, size effects are less pronounced, extrapolation to the thermodynamic limit is more cost-effective, and fewer atoms are necessary to produce statistically meaningful contact angles.^27,28^

**Table 2:** Dimensions and Composition of the Au(001), Au(110), and Au(111) Slab-and-Water Systems Used for the Contact-Angle Simulations

	Au Slab Dimensions (Å)				
	x	y	z	Number of Au Atoms	Number of Water Molecules
Au(001)	248.88	28.56	8.16	3416	2140
Au(110)	250.99	28.56	8.66	2436	2140
Au(111)	250.99	29.98	7.07	4176	2100

A water film was placed on top of the Au slab, centered along the *x*-axis, with an initial gap of 3 Å between the water and the surface to keep atoms from overlapping. The film spanned 50 Å in the *x*-direction, matched the width of the Au slab in the *y*-direction, and stood 45 Å tall in the *z*-direction. Each system held roughly 2,100 to 2,140 water molecules, depending on the facet. Periodic boundary conditions were applied along the *x-* and *y*-directions, while a large vacuum region was kept above the water film along the *z*-direction.

### 2.2 Nonreactive MD Benchmarking

The consequences of inconsistent interface modeling practices were investigated by Paniagua-Guerra and Ramos-Alvarado,^29^ who compared nine Au-water interaction parameters from the literature. They found that the calculated thermal boundary conductance varied by up to a factor of 5 across the sets, while all interface force fields were assumed to produce a hydrophilic interface. Nonetheless, interfaces that exhibit the same wetting properties can differ widely in their underlying physics, e.g., interfacial water structure and adhesion energy, a consequence of inconsistent interface force field parameterization.^15,30^ The nine nonreactive pairwise force fields (FF1 to FF9) drawn from the previous work of Paniagua-Guerra and Ramos-Alvarado^29^ are used to model Au-water interactions in this study; their parameters are listed in Table 3. The set spans four functional forms: the 12–6 Lennard-Jones (LJ), 9–6 LJ, Buckingham, and Morse potentials. Au–Au interactions are described throughout by the embedded-atom method (EAM) of Foiles et al.,^11^ fitted to the sublimation energy, lattice constant, elastic constants, and vacancy formation energy of FCC metals. Water is represented by the extended simple point charge (SPC/E) model^12^ in all cases. SPC/E was selected for its accurate bulk water properties and its established use in prior Au-water interface studies, which facilitates direct cross-comparison.

**Table 3:** Nonreactive Au-Water Force-Field Parameters (FF1 to FF9) Used in This Study, with Their Functional Form, Parameterization Target, and Original Source^a^

FF	Functional Form	Parameters	Parameterization Target	Source	CA Reported
FF1	LJ 12–6 (Au–O only)	*ε*_AuO_ = 2.4686 kJ/mol; σ_AuO_ = 3.60 Å	Target CA = 25° (SPC/E surface tension)	*Merabia et al.* ^31^	No (used as input)
FF2	LJ 12–6 (Au–O only)	*ε*_AuO_ = 8.28 kJ/mol; σ_AuO_ = 2.95 Å	Exp. adsorption energy	*Tay and Bresme* ^32^	No (NP only)
FF3	LJ 12–6 (Au–O, LB rules)	*ε*_AuO_ = 3.7924 kJ/mol; σ_AuO_ = 2.885 Å	Surface tension + Lorentz–Berthelot rules	*Heinz et al.* ^14^	No
FF4	LJ 12–6 (Au–O only)	*ε*_AuO_ = 3.8792 kJ/mol; σ_AuO_ = 2.8491 Å	DFT vdW-DF2 binding curve	*Gim et al.* ^33^	θ = 0°
FF5	LJ 12–6 (Au–O + Au–H)	ε_AuO_ = 3.6100 kJ/mol; σ_AuO_ = 2.833 Å; *ε*_AuH_ = 0.01 kJ/mol; σ_AuH_ = 3.5190 Å	vdW-DFT adsorption on Au(111)	*Berg et al.* ^34^	No
FF6	LJ 9–6 (Au–O + Au–H)	*ε*_AuO_ = 2.91 kJ/mol; σ_AuO_ = 2.9352 Å; *ε*_AuH_ = 0.05 kJ/mol; σ_AuH_ = 3.2760 Å	vdW-DFT adsorption on Au(111)	*Berg et al.* ^34^	No
FF7	Buckingham (Au–O + Au–H)	*A*_O_ = 45636 kJ/mol; ρ_O_ = 0.43 Å; *C*_O_ = 0.027 kJ nm^6^/mol	vdW-DFT adsorption on Au(111)	*Berg et al.* ^34^	No
FF8	Morse (Au–O + Au–H)	*D*_O_ = 1.86 kJ/mol; α_O_ = 0.905 Å^–1^; *r*_0,O_ = 4.20 Å	vdW-DFT adsorption on Au(111)	*Berg et al.* ^34^	No
FF9	Morse + BMH (Au–O + Au–H)	*D*_O_ = 6.4441 kJ/mol; α_O_ = 1.3 Å^–1^; *r*_0,O_ = 3.7 Å; *A*_H_ = 1.289 kJ/mol; ρ_H_ = 0.3846 Å	Exp. adsorption energy + site corrugation	*Dou et al.* ^35^	No

a BMH = Born-Mayer-Huggins; LB = Lorentz-Berthelot combining rules; NP = nanoparticle.

The literature on wettability for these nine potentials is limited. None of FF1 to FF9 was explicitly validated against a contact angle on a flat crystallographic Au surface. In the present work, all nine force fields were applied to flat Au(001), Au(110), and Au(111) slabs using the cylindrical droplet method on surface models as described in Section 2.1. An error was identified in the tabulation of FF6 parameters by Paniagua-Guerra and Ramos-Alvarado.^29^ The 9–6 LJ potential defines its characteristic length through r_m_ (the position of the energy minimum), which relates to the zero-crossing distance σ as σ = r_m_/(3/2)^1/3^. The published tabulation applied the 12–6 relationship, σ = r_m_/2^1/6^ instead, producing σ values approximately 1.98% larger than the correct source values (σ_AuO_ = 2.9934 Å and σ_AuH_ = 3.3409 Å, versus the corrected values of σ_AuO_ = 2.9352 Å and σ_AuH_ = 3.2760 Å). All simulations reported in this work used the corrected parameters taken directly from Berg et al.^34^ and listed in Table 3.

Further details of these nonreactive Au-water force-field parameters, including the Lennard-Jones 12–6 potentials with Au–O interactions (FF1 to FF4) and the multisite potentials with Au–O and Au–H interactions (FF5 to FF9), together with the corresponding LJ 12–6 interaction-energy expression, are provided in Supporting Information Sections S1.1 and S1.2.

All simulations with each of the above-mentioned force fields are performed using the open-source code LAMMPS (22Jul2025 version).^36^ The Au slab and water film are initially relaxed by energy minimization, applying a steepest-descent algorithm (force and energy tolerances of 1.0 × 10^–4^ and 1.0 × 10^–6^, respectively), followed by conjugate-gradient minimization (tolerances of 1.0 × 10^–6^ and 1.0 × 10^–8^) to remove residual atomic overlaps. Following minimization, the system is equilibrated under the canonical (NVT) ensemble at 300 K for 4 ns using a Nosé-Hoover thermostat with a damping constant of 100 fs and a time step of 1.0 fs. The bottom layer of the Au slab is held fixed throughout to prevent slab translation, and rigid SPC/E water geometry is enforced via the SHAKE algorithm.^37^

Production data are collected over a subsequent 2 ns run under the microcanonical (NVE) ensemble at the same time step. During production, the Au-water interaction energy is sampled every 1000 steps using the LAMMPS group/group compute. Atomic trajectories are saved every 5000 steps for visual inspection of the wetting state and for subsequent contact angle calculation.

The contact angle on each surface was calculated from the equilibrated cylindrical droplet using the multiresponse postprocessing method of Ramos-Alvarado et al.,^27^ applied to the water trajectories from the production runs. Because the cell is periodic and short along the *y-*direction, the droplet relaxes into a cylinder with its cross-section in the *x-z* plane, which carries all the contact angle information. The time-averaged mass density field ρ­(*x*, *z*) was accumulated over the analysis window on a uniform 0.25 × 0.25 Å grid in the *x-z* plane, with the full *y*-extent collapsed into the bin depth, a resolution fine enough to resolve the interfacial layering while leaving the bulk region smooth. The bulk liquid density ρ_*L*_ was obtained from a sigmoidal fit to the density profile, and the liquid–vapor interface was located as the contour on which the field falls to ρ_*L/2*_, with linear interpolation between adjacent bins so that the interface points are not locked to the grid. The strongly layered region within ∼5 Å of the Au was discarded because there the local density oscillates well above and below the bulk value, so the half-density contour does not define a liquid–vapor interface and the profile departs from the circular form assumed by the Young analysis. The sensitivity of the extracted angle to this cutoff is documented in Section S7 of the Supporting Information. The remaining interface points were fitted to a circle by least-squares. The macroscopic contact angle is the angle between the tangent to that circle at its intersection with the Au surface and the surface itself, measured through the liquid.

### 2.3 Au-Water ReaxFF Force Field Development

The ReaxFF reactive force field was chosen to describe the Au-water interface because it can (1) model dynamic charge transfer and polarization, allowing the electronegativity and partial charges of the atoms to update dynamically based on their local environment, and (2) eliminate the need for mixing rules as it relies on bond-order potentials that are trained directly against quantum-mechanical (DFT) data for specific molecular interactions, all within a single mathematical framework with the chemical flexibility of a quantum-mechanical description while running at a cost suitable for nanoscale interfacial systems.^19,38^

An initial Au-water force field was built by combining two existing ReaxFF parameter sets. The water parameters were taken from the ReaxFF water description used by Achtyl et al.,^23^ developed in the context of their study of aqueous proton transfer across single-layer graphene. This parameterization’s main advantage is that it treats water as a reactive, polarizable species rather than as a rigid molecule with fixed point charges, which is consistent with the reactive description of the Au surface and allows the polarization and short-range structure of the first hydration layer to be captured. The collective water–water interactions that govern the cohesion and shape of the droplet are therefore described by an established liquid-water parameter set rather than by anything inferred from single-molecule adsorption data. The Au parameters were taken from the ReaxFF Au force field of Keith et al.,^21^ who fitted the parameters to PBE-GGA DFT data covering bulk FCC Au (e.g., lattice constant, cohesive energy, and equation of state), the low-index Au surfaces Au(111), Au(100), and Au(110) (surface energies and relaxations), and small Au nanoparticles of icosahedral and cuboctahedral shape. The force field reproduces the metallic bonding of Au, the energetic ordering of the low-index facets, and the size-dependent stability of Au nanoparticles, which makes it a good choice for slab geometries of the type used here.

Combining the aforementioned water and Au parameters gave a usable starting point, but the Au-water cross terms had to be trained further and were the only terms optimized in this work. The reference data for this training came from the vdW-corrected DFT study of Berg, Peter, and Johnston,^34^ who carried out a systematic evaluation and optimization of interface force fields for water on Au surfaces. They computed these interaction energies with plane-wave DFT in Quantum ESPRESSO, using the nonlocal van der Waals density functional vdW-DF, which incorporates dispersion self-consistently rather than through an added empirical correction, with a plane-wave cutoff of 50 Ry and ultrasoft pseudopotentials. Their data set comprises 174 water configurations on four-layer Au(001) and Au(111) slabs, spanning the top, bridge, and hollow sites with the molecule oriented with either the oxygen or one or both hydrogens directed toward the surface. Dispersion is essential at this interface: the same authors report that the vdW-DF interaction is substantially stronger than PBE without dispersion and places the equilibrium distance farther from the surface. For the present training, we focused on the configurations that most directly control the structure and adhesion of the first hydration layer, i.e., water adsorbed with the hydrogens pointing toward the surface (H-down) and away from the surface (H-up), and water with its oxygen sitting above the top, bridge, and hollow sites of Au(001) and Au(111).

The parameters optimized here are Au–O and Au–H pairwise terms together with the associated charge-transfer parameters, and are therefore defined between atom types rather than for particular surface orientations. A ReaxFF parameter set contains no facet-specific entries, and facet dependence emerges instead from the surface geometry, the atomic surface density, and the coordination of the surface atoms, all of which the same pairwise terms describe. The Au(001) and Au(111) configurations of Berg et al.^34^ sample the local coordination environments these terms set, and the resulting parameter set is applied unchanged to Au(110), which was not represented in the training data. The Au(110) results in Section 3.2 are therefore predictions of the model, and their consistency with the surface-energy and work-of-adhesion orderings provides a test of transferability to a more open surface.

In its default implementation, ReaxFF updates atomic partial charges at every MD step using the electronegativity equalization method (EEM). This model exhibited excessive charge transfer from the water film to the Au (∼153 e within the first 100 ps), whereas a neutral Au surface in contact with water would be expected. The charged surface pulled the water down and yielded complete wetting, which is an artifact of the charge scheme rather than a feature of the true Au-water interaction. The origin of the problem lies in how EEM treats interatomic charge response. Each atom in EEM has an electronegativity and a hardness, and the partial charges at every step are obtained by solving an n × n linear system, where n is the number of atoms. The hardness is treated as a purely local atomic property and does not depend on interatomic distance, so charge can in principle flow from any atom to any other atom without any distance-dependent resistance. In a heterogeneous system such as Au and water, where the Au and oxygen electronegativities differ substantially, this allows the water to lose a sizable amount of charge to the metal slab without paying any spatial cost.

To address this, we switched to the ACKS2 charge equilibration scheme of Verstraelen et al.^39^ ACKS2 is derived as an atom-condensed second-order approximation to Kohn–Sham DFT, and the key change relative to EEM is that it adds a distance-dependent contribution to the charge-response kernel. In effect, the hardness becomes distance-dependent, i.e., the cost of moving charge between two atoms grows with their separation, which damps out the long-range charge transfer that EEM allows. The Achtyl et al.^23^ water force field was developed using the ACKS2 method, which is why we chose this particular ReaxFF water description. With ACKS2, the Au slab remained essentially neutral throughout the wettability simulations on all three facets. The additional degree of freedom per atom makes the charge solve considerably more expensive than EEM, an overhead that is discussed in Section 6.

To quantify the difference between the two charge-equilibration schemes, per-atom charges were extracted directly from the production trajectories of the Au(001)-water system. Averaged over the ACKS2 run, the Au slab carries a time-averaged charge of +9.70 ± 0.45 e, balanced by −9.70 ± 0.45 e on the water, corresponding to +0.0028 ± 0.0001 e per Au atom and −0.0045 e per water molecule. The equivalent EEM values, obtained from the first 100 ps before that simulation was discontinued, are +152.25 ± 4.85 e and −152.25 ± 4.85 e, or +0.0446 ± 0.0014 e per Au atom, more than an order of magnitude larger. Resolving the ACKS2 charge by atomic layer shows that the residual charge is confined to the water-facing Au layer, which carries +9.85 e or +0.0115 e per atom, while the 2562 atoms of the three subsurface layers carry −0.15 e in total, or −0.00006 e per atom. The interior of the slab is therefore neutral to within the resolution of the analysis, and the induced charge is screened within a single atomic layer, as expected for a metal in contact with a polar liquid. Normalized over the full Au surface area, the interfacial charge density is 0.0014 e Å^–2^, or 2.19 μC cm^–2^, rising to approximately 5.8 μC cm^–2^ when normalized over the wetted area beneath the droplet. Figure 2 depicts the charge spatial distribution, in which the Au atoms remain essentially neutral across the full width of the slab, including the dry regions on either side of the droplet, while the charge resides on the water and is most ordered in the contact layer immediately above the surface.

**Figure 2:**
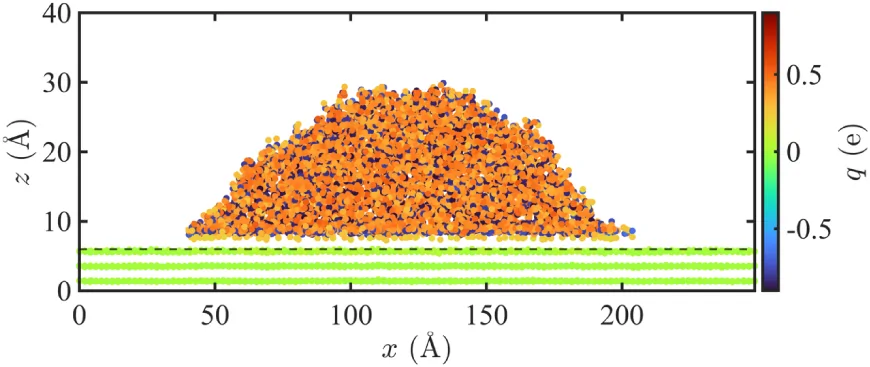
Instantaneous atomic charges at the Au(001)-water interface, taken from the ACKS2 production trajectory at *t* = 4 ns. Color indicates the charge on each atom, with the range set by the water. The dashed line marks the topmost Au layer. Gold atoms across the entire slab, including the dry regions on either side of the droplet, sit at the center of the color scale, showing that the metal remains neutral while the charge resides on the water.

For brevity, we use ReaxFF and ReaxFF/ACKS2 interchangeably throughout the manuscript to refer to our Au-water model, which incorporates the ACKS2 charge-equilibration scheme unless otherwise specified.

The optimization of these cross terms minimized a cumulative normalized error between the ReaxFF predictions and the vdW-DFT reference data. The error function had the standard ReaxFF form:

1 $$Error=\overset{n}{\underset{i=1}{\sum }}{[\frac{x_{i,QM}-x_{i,ReaxFF}}{\sigma_{i}}]}^{2}$$

where *x*_*i*,QM_ is the reference DFT value, *x*_*i*,ReaxFF_ is the corresponding ReaxFF prediction, *n* is the total number of training points, and *σ_i_* is the accuracy target or weighting factor assigned to each data point. Smaller values of *σ_i_* give greater weight to a data point during optimization. The final parameter set represents the best global compromise across all training data rather than an exact fit to every individual point. The final ReaxFF force field parameters can be found in Section S10 of the Supporting Information.

Figure 3 compares the trained ReaxFF/ACKS2 model with the vdW-DFT reference. The optimized force field reproduces the main features of the adsorption curves for both H-up and H-down orientations, as illustrated in Figure 3a and 3b, and follows the DFT energy trends for water over top, bridge, and hollow sites on Au(001) and Au(111), depicted in Figure 3c and 3d. The level of agreement seen in Figure 3 indicates that the trained Au-water parameters provide a reasonable description of the short-range interaction between water and clean Au surfaces.

**Figure 3:**
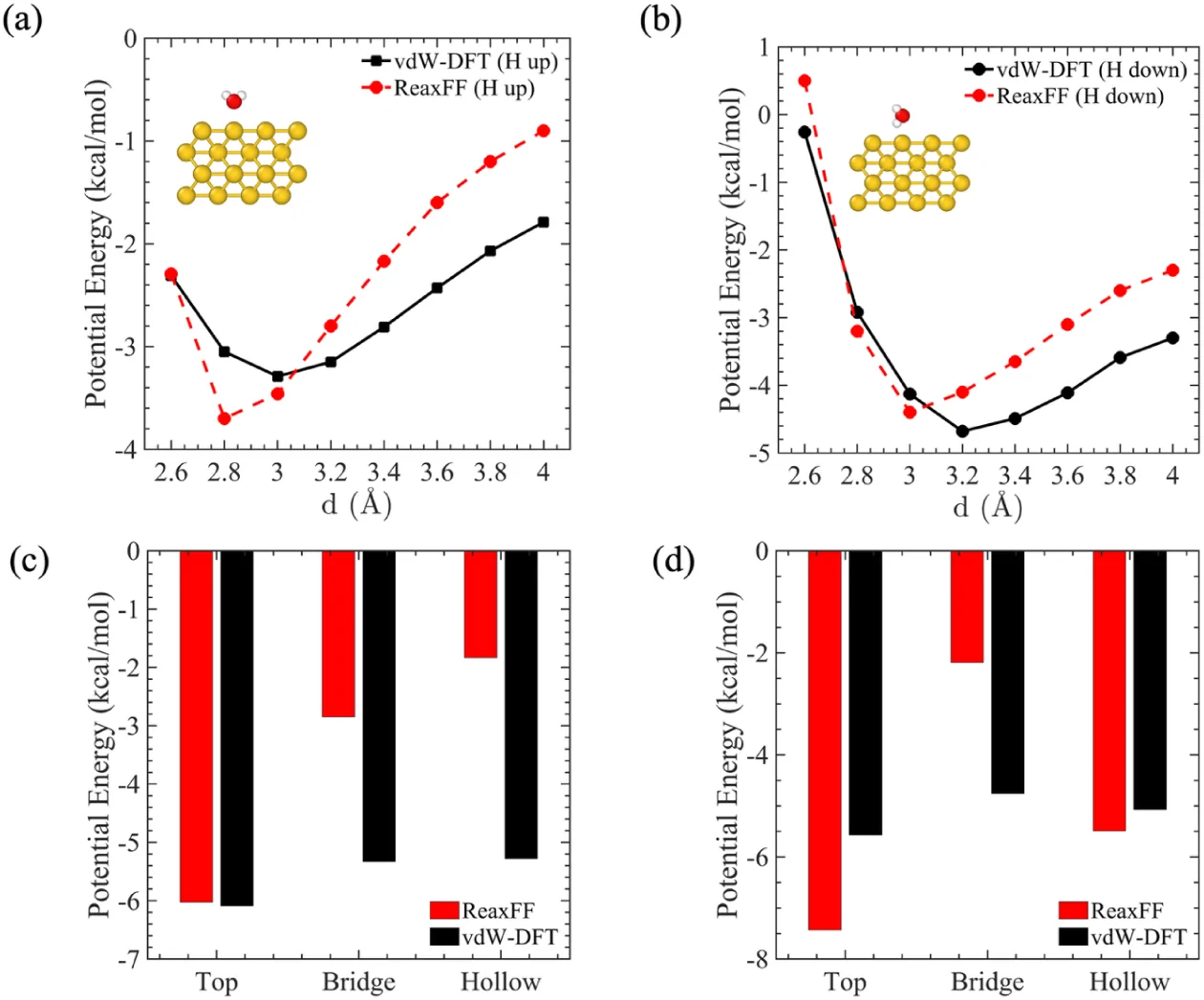
Comparison of ReaxFF-predicted and DFT-calculated interaction energy profiles for water on Au surfaces in different adsorption configurations. Panels (a) and (b) show the training results for H-up and H-down water orientations, respectively. Panels (c) and (d) show the fitted energy profiles for water adsorbed with the oxygen atom positioned above top, bridge, and hollow sites on Au(001) and Au(111), respectively. The DFT reference data were taken from Berg et al.,^34^ and the optimized ReaxFF parameters were trained to reproduce the relative adsorption energies and equilibrium interaction distances relevant to Au-water wettability.

A detailed comparison of the ReaxFF/ACKS2 water description against the nonreactive SPC/E and TIP3P models and experimental data (Figures S1 and S2), together with validation of the ReaxFF/ACKS2 description of Au against experimental thermal-expansion and cohesive-energy data (Figure S3), is provided in Supporting Information Sections S2 and S3.

The ReaxFF/ACKS2 wettability simulations used the same setup as the nonreactive runs in Section 2.2, with a few changes required by the reactive description. The same LAMMPS build^36^ and the same two-stage energy minimization (steepest descent followed by conjugate gradient) were applied, and each system was then equilibrated in the NVT ensemble at 300 K for 4 ns with the bottom Au layer held fixed. Water was modeled as a fully reactive, flexible species, so the rigid-geometry SHAKE constraint of the SPC/E runs was dropped, and the atomic partial charges were updated at every step through the ACKS2 scheme introduced above. The time step was shortened to 0.25 fs, as commonly used for low-temperature ReaxFF simulations.^38^ Atomic trajectories and velocities were stored every 500 steps for the wetting-state analysis. The contact angle on each facet was then extracted from these trajectories with the cylindrical droplet model and the multiresponse postprocessing method of Ramos-Alvarado et al.,^27^ discarding the strongly layered region within about 5 Å of the Au before the circular fit, exactly as in Section 2.2. The binning and density-fitting details are collected in Section S7 of the Supporting Information.

For reference, Table 4 summarizes the key simulation parameters used in both the nonreactive and ReaxFF/ACKS2 simulations.

**Table 4:** Summary of Key Simulation Parameters for the Nonreactive and Reactive Simulations

Parameter	Nonreactive (SPC/E) Simulations	ReaxFF/ACKS2 Simulations
Software	LAMMPS	LAMMPS
Energy minimization	Steepest-descent → conjugate-gradient	Same
Equilibration ensemble	NVT, 300 K, 4 ns	Same
Thermostat	Nosé-Hoover, 100 fs damping	Same
Equilibration time step	1.0 fs	0.25 fs
Boundary condition	Bottom Au layer fixed	Same
Charge equilibration	Fixed SPC/E charges	ACKS2, updated every MD step
Nonbonded cutoff	15.0 Å	10 Å

## 3 Computational Results and Discussion

### 3.1 Nonreactive Force Field Wettability Calculations

Figure 4 depicts the equilibrated state of a water droplet on Au(001) at the end of a 6 ns simulation (4 ns NVT followed by 2 ns NVE production) for each of the nine nonreactive force fields. Eight of the nine nonreactive force fields produce complete wetting on all three Au surface orientations, with the initial cylindrical droplet spreading into a uniform film during the 4 ns NVT equilibration. This behavior confirms that complete spreading is the dominant outcome for nonreactive SPC/E-EAM descriptions of the Au-water interface, regardless of parameterization strategy. The only exception is FF5, which retains a finite droplet on Au(001) and Au(110), as illustrated in Figure 4(e) and S4.i (e); FF5 produced complete wetting on Au(111). To rule out the kinetic artifact of insufficient equilibration time, the FF5 simulations on Au(001) and Au(110) were extended to 8 ns, and the droplet persisted on both surfaces (see Figures 4(f) and S4.i­(f)). Equilibrated snapshots for the Au(110) and Au(111) surfaces are provided in Supporting Information, Section S4 (Figure S4).

**Figure 4:**
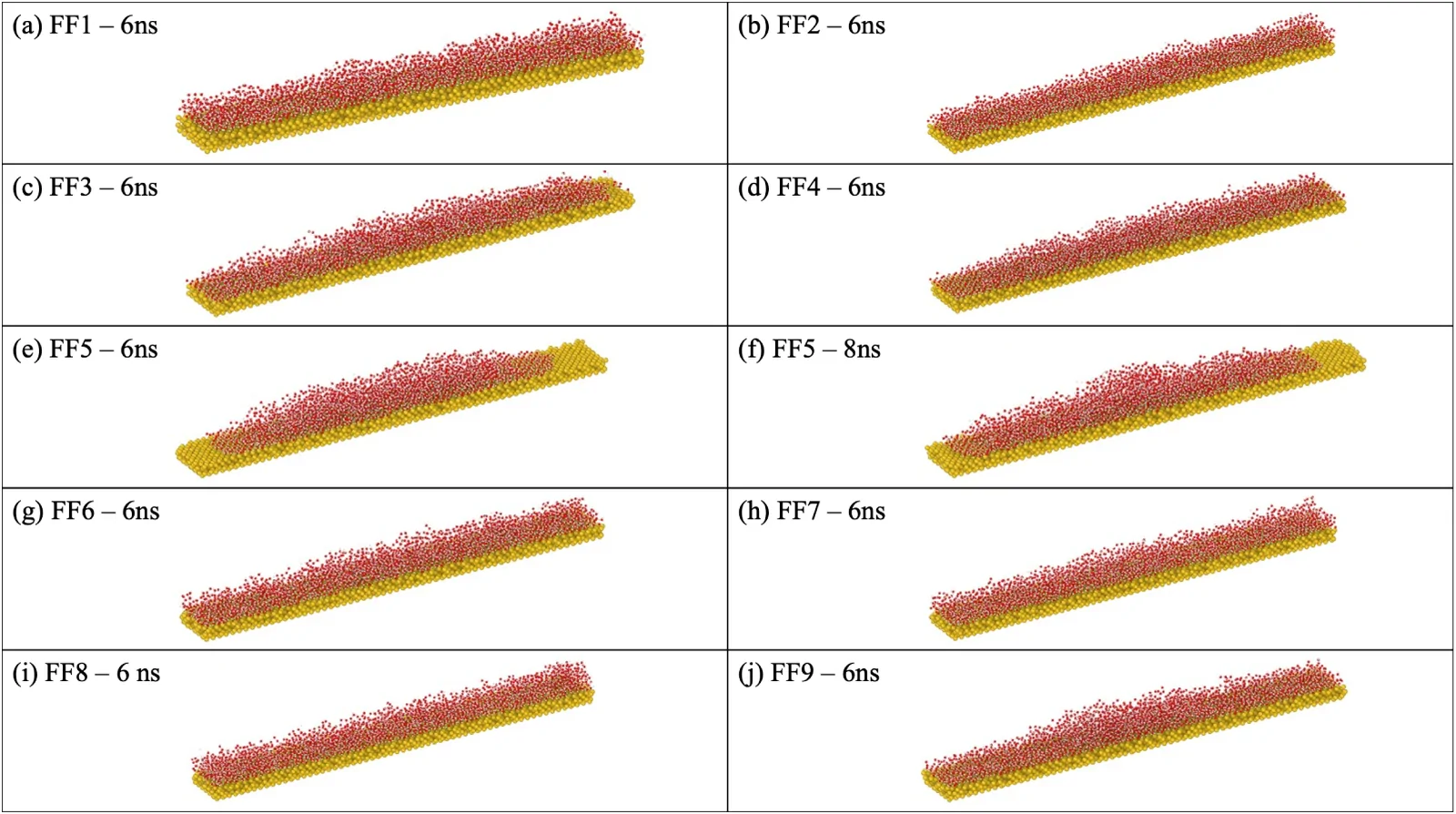
Representative simulation snapshots for all nine nonreactive force fields on the Au(001) surface at *t* = 6 ns, or 4 ns NVT plus 2 ns NVE. Au atoms are shown in yellow, oxygen in red, and hydrogen in white. A flat-water film indicates complete wetting (CA ∼ 0°). FF5 is the only parameterization that shows partial wetting.

FF5 retains a finite droplet because it carries the weakest Au-water interaction energy of the nine parameterizations, with values of −1663.5 ± 10.9 kJ mol^–1^ nm^–2^ on Au(001) and −1532.8 ± 11.9 kJ mol^–1^ nm^–2^ on Au(110), as detailed in Section S5. The weaker Au-water attraction relative to water–water cohesion is the cause of partial wetting in this case, not a consequence of it. Circular-arc fitting to the time-averaged density field (Section S6) gives a contact angle of 10.96° on Au(001) and 11.20° on Au(110), placing FF5 in the strongly hydrophilic regime even where wetting is incomplete.

Among the eight force fields that produce complete wetting, FF1 is unique in that it is the only parameterization derived from an explicit wettability target. Its Au–O well depth (ε = 2.4686 kJ/mol) was originally tuned to get a target contact angle of 25° using an analytical estimate based on LJ contributions to the surface tensions and the SPC/E liquid–vapor surface tension at 300 K, but that estimate was never tested by simulation in the original work. Applied here to a flat crystallographic surface, FF1 produces complete spreading rather than the targeted finite contact angle, the same outcome as the other seven complete-wetting force fields despite its different parameterization strategy.

### 3.2 ReaxFF Wettability Calculations

This section presents the wettability characterization of the Au(001), Au(110), and Au(111) facets using the newly developed ReaxFF/ACKS2 parameter set. For each surface, we built a time-averaged water density field, extracted the macroscopic contact angle, confirmed its convergence with a set of quality diagnostics, and then computed the work of adhesion and the clean-surface energy so that the wettability of the three facets could be explained on a common footing.

To verify CA calculation convergence, the analysis was run cumulatively, and four diagnostics were tracked together as a function of analysis time. Averaging was started from five reference times, *t*_start_ = 1.0, 1.5, 2.0, 2.5, and 3.0 ns, following the multistart-time test described in the Supporting Information of Ramos-Alvarado et al.,^27^ and for each start time, the CA, the centerline bulk density, the root-mean-square error (RMSE) of the circular fit, and the RMSE of the sigmoidal fit were recomputed as frames accumulated. A result was accepted as converged only when (1) multiple contact-angle curves collapsed onto a common value, (2) the two RMSE indicators decayed to small and slowly varying levels, and (3) the circular-fit RMSE fell to or below the 0.3 Å threshold recommended by Ramos-Alvarado et al.^27^ Full details are given in Section S7 of the Supporting Information.

Figure 5 illustrates these diagnostics for the Au(001) system as a representative case. In panel (a), the contact-angle curves begin in an oscillatory regime, with the t_start_ = 1.0 ns curve overshooting to ∼26° because it still includes frames that are settling toward equilibrium and then collapse onto a common plateau near 18° by the end of the window. Panel (b) depicts the circular-fit RMSE decaying from several angstroms toward the 0.3 Å threshold for every start time. The simultaneous flattening of the angle in panel (a) and the decay of the fit error in panel (b) are what make the result trustworthy, since a stable angle paired with a small and falling fit error is hard to obtain by chance. Panel (c) shows the centerline bulk density settling near 0.98 g/cm^3^, a little below the experimental value of liquid water and consistent with the slightly low ReaxFF water density established earlier, and panel (d) details the density-fit RMSE falling well below 0.1 g/cm^3^. Starting at 2.0 ns or later removes the settling frames, and the remaining curves are almost indistinguishable. The production analysis window was therefore fixed at *t* = 2.0 to 4.0 ns for all three facets, containing 16,000 stored frames per system at the 0.25 fs time step and trajectory output frequency given in Section 2.3. The corresponding diagnostics for Au(110) and Au(111) are depicted in Figures S9 and S10.

**Figure 5:**
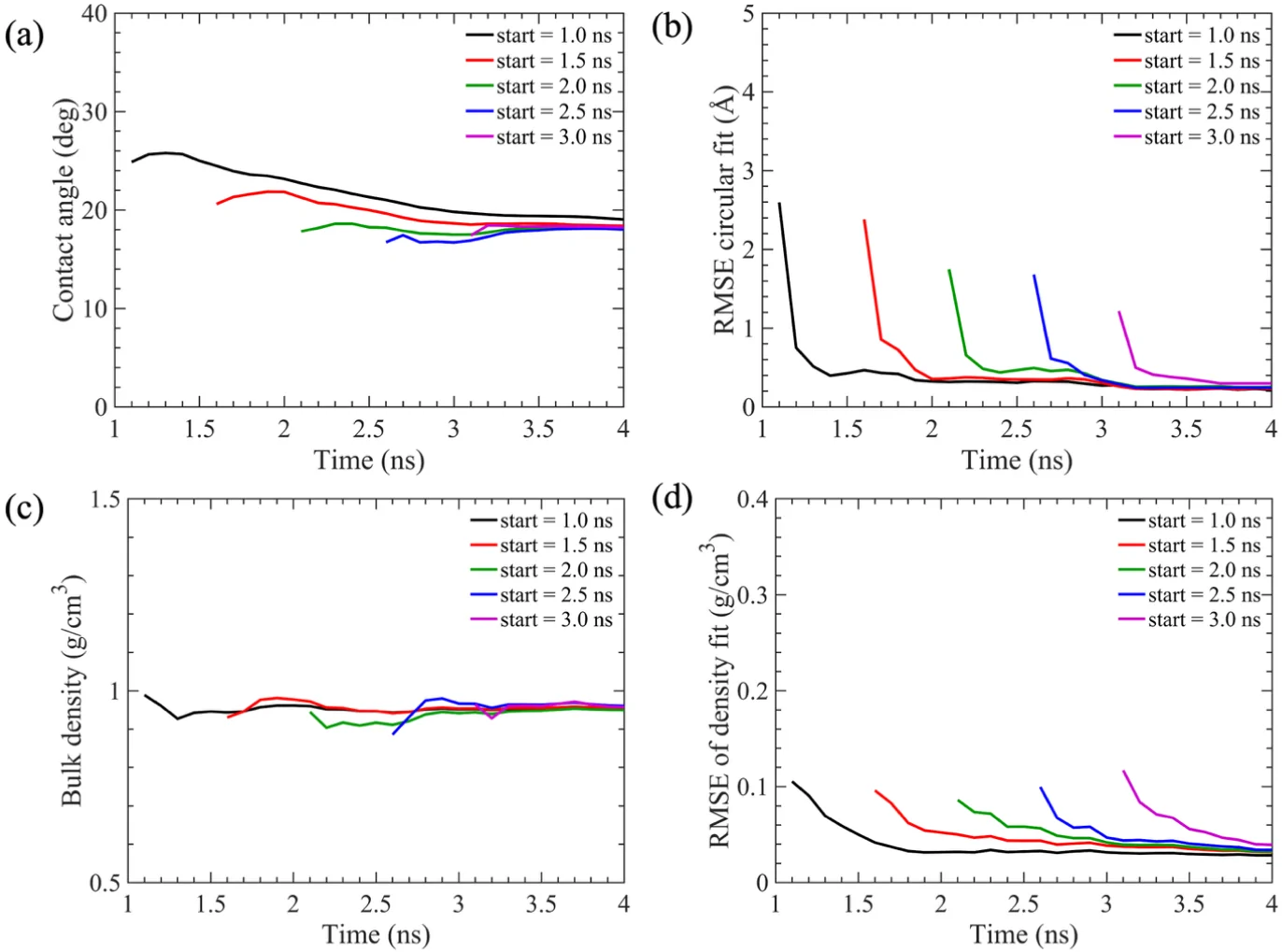
Convergence diagnostics of the contact-angle analysis for the Au(001) system, shown as cumulative averages from five start times (*t*_start_ = 1.0 to 3.0 ns). (a) Cumulative contact angle, (b) root-mean-square error (RMSE) of the circular fit, (c) cumulative centerline bulk density, and (d) RMSE of the sigmoidal density-profile fit.

Figure 6 collects the results for the three facets. Panels (a) to (c) depict the time-averaged density contours with the half-density interface and the fitted circle, resulting in CAs of 18.1° for Au(001), 24.1° for Au(110), and 14.0° for Au(111). All three lie well inside the hydrophilic regime. Panel (d) details the centerline density profiles, which share the same qualitative shape: a sharp first contact peak near 2.5 Å reaching about 2.5 g/cm^3^, one or two weaker layering peaks, and a decay to a bulk plateau near 1.0 g/cm^3^ within roughly 10 Å of the surface. The position and height of the first peak are almost the same for all three facets, which indicates that the short-range Au-water binding is set mainly by direct oxygen-Au contacts rather than by the in-plane arrangement of the surface atoms. Beyond the first layer, the profiles separate, i.e., the Au(110) droplet carries its liquid plateau out to roughly 20 Å before dropping to vapor, while Au(001) and Au(111) reach the bulk plateau sooner, which is consistent with the taller, more beaded droplet expected for the higher contact angle of Au(110).

**Figure 6:**
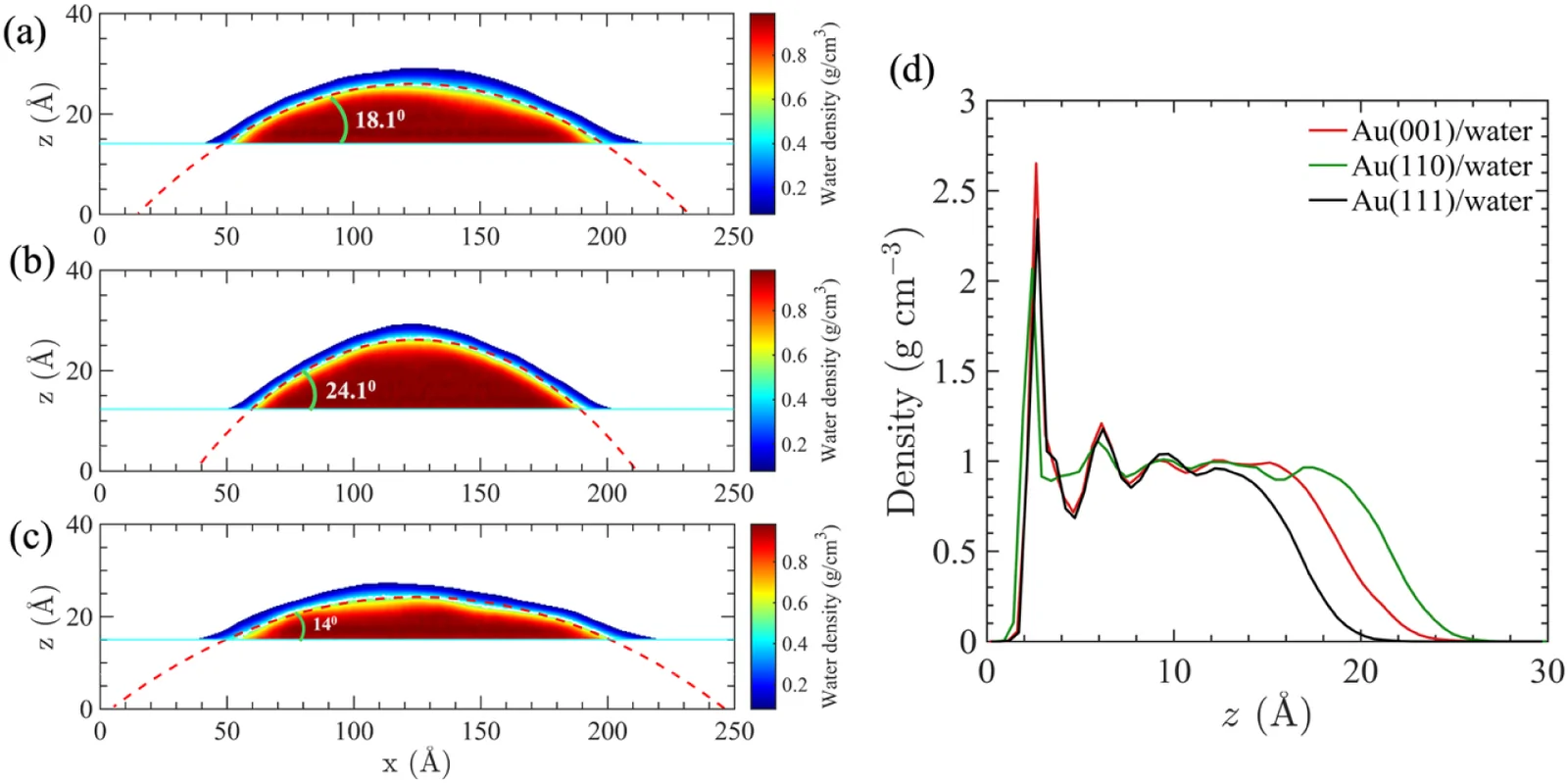
Contact-angle results for the three Au facets from the ReaxFF/ACKS2 simulations. Time-averaged water density contours and the fitted circle, giving contact angles of (a) 18.1° for Au(001), (b) 24.1° for Au(110), and (c) 14.0° for Au(111). (d) Centerline water density profiles for Au(001), Au(110), and Au(111).

#### 3.2.1 Work of Adhesion and Surface Energy

To examine whether the facet dependence of the contact angle reflects a corresponding difference in solid–liquid affinity, we computed the work of adhesion *W*_*ad*_ between water and each Au surface using the phantom-wall method of Leroy and Müller-Plathe,^40^ which computes the reversible work needed to separate the water from the solid by thermodynamic integration along a quasi-static mechanical path and accounts for both the energetic and entropic contributions to adhesion at finite temperature. These calculations used a separate set of flat slab-on-slab systems, one per facet, rather than the cylindrical droplets used for the contact angle. The planar geometry is required by the method, since the work is normalized per unit area and a curved droplet interface would introduce curvature and contact-line contributions into a quantity defined for a flat interface. These calculations are therefore not an independent determination of the contact angle; they provide a separate thermodynamic measure of Au-water affinity against which the ordering obtained from the droplet simulations can be compared. Simulations for each facet were performed three times with different initial velocity seeds, giving 125.16 ± 9.31 mJ/m^2^, 111.24 ± 8.44 mJ/m^2^, and 100.35 ± 7.32 mJ/m^2^ for Au(111), Au(001), and Au(110), respectively.

We also computed the clean-surface energy of each facet, using a slab energy-difference method in which a fully periodic Au crystal and the same slab with a vacuum gap are each relaxed, and the energy difference is divided by the created surface area. Both the slab and the periodic reference were constructed at the ReaxFF equilibrium lattice constant of a_o_ = 4.1653 Å, obtained by relaxing a fully periodic bulk cell, rather than at the experimental value, so that the slab is unstrained with respect to the force field’s own equilibrium geometry. The simulation setup, the convergence tests, and the governing equations for both the work of adhesion and the surface energy are described in Section S8 of the Supporting Information. The computed surface energies of 1.68, 1.89, and 2.00 J/m^2^ follow the order γ(111) < γ(001) < γ(110), the expected ordering for FCC Au, since the close-packed (111) face has the fewest undercoordinated surface atoms and the open (110) face the most.

Table 5 compares these values with the modified embedded-atom method^41^ and with two full-potential electronic-structure treatments: the full charge density linear muffin-tin orbital method (FCD-LMTO)^42^ and the full-potential screened Korringa-Kohn-Rostoker method (FP-KKR).^43^ The ReaxFF/ACKS2 values are higher than all three computational reference values and higher than the experimental estimate of 1.5 J/m^2^ obtained by extrapolation from liquid-metal surface tension measurements.^44^ The differences between facets, however, are reproduced closely: Δγ(001–111) = 0.21 J/m^2^ and Δγ(110–111) = 0.32 J/m^2^ against 0.23 and 0.36 J/m^2^ from FP-KKR. Since it is the relative surface energies that govern the facet dependence of wettability discussed in Section 3.2.2, this agreement is the more relevant test. Surface energies were not training targets for either the Au parameters or the Au-water cross terms optimized here, so these values are predictions of the model.

**Table 5:** Clean-Surface Energies of the Low-Index Au Facets (J/m^2^) Compared with the Modified Embedded-Atom Method, Two Full-Potential Electronic-Structure Methods, and Experiment

Au Surface	This Work	MEAM (2003)^41^	FCD-LMTO^42^	FP-KKR^43^	Expt.^44^
γ(111)	1.68	0.93	1.28	1.39	1.5
γ(001)	1.89	1.14	1.63	1.62	—
γ(110)	2.00	1.18	1.70	1.75	—

#### 3.2.2 Wettability and Adhesion across the Three Facets

Table 6 compiles the CA, work of adhesion, and surface energy for all Au facets, and the ordering has a clear physical reading. All three predicted contact angles fall between roughly 14° and 24°, placing the ReaxFF/ACKS2 description of clean Au firmly on the strongly wetting side of the literature.

**Table 6:** ReaxFF/ACKS2 Predicted Contact Angle, Work of Adhesion, and Clean-Surface Energy for the Three Low-Index Au Facets

Au Facet	Contact Angle (°)	Work of Adhesion (mJ/m^2^)	Surface Energy (mJ/m^2^)
Au(111)	14.0	125.16 ± 9.31	1680
Au(001)	18.1	111.24 ± 8.44	1890
Au(110)	24.1	100.35 ± 7.32	2001

The contact-angle trend is consistent with the work-of-adhesion trend: larger work of adhesion corresponds to smaller contact angles, as expected from the Young–Dupré relation.^15,45^ Au(111) has the largest *W*_*ad*_ and the smallest contact angle, Au(110) has the smallest *W*_*ad*_ and the largest contact angle, and Au(001) sits between them. The surface energy runs in the opposite direction, so that the facet with the lowest clean-surface energy, Au(111), is the most wetting. These seemingly contradictory trends are reconciled by the atomic surface density of the facets. The close-packed Au(111) plane both costs the least energy to create, giving it the lowest surface energy, and packs the most atoms per unit area in contact with the water, giving it the strongest areal adhesion and therefore the lowest contact angle. The open Au(110) plane is the reverse on both counts. A similar dependence of wettability on substrate atomic surface density was noted by Ramos-Alvarado et al.^27^ for the Si(100) and Si(111) surfaces.

#### 3.2.3 Hydrogen-Bonding Network at the Au-Water Interface

To enable a direct comparison with the *ab initio* molecular dynamics results of Velasco-Velez et al.,^46^ a separate Au(111)/water simulation was constructed to replicate their system. The cell contains a six-layer Au(111) slab of 96 atoms with 150 water molecules, with lateral dimensions of 11.81 × 10.23 Å and a total height of 55.0 Å, and is periodic in all three directions with no vacuum region. The water film is therefore confined between the two Au faces of the slab and its periodic image, as shown in Figure 7(a), with an initial water density of 1.00 g cm^–3^ and an initial Au-to-oxygen separation of 2.9 Å at both surfaces. Following energy minimization, the system was equilibrated and propagated in the NVT ensemble at 300 K for 300 ps with a time step of 0.25 fs, and the final 80 ps were used for analysis, matching the trajectory length analyzed in the reference study. The donor populations were stationary well before the start of this window.

**Figure 7:**
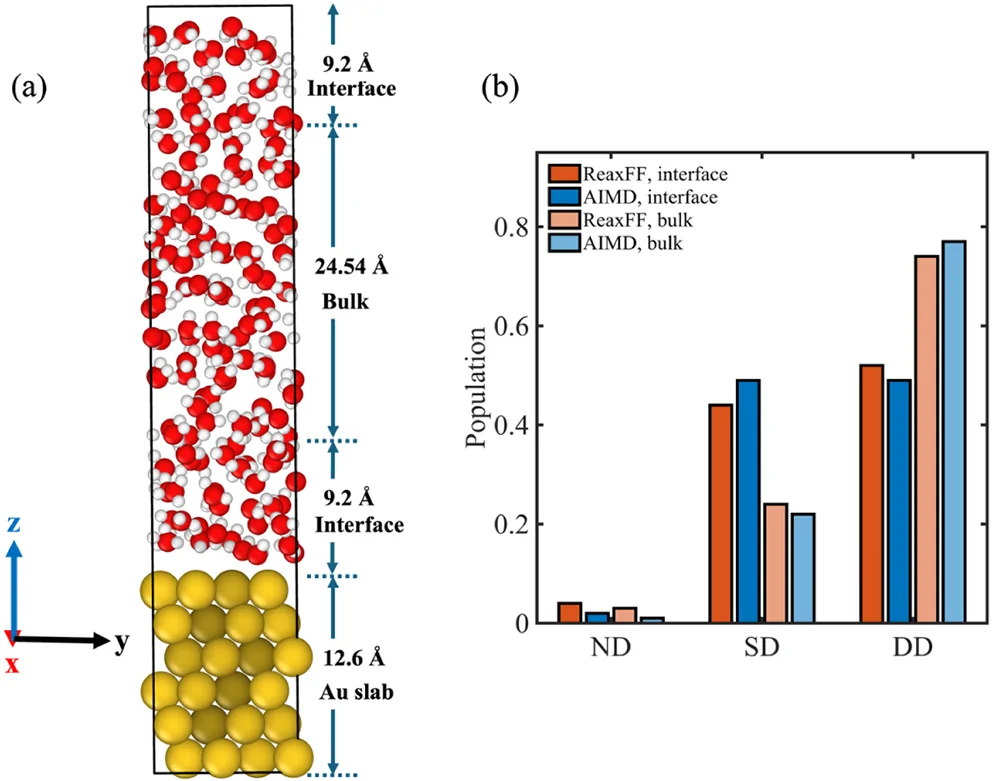
(a) Initial configuration of the Au(111)/water system used for the hydrogen-bond analysis, showing the six-layer Au(111) slab and the 150-molecule water film in a fully periodic cell of 11.81 × 10.23 × 55.0 Å. Gold atoms are shown in gold, oxygen in red, and hydrogen in white. The absence of a vacuum region means that the water film is confined between the two Au surfaces, so both faces of the slab present an equivalent Au-water interface. (b) Hydrogen-bond donor populations for nondonor (ND), single-donor (SD), and double-donor (DD) water molecules in the interfacial region within 9.2 Å of the Au surface and in the bulk region beyond it. ReaxFF/ACKS2 results from this work are compared with the AIMD values of Velasco-Velez et al.,^46^ using identical hydrogen-bond criteria and the same spatial partitioning.

Hydrogen bonds were identified using the geometric criterion of Luzar and Chandler,^47^ with an O···O separation below 3.5 Å and an O–H···O angle of at least 145°, corresponding to the 35° deviation criterion used in the reference work. Each water molecule was classified by the number of hydrogen bonds it donates, as a nondonor (ND), single donor (SD), or double donor (DD), and assigned to a region according to the instantaneous distance of its oxygen atom from the nearer Au surface. Following the partitioning used in the reference study, water within 9.2 Å of a surface was assigned to the interfacial region and the remainder to bulk, which for the present cell corresponds to a bulk band 24.5 Å thick at the center of the film.

Figure 7(b) compares the resulting donor populations with the AIMD values. In the bulk region, the present model yields 3% ND, 24% SD, and 73% DD, against 1%, 22%, and 77% from the AIMD calculations, so the hydrogen-bond topology of the liquid away from the metal is reproduced closely. In the interfacial region, the double-donor population falls to 52%, and the single-donor population rises to 44%, compared with 49% and 49% respectively in the reference work. Expressed as a mean number of donated hydrogen bonds per molecule, the present model gives 1.72 in bulk and 1.48 at the interface, against 1.76 and 1.47 from the AIMD reference. Both descriptions therefore show the same behavior on approach to the surface: roughly one water molecule in four loses a donated hydrogen bond, the single-donor population approximately doubles relative to bulk, and the nondonor population remains small in both regions. The agreement holds for the magnitude of the disruption and not only for its direction. Since no hydrogen-bond statistics, either bulk or interfacial, entered the force field optimization described in Section 2.3, this comparison constitutes an independent test of the interfacial water structure predicted by the model rather than a restatement of the fit.

## 4 Experimental Wettability of Clean Au Surfaces

### 4.1 Cleaning Protocol

Glass slides coated with 100 nm of Au with a 5 nm Ti adhesion layer were purchased from Platypus Technologies and cut into 1 cm × 2 cm strips. The strips were sonicated in deionized water for 10 min; during the sonication, a bath of nanostrip (90% H_2_SO_4_, 5% H_2_SO_5_, ∼4% H_2_O, and ∼1% H_2_O_2_) was preheated to 60 °C. An Au strip was then removed from the DI water and immediately placed into the bath for 5 min; any Au strip not put in the nanostrip remained in the DI water to prevent exposure to atmospheric contaminants. After 5 min, the strip in the bath was removed, rinsed with DI water, and blow-dried with N_2_.

### 4.2 X-ray Diffraction (XRD) Measurements

X-ray diffraction (XRD) measurements were performed using Malvern Panalytical’s Empyrean X-ray diffractometer utilizing Cu Kα radiation (λ = 1.54059 Å), operated at 45 kV and 40 mA. Measurements were collected over a 2θ range of 30°–60° with a step size of 0.02626° and a total acquisition time of 272.34 s.

### 4.3 Contact Angle Measurements

CA measurements were performed utilizing a Ramé-Hart automatic goniometer (290 Series Model) fitted with the Ramé-Hart 4X Macro Imaging Lens, operated under laboratory conditions. Immediately after being dried and placed on the goniometer, a water droplet of 0.5 μL in volume was dispensed on the specimen, and measurements were taken every minute for the first 5 min. After the first 5 min, the time interval between each measurement was expanded. In total, the contact angle measurements were repeated routinely for 4 h (240 min). The collected images were analyzed utilizing the ImageJ software and the DropSnake^48^ tool from the contact angle add-on.

Figure 8 illustrates the time-dependent contact angle measurements of a polycrystalline Au surface composed primarily of the <111> orientation; thermodynamically speaking, this facet has the lowest surface energy (see Table 5). As seen across the literature and the data presented in Figure 8, experimental contact angle measurements of Au vary with the surface conditions of the sample. Without cleaning, Au’s high contact angle is a consequence of adventitious carbon contamination,^49^ a well-documented occurrence in the literature via methods like X-ray photoelectron spectroscopy (XPS).^3,50,51^ Immediately after surface cleaning, a contact angle of 30° was observed, which is consistent with the results of Turetta et al.^8^ We assume this measurement is primarily a result of the hydrophilicity of clean Au. This conclusion is drawn from the fact that cleaning processes like nanostrip create relatively fewer Au oxides^52^ than other methods such as plasma or UV-ozone.^53–55^ The difference between the values of the trained models (14–24°) and the experimental measurements (30°) is explainable when considering model-to-experiment discrepancies in surface conditions/structure and atmospheric contamination.

**Figure 8:**
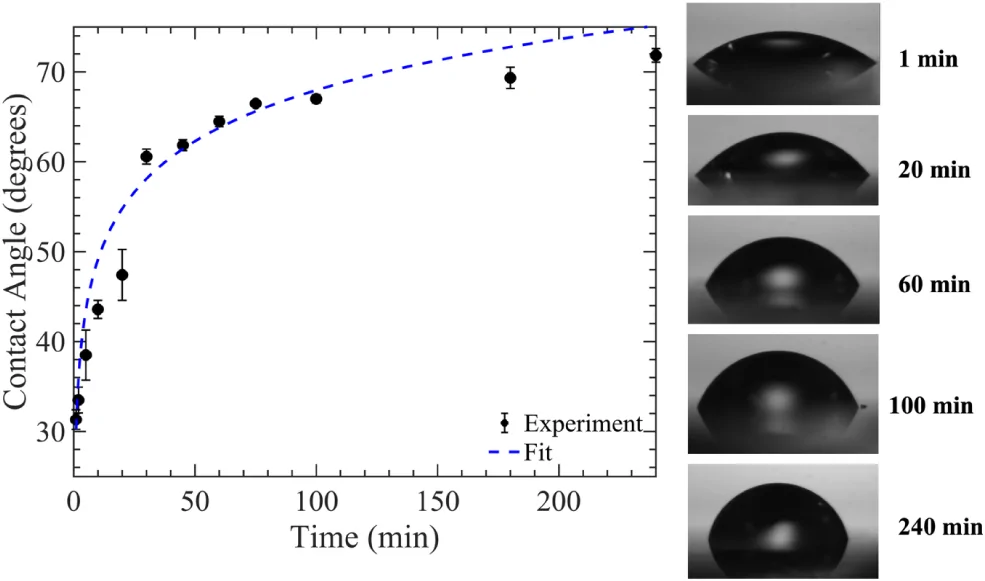
Results of the time-dependent contact angle measurements for the three samples averaged and reported with one standard deviation. The blue dashed line is a basic logarithmic fit. Snapshots of the droplets at major intervals are shown on the right.

Experimentally, our available facilities could not prevent Au sample exposure to the atmosphere, thus negating an exact comparison with the atomically clean models simulated in this study. Furthermore, the purchased gold film was polycrystalline and predominantly terminated by the <111> facet, as confirmed by the singular peak present at 38.2° in the XRD data^56^ in Figure S12 of Section S9 in the Supporting Information. Other surface defects such as scratches, grain boundaries, or nano- and microscale structures (e.g., pillars and steps) can also affect the measured contact angle,^52,57^ while scratches visible to the human eye can be avoided during measurements, smaller defects or structural inhomogeneity cannot.

Additionally, our results demonstrate the slow evolution to higher contact angles that aligns with previous reports.^50^ While our data show that after 4 h, the contact angle is ∼75°, if this process were allowed to continue for several hours, the measurements would have reached 80–90° as the hydrocarbon layer fully covers the surface. Modeling the adventitious carbon contamination effect requires further development of ReaxFF force fields to simulate reaction kinetics of airborne organics with the gold surface, which is beyond the scope of this work.

## 5 Resolving Wettability of Au Surfaces through Simulations and Experiments

When it comes to interfacial liquid properties and solid–liquid affinity, the nonreactive force fields are the least informative. Among the nine nonreactive force fields spanning four functional forms and four parameterization strategies, eight produced complete wetting on all three Au facets at 300 K. Only FF5 retained a small contact angle, yielding CA = 10.96° on Au(001) and CA = 11.20° on Au(110), and complete spreading on Au(111). The near-identical contact angles across two surface orientations are consistent with the surface transferability expected of pair potentials, yet the values themselves, both below 12°, place FF5 firmly in the superhydrophilic regime. This agrees with the earlier use of the same potentials by Paniagua-Guerra and Ramos-Alvarado^29^ and with the DFT-CES result of Gim et al.^33^ that clean noble metals wet completely, but it only tells us that every potential binds water at least as strongly as the spreading threshold.

The ReaxFF/ACKS2 model provides sound chemical/physical insight. The predicted CAs are 14.0° for Au(111), 18.1° for Au(001), and 24.1° for Au(110). By letting water and Au repolarize each other at every step, the reactive model captures an interaction strong enough to drive the surface well into the hydrophilic regime, yet not so strong that the droplet loses its shape. Additionally, the wettability characterization follows the work-of-adhesion ordering from the phantom-wall calculations.

Goniometry on a freshly cleaned surface, performed within 1 min of removal from a nanostrip bath, produced a CA ∼ 30°, which rose to ∼75° over 4 h in ambient air and would likely continue toward 80° to 90° if left longer. The slow, logarithmic rise is the signature of adventitious hydrocarbon adsorption,^5,8^ i.e., bare Au pulls organic molecules from the air, and the aged angle reflects the wetting of that overlayer rather than of the Au beneath it.

It should be stressed that the ∼30° measured on the freshly cleaned film is an effective contact angle and not an intrinsic one. The first measurement is taken roughly 1 min after removal from the nanostrip bath, during which the surface is exposed to laboratory air, and hydrocarbon uptake on clean Au is known to begin immediately. Smith^3^ quantified this directly, correlating the Auger C/Au peak-height ratio with the measured angle and showing that less than one monolayer of adsorbed carbonaceous material is sufficient to raise the contact angle of clean Au from near zero to approximately 50°. On that scale, a residual submonolayer carbon coverage of a few tenths of a monolayer at the time of our first measurement is entirely consistent with an angle near 30°. The measured value should therefore be read as an upper bound on the intrinsic contact angle of clean Au, and the simulated 14.0° to 24.1° on ideal facets as the corresponding clean-surface limit. The residual gap between them reflects the contamination that cannot be eliminated experimentally, together with the polycrystallinity, steps, and roughness of the deposited film. The two numbers bracket the intrinsic value rather than being in conflict. The comparison is therefore between a clean-facet limit and a polycrystalline effective value, not a facet-by-facet validation of the individual simulated angles. The wetting regime was nonetheless reproduced without any wettability-specific tuning of the model, which makes the contact angle a genuine downstream prediction rather than a fitted result.

## 6 Limitations and Outlook

Our ReaxFF/ACKS2 model yields a physically meaningful description of the intrinsic wettability of clean Au, but several limitations remain.

- **The training data.** The Au-water cross terms were fitted against vdW-corrected DFT energies for single water molecules adsorbed on Au, since quantum-mechanical reference calculations for an extended Au slab in contact with a full water film are not computationally tractable at the level of theory required to describe dispersion correctly at this interface. These configurations control the binding strength, orientation, and site preference of the first hydration layer, but they do not sample the collective hydrogen-bonding network or the many-body polarization of an extended interface directly. Water–water interactions are described by an established ACKS2 parameter set that reproduces the structure and dynamics of the bulk liquid, so the cohesive behavior of the droplet away from the metal is not inherited from the single-molecule fit. Improving the description of the contact layer would nonetheless benefit from reference data for extended systems. The natural targets are periodic water bilayers or thin films on Au, small water clusters adsorbed on the surface, and short AIMD trajectories of the Au-water interface, from which forces as well as energies could be included in the objective function. Force matching against such trajectories would constrain the many-body response of the contact layer directly rather than inferring it from monomer energetics, and would be the appropriate route to a more transferable Au-water description.
- **Computational cost.** The bottleneck is the charge-equilibration step rather than the force evaluation. ACKS2 adds a potential variable per atom, doubling the linear system solved at every time step, and the ACKS2 fix is not GPU-enabled in the LAMMPS version used here, so charges and positions are transferred to the host and solved on the CPU at every step. This limited the GPU runs to ∼50 h per nanosecond against ∼80 on CPU alone. The cost is therefore a property of the charge model and its current implementation rather than an inherent limit of reactive MD. A GPU-resident ACKS2 solver would remove the transfer overhead directly, and customized preconditioners^58,59^ or an extended-Lagrangian formulation that propagates the charges and potential fluctuations as dynamical variables^60^ would reduce or avoid the iterative solve itself; both directions are reviewed by Leven et al.^61^ Reducing the charge-update frequency would lower cost proportionally but introduces nonconservative forces, and a hybrid EEM/ACKS2 scheme would help little here, since the region where EEM fails is the interface, which is also the region of interest.
- **Idealized nature of the simulated surfaces.** The contact angles were computed on perfect, atomically flat, single-facet slabs, whereas real Au films are polycrystalline and carry steps, terraces, grain boundaries, roughness, and adsorbed impurities. As discussed in Section 4, these features explain why the measured angle on a (111)-dominated polycrystalline film sits somewhat above even the least wetting facet we simulated, and they make a one-to-one comparison between simulation and experiment difficult. The nanoscale droplets and nanosecond time scales used here add residual finite-size and sampling effects that can shift the computed angles by a few degrees, although they are unlikely to move the values out of the strongly hydrophilic range.
- The liquid–vapor surface tension of the ReaxFF/ACKS2 water description was not computed here, and the equilibrium density lies 1.4% below the experimental value. Since the surface tension enters the Young-Dupré relation as a common factor across all facets, this does not affect the facet ordering or the relative wettability of the three surfaces, but it could contribute a small systematic offset to the absolute contact angles. Quantifying that offset would require computing the surface tension of the model and repeating the wettability calculations across a range of water descriptions, which we identify as a target for future work.

The most direct way forward is to move from single-facet slabs to polycrystalline Au models containing grains, grain boundaries, steps, and roughness, so the facet-resolved predictions can be assembled into an effective contact angle for a realistic surface and compared with experiment on a common footing. A second priority is to simulate airborne hydrocarbon contamination explicitly, tracking how the contact angle climbs with organic coverage. Since both this work and the experiments attribute the high angles on aged Au to adsorbed hydrocarbons, reproducing the logarithmic time dependence of Section 4 (Figure 8) from such a model would be a strong test of that interpretation.

The framework also opens broader directions. The same force field can be extended to Au nanoparticles in water, where curvature, size, and the distribution of exposed facets may alter the wetting behavior, which is directly relevant to solvated nanoparticles used in plasmonic heating and biomedical delivery. It can be used to study how ions, pH, and surface defects modify the Au-water contact angle. And because wettability and interfacial adhesion govern the vibrational coupling that carries heat across a solid–liquid boundary, the model provides a foundation for computing the thermal boundary conductance at Au-water interfaces, linking contact angle, work of adhesion, interfacial water structure, and heat transport in aqueous Au systems.

## 7 Conclusions

We developed and tested a ReaxFF/ACKS2 reactive force field for Au-water wettability and combined it with controlled contact-angle experiments to close the knowledge gap between computations and experiments on clean Au surfaces. The simulations predicted CAs of 14.0° for Au(111), 18.1° for Au(001), and 24.1° for Au(110), all well inside the hydrophilic regime, showing that clean Au wets strongly without spreading completely. Our work of adhesion calculations, performed using the phantom-wall method of Leroy and Müller-Plathe, followed the same ordering, with the close-packed Au(111) face binding water most strongly and the open Au(110) face most weakly, and the clean-surface energies ran in the opposite order, linking wettability, adhesion, and surface energy through the atomic surface density of the facets. The model was validated on both components, reproducing the structure, dynamics, and diffusion of bulk water against the SPC/E and TIP3P models, and capturing the cohesive energy of FCC Au to within ∼2% along with the sign and rough magnitude of its thermal expansion.

Goniometry conducted on freshly cleaned polycrystalline Au yielded a contact angle near 30° that rose to 75° over 4 h in ambient air, consistent with progressive hydrocarbon adsorption. Once contamination, surface defects, and finite-size effects are accounted for, the predicted 14° to 24° on the ideal facets and the measured 30° on a (111)-dominated film bracket the intrinsic value of clean Au from below and above. The two end points of the literature range are therefore not in conflict: complete wetting reflects an upper bound on the Au-water interaction, the 80° to 90° values reflect the wetting of an adsorbed hydrocarbon layer, and clean Au sits at a consistent intermediate value. The ReaxFF/ACKS2 model thus provides a physically grounded description of clean Au-water wettability and a foundation for future studies of polycrystalline and contaminated surfaces, nanoparticle wetting, and interfacial heat transfer in aqueous Au systems.

## Supplementary Material



## Notes

Addendum Sections: S1 – Nonreactive force-field parameters description. S2 – Comparison of the ReaxFF water description against nonreactive models and experimental data. S3 – Validation of the ReaxFF Au description against experimental data. S4 – Nonreactive droplet simulation snapshots. S5 – Nonreactive Au-water interaction energy. S6 – Contact angle calculations for FF5. S7 – Contact angle calculations using the ReaxFF/ACKS2 parameter set. S8 – Work of adhesion and surface energy calculations. S9 – X-ray diffraction characterization of the Au film. S10 – Complete optimized ReaxFF/ACKS2 force field parameter file for the Au–H–O system (PDF)
