# Supplementary material for: Unraveling the Wettability of Gold Surfaces: A Reactive Molecular Dynamics and Experimental Approach
Source: J Phys Chem C Nanomater Interfaces. 2026 Sep 28;130(40):14007–21. doi: 10.1021/acs.jpcc.6c04449 (PMC13660127; doi:10.1021/acs.jpcc.6c04449)
Supplement: Supplementary file 1 [file jp6c04449_si_001.pdf]

Supporting Information for

Unraveling the Wettability of Gold Surfaces: A Reactive Molecular Dynamics and Experimental Approach

Emdadul Haque Chowdhury,[1] Md Shahed Hossain Sohan,[1] Evan Buckwalter,[2] Seong H. Kim,[2] Adri C.T. van Duin,[1] and Bladimir Ramos-Alvarado[1,2, a)]

[1]Department of Mechanical Engineering, The Pennsylvania State University, University Park, PA16802, USA.

[2]Department of Chemical Engineering, The Pennsylvania State University, University Park, PA16802, USA.

a) Authors to whom correspondence should be addressed: bzr52@psu.edu

Section S1. Non-reactive force-field parameters description

S1.1. LJ 12-6 potentials with Au-O interactions (FF1 to FF4)

FF1 through FF4 use the 12-6 LJ potential and restrict Au-water interactions to the Au-O pair, ignoring Au-H terms:

$$V(r) = 4\varepsilon[\left(\frac{\sigma}{r}\right)^{12} - \left(\frac{\sigma}{r}\right)^{6}] \quad \text{(S1)}$$

where ε is the well depth, and σ is the collision diameter (zero-crossing distance). FF1 (Merabia et al. [1]) is the only potential in the set calibrated directly to wettability; The Au-O well depth (ε = 2.4686 kJ/mol) was derived by substituting a target contact angle of 25° into the Young equation, using the SPC/E liquid-vapor surface tension at 300 K and an analytical estimate of the LJ contributions to the surface tensions. In practice, when applied to flat crystallographic surfaces via the cylindrical droplet method, FF1 produces complete spreading on all three facets, as do the other eight force fields. FF2 (Tay and Bresme[2]) was parameterized against experimental water adsorption energies on Au, yielding the largest well depth in the set (ε = 8.28 kJ/mol). It was

originally applied to passivated nanoparticles rather than flat surfaces, and when transferred to flat Au interfaces, it generates the highest interfacial interaction energies of the nine force fields. FF3 (Heinz et al. [3]) derives Au-water cross terms by applying the Lorentz-Berthelot combining rules to optimized Au-Au LJ parameters; this approach is internally consistent but known to perform poorly at metal-fluid interfaces.[4,5] FF4 (Gim et al.[5]) is the only potential in this group fit to a vdW-DF2 DFT binding energy curve that directly addresses wettability, which predicts complete wetting (CA = 0°) for clean noble-metal surfaces.

S1.2. Multi-site potentials with Au-O and Au-H interactions (FF5 to FF9)

FF5 through FF9 include explicit interactions for both water atoms, allowing the potential to respond to the orientation of the water molecule at the surface. FF5 through FF8 were developed together by Berg et al.[4] against vdW-DFT adsorption energies for water on Au (111), using the 12-6 LJ (FF5), 9-6 LJ (FF6), Buckingham (FF7), and Morse (FF8) functional forms. Berg et al. showed that the steep r^{-12} repulsive wall of the standard 12-6 LJ form (FF5) distorts both the well depth and its position relative to the DFT reference, while Buckingham and Morse potentials reproduce the short-range repulsion more accurately. None of the four were tested for contact angle. FF9 (Dou et al.[6]) was developed for simulations of laser-induced explosive boiling of water on Au, using a Morse potential for Au-O and a Born-Mayer-Huggins corrugation term for Au-H. The corrugation term differentiates on-top, bridge, and hollow adsorption sites on the Au lattice, making FF9 the only site-sensitive potential in this set; it was not applied to contact angle simulations previously.

Section S2. Comparison of the ReaxFF water description against non-reactive models and experimental data

Before applying the trained force field to the Au-water interface, we verified that the ReaxFF/ACKS2 description reproduces the dynamical and structural behavior of bulk liquid water. Five properties were computed: the velocity autocorrelation function (VACF), the vibrational density of states (vDOS), the mean square displacement (MSD), the self-diffusion coefficient (D), and the radial distribution function (RDF). Each was compared against the widely used non-reactive water models SPC/E and TIP3P, against the polarizable AMOEBA[7] model for the vDOS, and against experimental data for the diffusion coefficient. The goal was to confirm that making water reactive and letting its charges respond through ACKS2 does not degrade the bulk-water behavior relative to the established fixed-charge models.

The bulk-water simulations used a cubic box of 1024 water molecules with an edge length of 31.33 Å. This box generates a density of ~1.0 g/cm^3 at 300 K, with periodic boundary conditions in all three directions. The system was first relaxed using the conjugate-gradient energy minimization algorithm to remove close contacts, then equilibrated for 500 ps in the NVT ensemble at 300 K using a Nosé-Hoover thermostat with a 100 fs damping constant, a velocity-Verlet integrator, and a 0.25 fs time step. The production stage was a 500 ps run in the NVE ensemble. During production, oxygen-atom velocities were saved every 2.5 fs for the velocity-based analyses and atomic positions every 50 fs for the displacement-based analyses. The oxygen velocity was used as a proxy for the molecular center-of-mass velocity, which is reasonable because the oxygen atom carries about 89% of the molecular mass. For the diffusion coefficient, the same protocol was repeated at several temperatures.

The VACF, MSD, D, and RDF were computed following the procedures of Lee [8], and the vDOS following Khusnutdinoff and Mokshin.[9] Figure S1(a) depicts the normalized VACF. All

three models start at unity, decay within ~100 fs, pass through a small positive shoulder near 130 fs, and reach a shallow negative minimum between 200 and 300 fs before relaxing to zero by roughly 500 fs. The negative region is the signature of cage rattling, where a water molecule reverses direction after colliding with the wall of its hydrogen-bonded cage. The ReaxFF curve follows SPC/E closely, including the depth of the negative minimum, which shows that the reactive model captures the short-time librational and translational dynamics of liquid water. Figure S1(b) illustrates the corresponding vDOS, the spectral form of the VACF. Liquid water has two intermolecular bands: a low-frequency band near 10 ps^{-1} (~50 cm^{-1}) assigned to hydrogen-bond bending and the hindered O-O-O cage motion, and a higher band near 45 ps^{-1} (~240 cm^{-1}) in the hydrogen-bond stretching region. The rigid fixed-charge models SPC/E and TIP3P produce a tall, sharp low-frequency peak, while ReaxFF gives a lower and broader first band with a clearer stretching band than either rigid model resolves. The polarizable AMOEBA model gives a still broader, lower-amplitude distribution. The broader ReaxFF band reflects a wider spread of local hydrogen-bond environments and a more anharmonic network than the rigid fixed-charge models impose. The low-frequency band sits near the experimental bending frequency at 50 to 60 cm^{-1},[10] and the stretching band falls above the experimental value near 175 to 200 cm^{-1},[11] so ReaxFF reproduces the bending mode accurately while placing the stretching mode somewhat high.

Figure S1(c) details the MSD, which is linear in the diffusive regime for all three models. The slopes order as TIP3P > SPC/E > ReaxFF, with TIP3P reaching ~32 Å^2 at 10 ps, SPC/E ~16 Å^2, and ReaxFF ~13 Å^2. Figure S1(d) presents the self-diffusion coefficients extracted from the MSD slopes, plotted as log D against 1000/T. The lower mobility of ReaxFF seen in the MSD carries directly into this plot. All four series are straight on the Arrhenius axes, so both the reactive model and the reference data follow the thermally activated form of water diffusion. ReaxFF lies

below SPC/E across the range, and both fall below the two experimental series at the higher temperatures, while the four curves draw together near 300 K. Near 300 K, the ReaxFF diffusion coefficient is somewhat lower than the experimental value of ~2.3×10^{-9} m^2/s, so the reactive model is mildly under-diffusive in bulk water. The slope of the ReaxFF Arrhenius line, which sets the activation energy for diffusion, matches the experiment well, so the temperature dependence is captured, while the absolute value is underpredicted. Note that the SPC/E, Exp1, and Exp2 data are taken from Lee.[8] The two experimental series are independent determinations of the water self-diffusion coefficient, obtained by pulsed-field-gradient ^1H NMR and by isotopic tracer (diaphragm-cell) measurements, which agree closely and bracket a value near 2.3×10^{-9} m^2/s at 298 K.

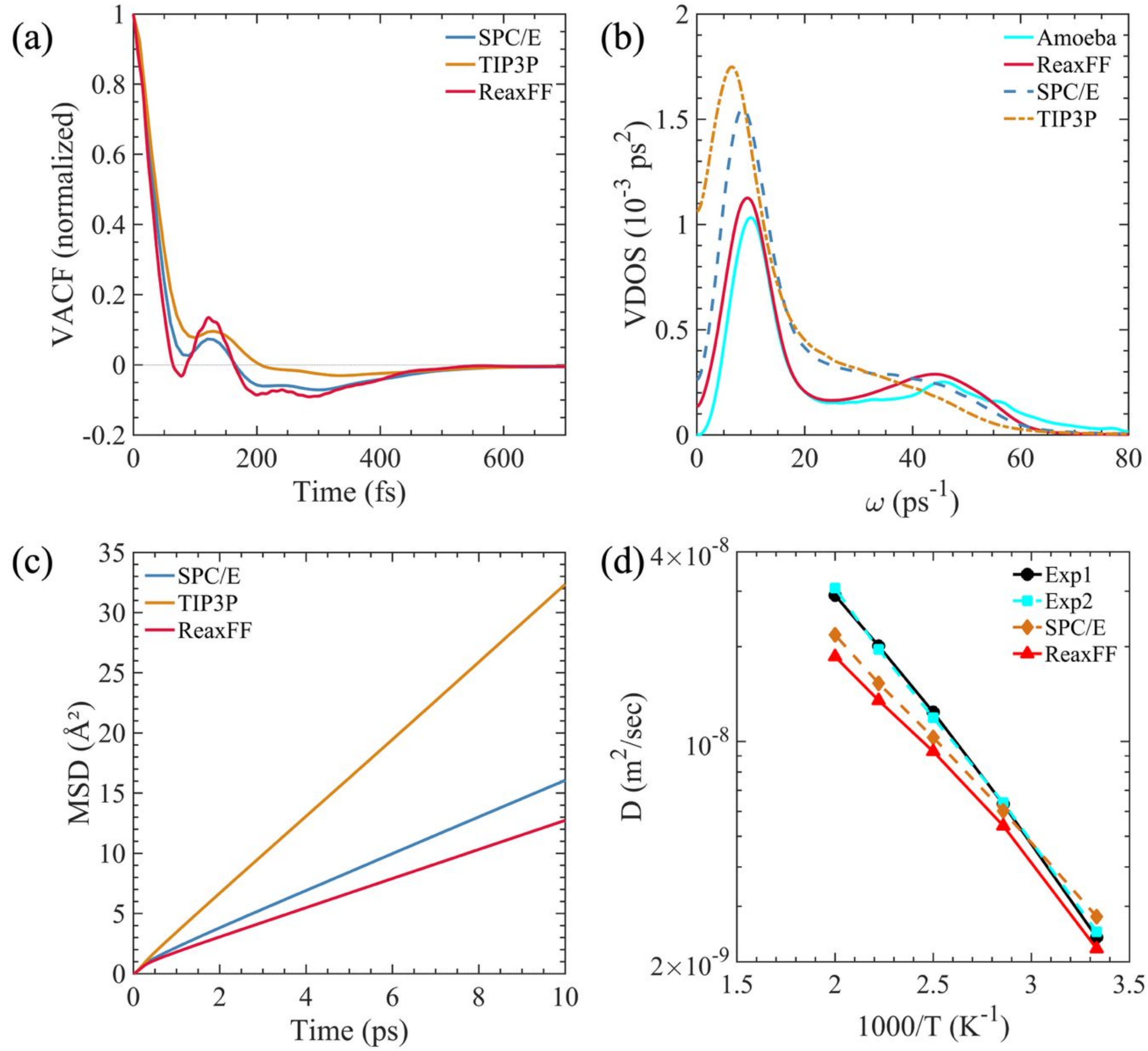

Figure S1. Bulk liquid-water validation of the ReaxFF/ACKS2 model. (a) Velocity autocorrelation function from ReaxFF, SPC/E, and TIP3P at 300 K. (b) Vibrational density of states from ReaxFF, SPC/E, TIP3P, and AMOEBA at 300 K. (c) Mean square displacement of the oxygen atoms from ReaxFF, SPC/E, and TIP3P at 300 K. (d) Self-diffusion coefficient on an Arrhenius plot from ReaxFF and SPC/E, compared with two independent experimental datasets (Exp1 and Exp2).

Figure S2 compares the radial distribution functions of the three models for the (a) O-O, (b) O-H, and (c) H-H atom pairs. For g_{OO} the first peak sits near 2.8 Å in all three models, matching the experimental nearest-neighbor oxygen distance,[12] and the first minimum near 3.3 Å is also reproduced. ReaxFF gives a first-peak position and height close to SPC/E and TIP3P, and its second and third shells near 4.2 and 6.5 Å are more sharply defined than the broader, flatter features of the two non-reactive models, which points to somewhat stronger second-neighbor ordering in the reactive description. The g_{OH} functions show the hydrogen-bonded peak near 1.8 Å and the main peak near 3.2 Å, with ReaxFF tracking the non-reactive models closely through the first two peaks. The g_{HH} functions show their first peak near 2.4 Å and a second feature near 3.8 Å, again with ReaxFF in line with SPC/E and TIP3P. The agreement across all three pair correlations confirms that the reactive description reproduces the local structure of liquid water, not only its dynamics.

Figure S2(d) adds an independent thermodynamic check by comparing the equilibrium density of ReaxFF water with NIST[13] reference data over a range of pressures. The same 1024-molecule box used for the dynamical analyses was first energy-minimized, then run in the NPT ensemble at 300 K for 3 ns at each pressure between 0.1 and 1.5 MPa, with the equilibrium density taken as the average over the final 1 ns. ReaxFF predicts a density of ~0.983 g/cm^3, roughly 1.4% below the NIST value of ~0.997 g/cm^3, and both data sets are essentially flat across this pressure range, as expected for a nearly incompressible liquid at these low pressures. The small underestimate of the bulk density is a minor and acceptable deviation for a reactive model that was not specifically fitted to the liquid density, and it does not affect the structural and dynamical agreement established above.

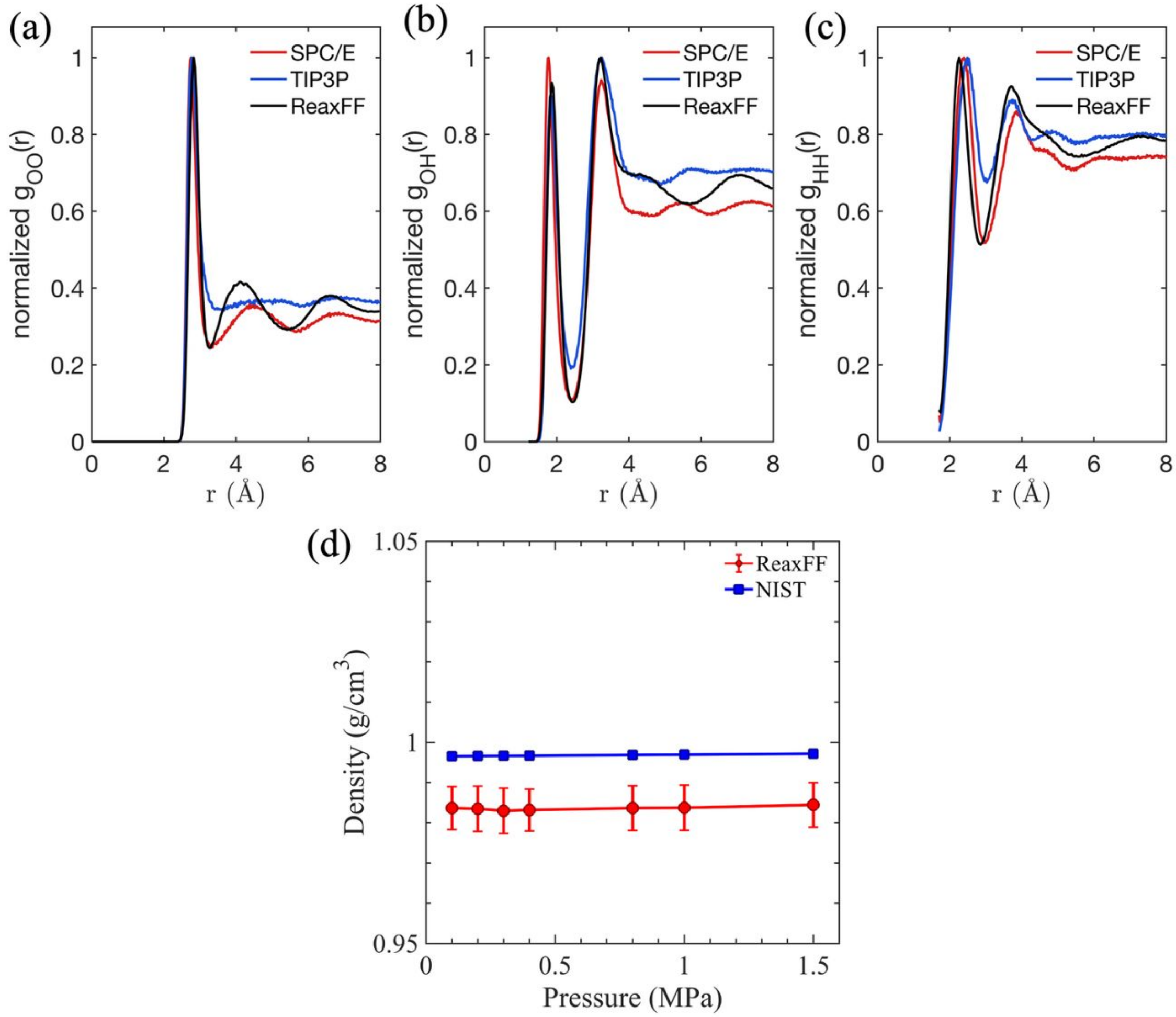

Figure S2. Radial distribution functions from ReaxFF, SPC/E, and TIP3P for the (a) O-O, (b) O-H, and (c) H-H pairs. (d) Equilibrium density of ReaxFF water as a function of pressure at 300 K from NPT simulations, compared with NIST reference data.

Taken together, these comparisons place the ReaxFF/ACKS2 water model on par with established water models in both structure and dynamics. SPC/E calculations from Lee[8] served as the primary non-reactive benchmark for the structural and dynamical tests, with ReaxFF closely reproducing its radial distribution functions and short-time velocity correlations, while AMOEBA provided the polarizable reference for the vibrational density of states. ReaxFF differs from these

models in that the water molecule is fully flexible, and the partial charges respond to the local environment through ACKS2 rather than being held fixed. The cost of this generality is a modest under-prediction of the bulk diffusion coefficient relative to experiment, but the structural correlations, the vibrational band positions, and the activation energy for diffusion are all captured, and the equilibrium density stays within about 1.4% of the NIST reference. Because the reactive, charge-responsive description is exactly what is needed to capture charge transfer and polarization at the Au-water interface, and because it does not sacrifice the bulk-water behavior, the model is well suited to the wettability simulations that follow.

Section S3. Validation of the ReaxFF Au description against experimental data

The Au side of the force field was validated by computing the temperature-dependent lattice constant and unit-cell volume of FCC Au and comparing them against the experimental measurements of Pamato et al.[14] They determined the thermal expansion of Au by X-ray powder diffraction from 40 K up to the melting point at 1337 K, using a heated sample stage with MgO as an internal standard and refining the Au lattice parameter from the diffraction pattern recorded at each temperature. They fitted the measured unit-cell volumes to a second-order Grüneisen approximation to the zero-pressure equation of state and found that the expansion becomes nonlinear above about 800 K, which they attributed to the thermal generation of point defects ahead of melting. Our comparison covers 200 to 700 K, which lies below that defect-dominated regime, so the experimental values here represent the regular thermal expansion of the Au lattice.

Figure S3 depicts the experimental and calculated lattice constant “a” in panel (a) and the unit-cell volume “V” in panel (b). ReaxFF captures the temperature effect in both parameters. Nonetheless, the calculated values are offset across the whole temperature range, i.e., the lattice

constant is ~2.4% higher in panel (a) and the unit-cell volume is ~7.4% higher in panel (b). These two offsets are consistent with V = a^3, since for a small fractional change, the volume offset is close to three times the linear offset ($1.024^3 = 1.074$, or 7.4%). The experimental data rise more steeply with temperature than the ReaxFF prediction; thus, the model underestimates the thermal expansion coefficient, and the relative offset narrows slightly toward the high-temperature end.

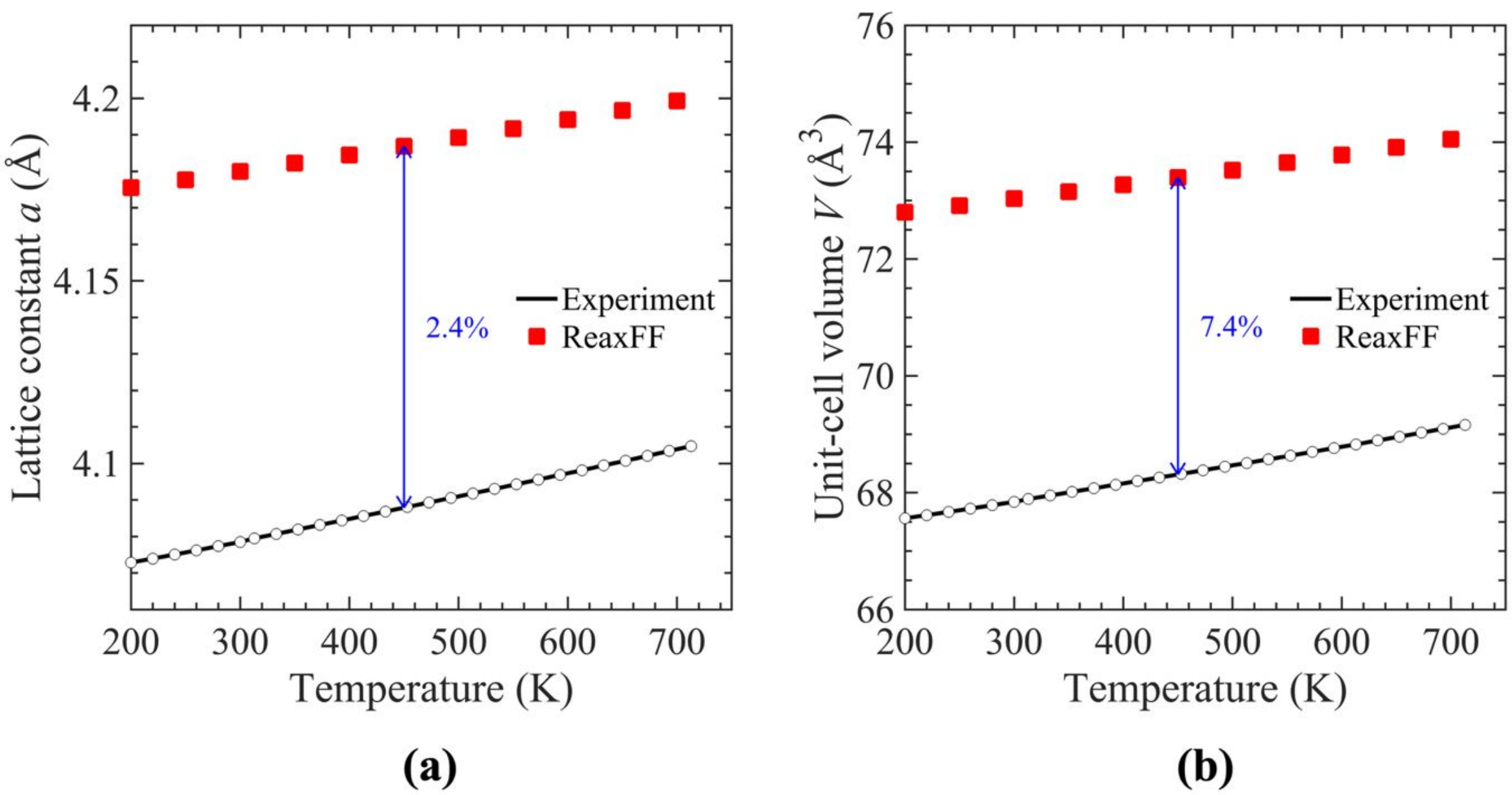

Figure S3. Comparison between experimentally obtained and ReaxFF predicted (a) lattice constant "a" and (b) the unit-cell volume "V". ReaxFF predicted lattice constant is ~2.4% higher in panel (a) and the unit-cell volume is ~7.4% higher in panel (b).

The roughly 2.4% overestimate of the Au lattice constant originates from the Keith et al. parameter set,[15] which was fit to PBE-GGA DFT data for bulk and nanoparticle Au. The same bias is already present in that reference, where Keith et al.[15] reported an FCC lattice constant of 4.16 Å against the experimental 4.08 Å and note that PBE-GGA and ReaxFF both overestimate Au lattice constants relative to measurements. Figure S3 confirms that the model captures the correct sign

and magnitude of the thermal expansion, and this small offset does not affect the computed contact angles, which are dominated by the Au-water interaction and surface coordination rather than a small deviation in Au's lattice constant.

Additionally, we computed the cohesive energy of FCC Au, a fundamental measure of how strongly a crystal is bound. Cohesive energy is the energy required to separate the solid into isolated atoms. It reflects the depth of metallic bonding and correlates with properties such as melting temperature, surface energy, and vacancy formation energy. It is a standard and demanding benchmark for an interatomic potential, since a force field can only describe surfaces, defects, and adhesion reliably if it first reproduces the bulk binding energy. To compute it, we built a $3 \times 3 \times 3$ conventional FCC supercell of Au containing 108 atoms in a cubic box of 12.4959 Å, corresponding to the ReaxFF equilibrium lattice constant of 4.16528 Å, and relaxed it using conjugate-gradient energy minimization. The cohesive energy was taken as the difference between the energy of an isolated Au atom and the per-atom potential energy of the relaxed bulk crystal. The ReaxFF/ACKS2 cohesive energy is 3.75 eV/atom. This is within 2% of the experimental cohesive energy of Au, which is 3.81 eV/atom in the compilation of Kittel [16] and 3.82 eV/atom in the dedicated re-evaluation by Arblaster [17]. The value also lies within the range of recent first-principles predictions for bulk Au, such as van der Waals-corrected DFT calculations that give cohesive energies between roughly 3.5 and 3.7 eV/atom across the best-performing methods [18], and CCSD(T)-F12 cluster calculations extrapolated to the bulk give about 3.87 eV/atom [19]. The model therefore slightly underbinds the Au crystal by less than 0.1 eV/atom relative to experiments, which is good agreement for a reactive force field and shows that the Keith parameterization captures the strength of metallic Au-Au bonding, not only the lattice geometry and its thermal expansion.

Section S4. Non-reactive droplet simulation snapshots

Figure S4 shows representative simulation snapshots taken at the end of the 4 ns NVT equilibration and 2 ns NVE production runs (6 ns total) for all nine non-reactive force fields as discussed in Section S1 (and Section 2.2 of the main manuscript), on Au facets (110 and 111). These frames directly reveal the wetting state of each system: a retained cylindrical droplet indicates partial wetting with a finite contact angle, while a water film covering the surface indicates complete wetting with the contact angle approaching zero.

Eight of the nine non-reactive force fields produce complete wetting on all three surface orientations, with the initial cylindrical droplet spreading into a uniform film during the NVT equilibration period. FF5 is the only exception, retaining a finite droplet on Au(001) and Au(110) at the end of the 6 ns simulation. To confirm this behavior was not a kinetic artifact of insufficient simulation time, the NVT run for the FF5/Au(001) and FF5/Au(110) cases was extended to 8 ns; the droplet persisted on the Au(110) surface (Figures S4.i(e), S4.i(f)). On Au(111), FF5 produces complete wetting (Figure S4.ii(e)). For the remaining eight force fields, the wetting state is consistent across all three surfaces at 6 ns, indicating that force field parameterization is a primary determinant of wetting behavior in this set. The relative strength of the Au-water interaction compared with water-water cohesion is shown quantitatively in Section S5.

(i) Au(110)

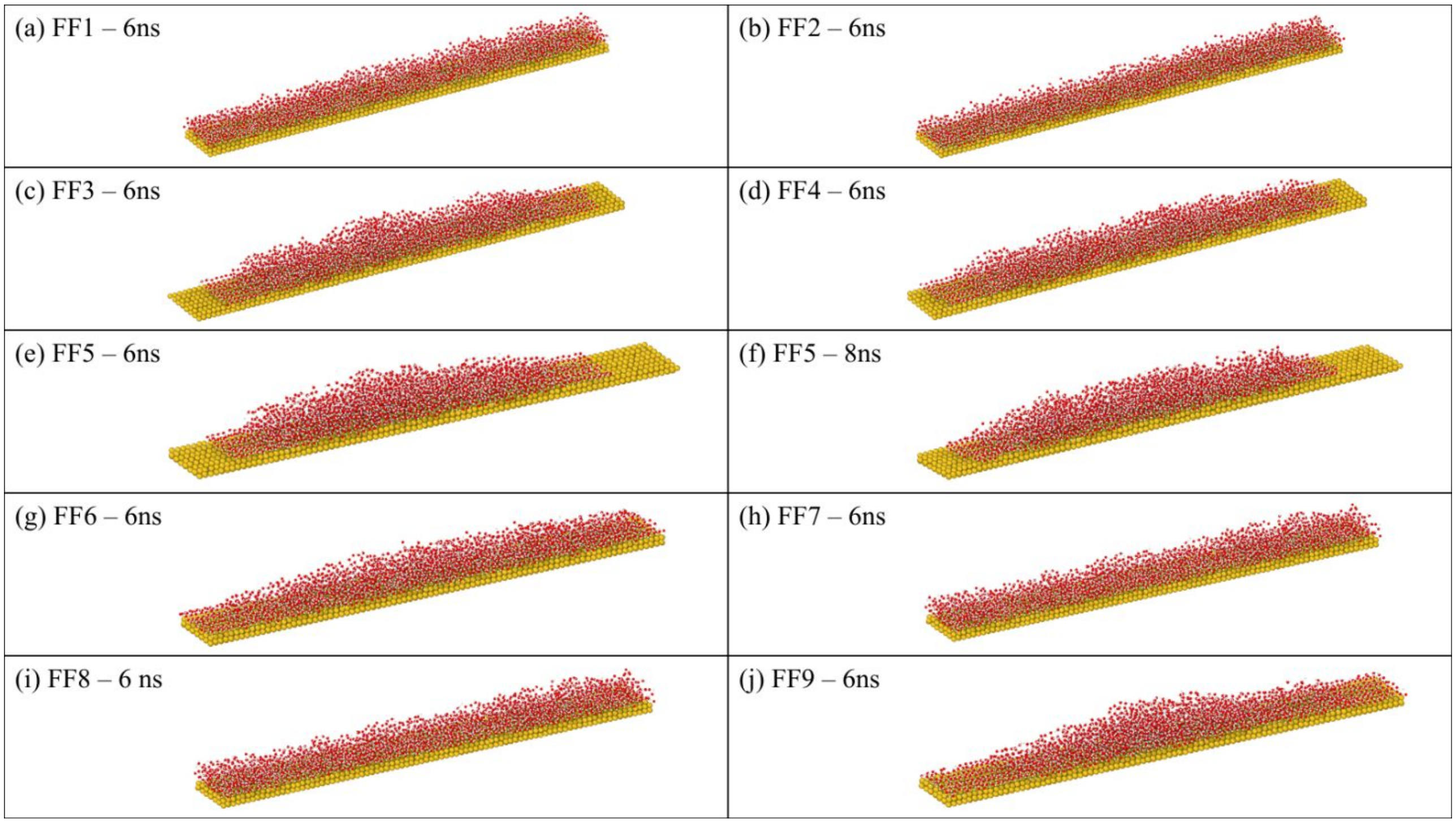
(a) FF1 – 6ns
(b) FF2 – 6ns
(c) FF3 – 6ns
(d) FF4 – 6ns
(e) FF5 – 6ns
(f) FF5 – 8ns
(g) FF6 – 6ns
(h) FF7 – 6ns
(i) FF8 – 6 ns
(j) FF9 – 6ns

(ii) Au(111)

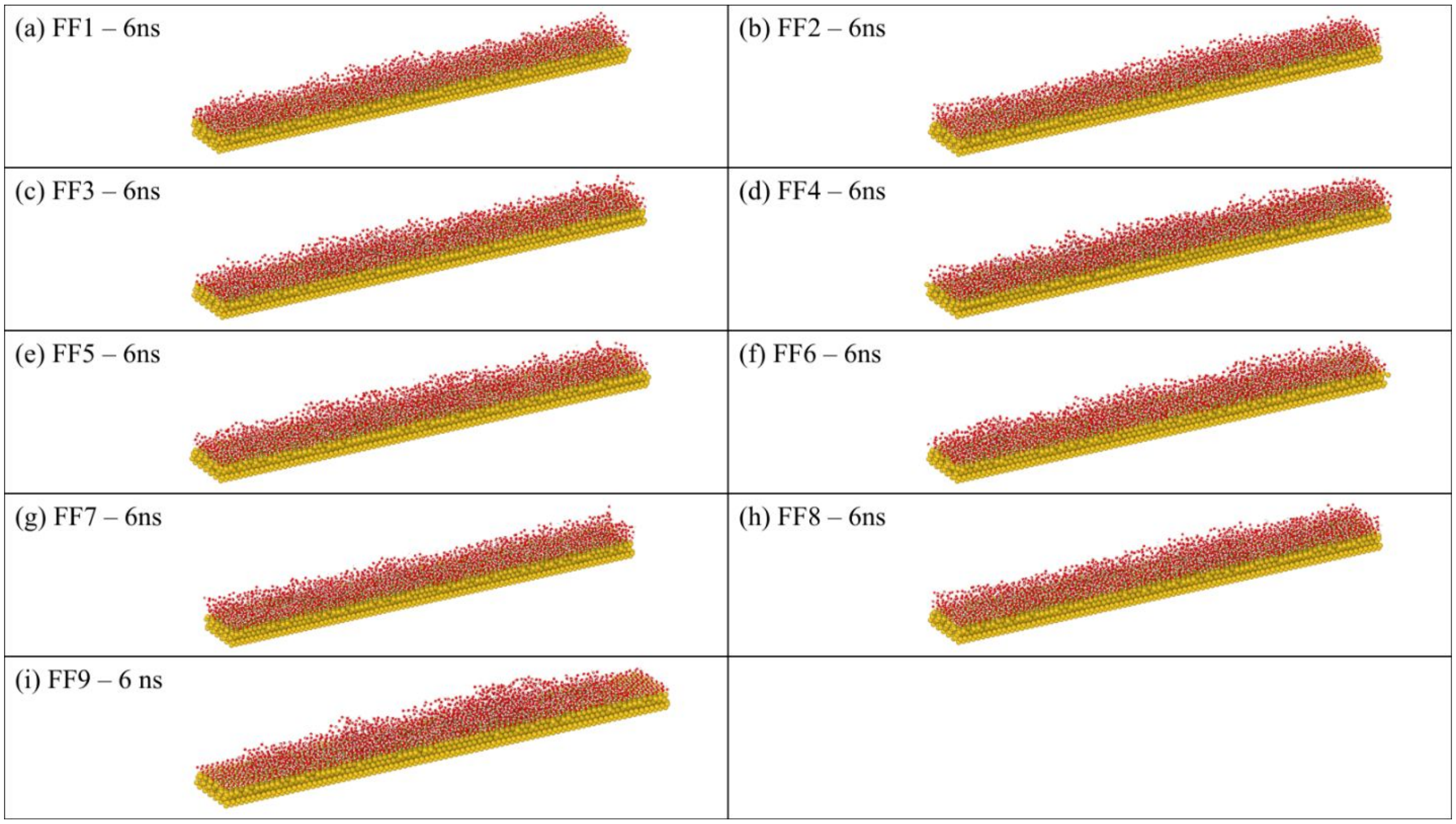

Figure S4. Representative (t = 6 ns) simulation snapshots for all nine non-reactive force fields on the Au(110), and Au(111) surfaces. Au atoms are shown in yellow, oxygen in red, and hydrogen in white. A flat water film indicates complete wetting (θ ~ 0°); a retained cylindrical geometry indicates partial wetting with a finite contact angle.

Section S5. Non-reactive Au-water interaction energy

To understand why only FF5 retains a finite contact angle, the time-averaged Au-water interaction energy (IE) per unit surface area was computed for all nine force fields. The IE is the total pairwise potential energy between the Au slab and water groups, computed using the LAMMPS compute group/group command and normalized by the surface cross-sectional area $A = L_x \times L_y$. Values are reported in kJ mol^{-1} nm^{-2}, following the convention of Paniagua-Guerra and Ramos-Alvarado [20], and represent the mean over the last 80% of the 2 ns NVE run, with the standard deviation reported as the uncertainty. Table S1 details that FF5 has the weakest Au-water

interaction energy of all nine force fields on every surface, at −1663.5 ± 10.9 kJ mol^{-1} nm^{-2} on Au(001), −1532.8 ± 11.9 kJ mol^{-1} nm^{-2} on Au(110), and −2125.2 ± 12.1 kJ mol^{-1} nm^{-2} on Au(111). This weak adhesion explains the unique wetting behavior of FF5 reported in Section S4. Au(111) shows the most negative IE across all nine force fields, followed by Au(001), then Au(110). This ordering reflects the higher surface atom packing density of the close-packed (111) face, which provides more Au-water contacts per unit area than the more open (001) and (110) faces. This ordering is summarized in Figure S5.

Table S1. Time-averaged Au-water interaction energy [kJ mol^{-1} nm^{-2}] for all nine non-reactive force fields on three Au surface orientations. Values are mean ± standard deviation over the last 80% of the 2 ns NVE production run. More negative values indicate stronger Au-water attraction.

Force field	Functional form	Au(001)	Au(110)	Au(111)
FF1 (Merabia et al.)	LJ 12-6	−2468.8 ± 13.1	−2284.7 ± 11.5	−2750.8 ± 12.1
FF2 (Tay and Bresme)	LJ 12-6	−6265.8 ± 14.2	−6489.8 ± 18.6	−7312.1 ± 16.4
FF3 (Heinz et al.)	LJ 12-6	−2344.8 ± 49.0	−2120.1 ± 21.8	−2513.6 ± 13.1
FF4 (Gim et al.)	LJ 12-6	−2471.5 ± 43.7	−2243.7 ± 12.0	−2506.8 ± 13.3
FF5 (Berg et al., Fit 1)	LJ 12-6	−1663.5 ± 10.9	−1532.8 ± 11.9	−2125.2 ± 12.1
FF6 (Berg et al., Fit 2)	LJ 9-6	−2364.4 ± 33.5	−2195.5 ± 29.8	−2586.1 ± 12.1
FF7 (Berg et al., Buckingham)	Buckingham	−2958.2 ± 12.4	−2950.2 ± 15.4	−3408.6 ± 13.6
FF8 (Berg et al., Morse)	Morse	−2990.8 ± 12.4	−2931.5 ± 12.2	−3507.7 ± 13.0
FF9 (Dou et al.)	Morse+BMH	−4998.8 ± 13.6	−4981.0 ± 25.0	−5870.3 ± 24.0

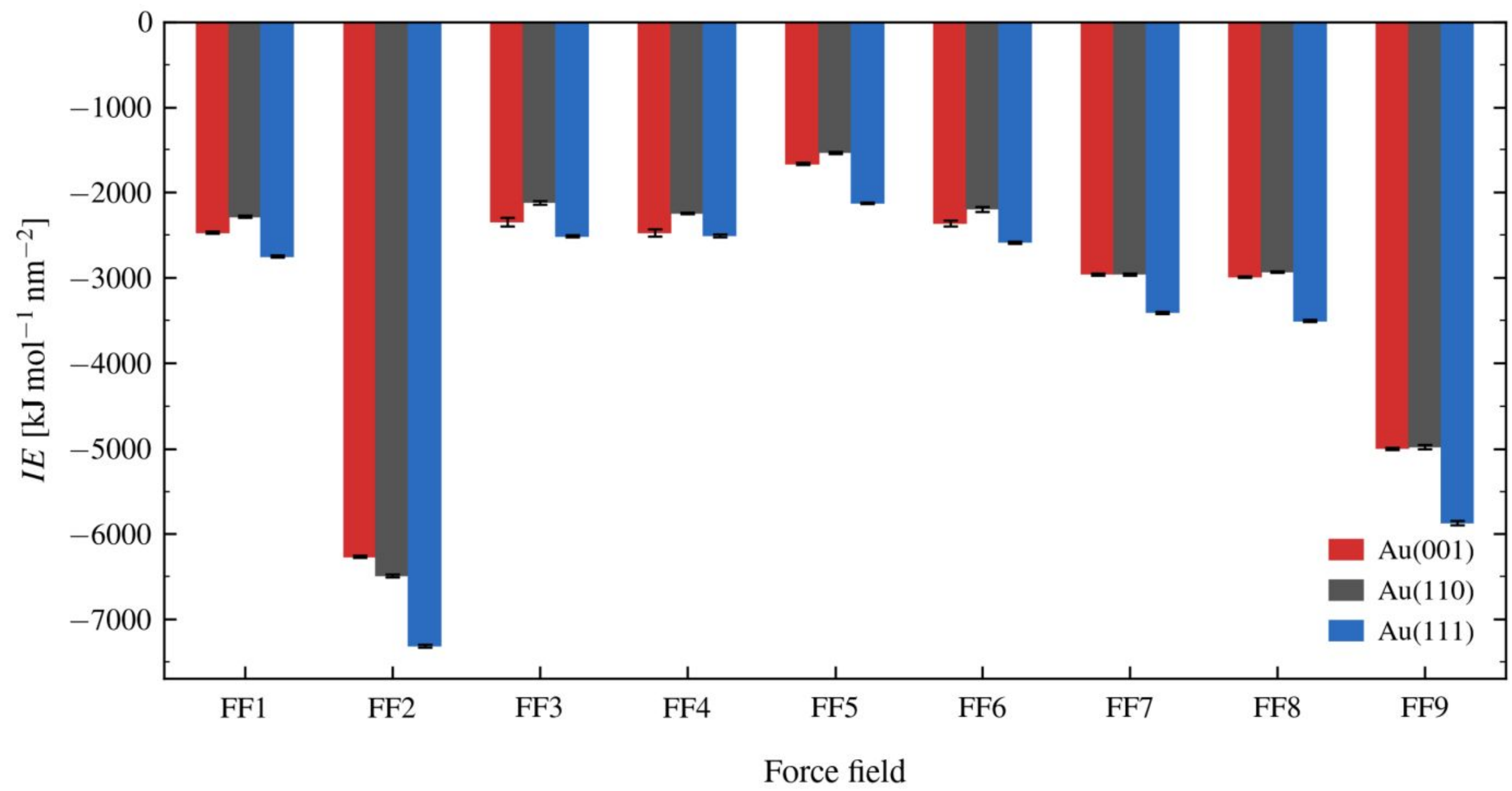

Figure S5. Time-averaged Au-water interaction energy per unit area for force fields FF1–FF9 on Au(001) (red), Au(110) (black), and Au(111) (blue) surfaces. Error bars denote one standard deviation. FF5 exhibits the weakest Au-water attraction among the nine parameterizations, which is the mechanistic origin of its unique ability to retain a finite contact angle.

Section S6. Contact angle calculations for FF5

FF5 is the only non-reactive force field that retains a finite droplet on Au(001) and Au(110) (Section S4), so quantitative contact angle calculations were performed for these two cases using the multi-response postprocessing method of Ramos-Alvarado et al.[21] as described in Section 2.2 of the main manuscript. The weakest Au-water interaction energy of all nine non-reactive force fields (Table S1, Section S5), FF5, generates a contact angle of ~11° for Au(001) and Au(110), placing it within the hydrophilic regime; the fitting parameters are listed in Table S2.

Figure S6 and S7 provides corresponding density fields and circular fits for FF5 on Au(001) and Au(110), respectively. The water density profiles (Figure S8) provide a structural

complement to this result. All nine force fields differ in the degree of hydration-layer ordering, and the differences track the Au–O interaction parameters in Table 3, i.e., force fields with deeper, narrower potential wells (e.g., FF2, parameterized to the experimental Au–water adsorption energy) produce a taller, more confined first hydration layer than weaker-binding force fields such as FF5, consistent with stronger Au–O attraction drawing water into a tighter contact layer. Together, the contact-angle, interaction-energy, and density-profile results indicate that contact angle, Au–water interaction energy, and contact-layer structure are complementary descriptors of force field behavior at this interface, rather than contact angle alone being a sufficient metric of force field fidelity.

Table S2. Contact angle fitting parameters for FF5 on Au(001) and Au(110).

Surface	Fitted radius R (Å)	Base radius (Å)	RMSE circular (Å)	R^2	CA (°)
Au(001)	426.29	81.02	0.397	0.965	10.96
Au(110)	427.07	82.99	0.199	0.960	11.20

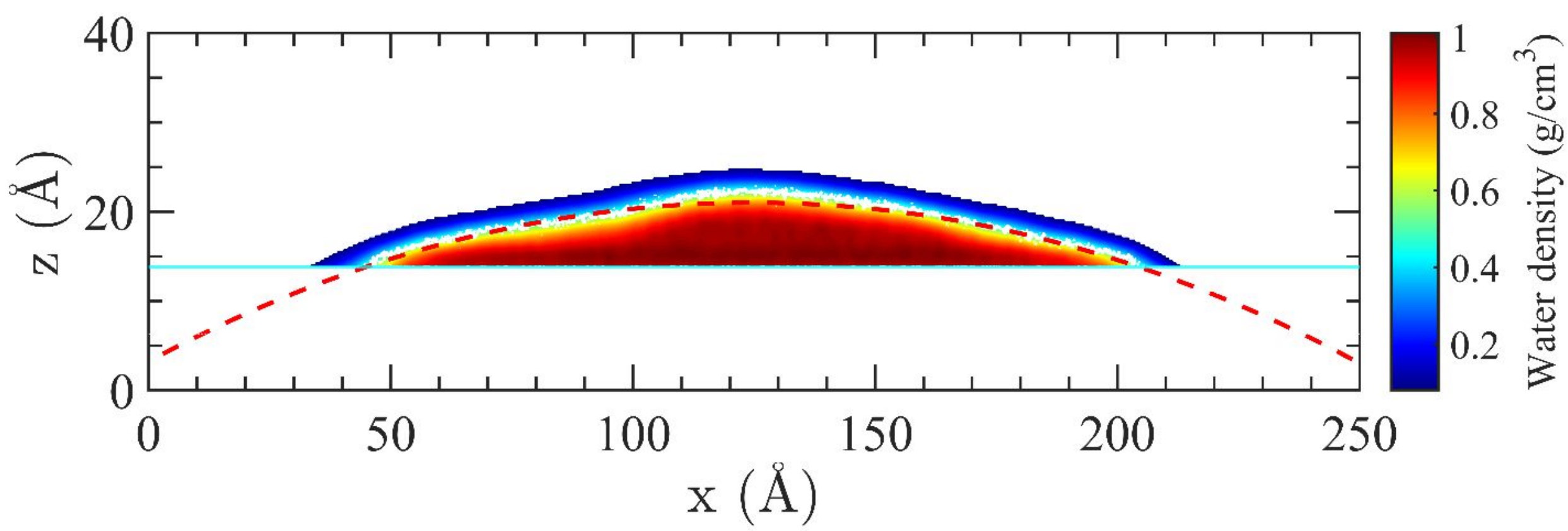

Figure S6. Time-averaged two-dimensional water density field and circular arc fit (dashed line) for FF5 on Au(001). CA = 10.96°.

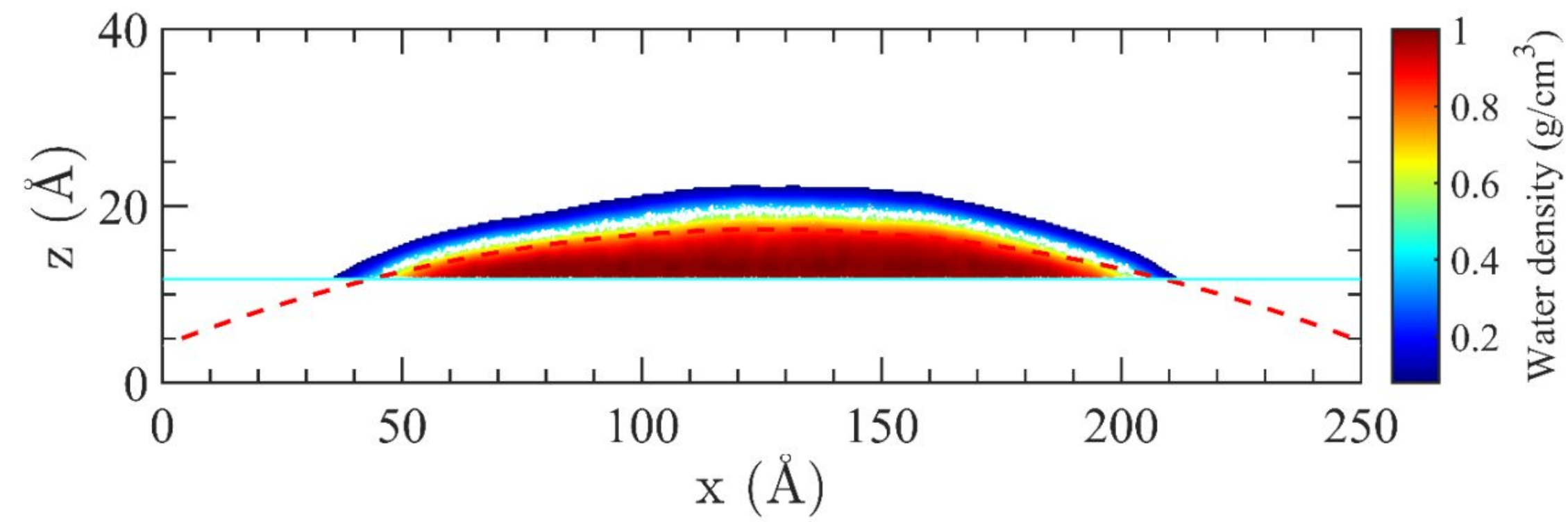

Figure S7. Time-averaged two-dimensional water density field and circular arc fit for FF5 on Au(110). CA = 11.20°.

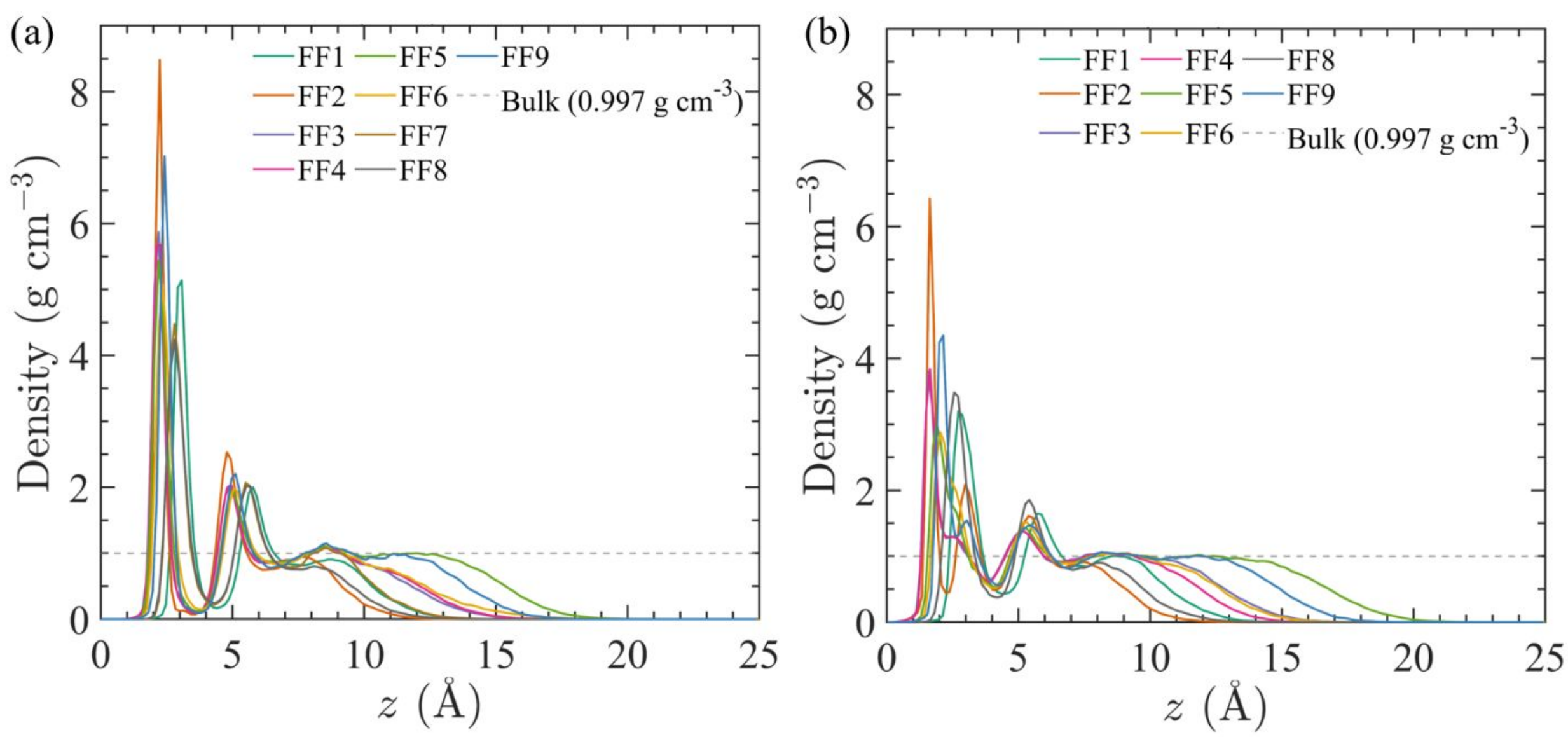

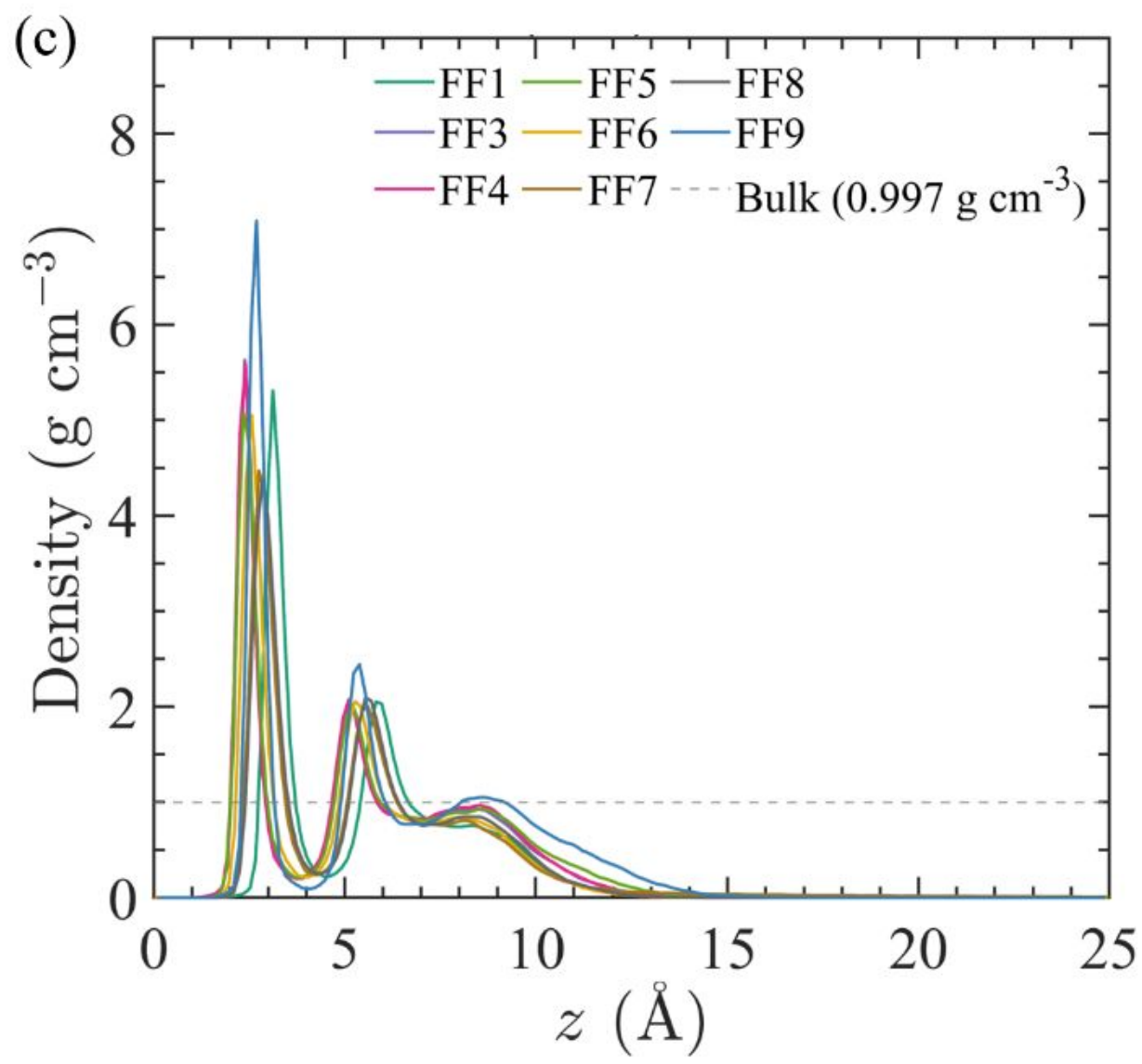

Figure S8. Time-averaged water density profiles ρ(z) along the surface normal for the (a) Au(001)/water, (b) Au(110)/water, and (c) Au(111)/water interfaces, computed for all nine non-reactive Au–water force fields (FF1–FF9).

Section S7. Contact angle calculations using the ReaxFF/ACKS2 parameter set

The contact angle was extracted from the equilibrated NVT trajectories of water using the multi-response post-processing method of Ramos-Alvarado et al.[21] The simulation cell was divided into a uniform grid in the x-z plane, with the full y-extent collapsed into the bin depth, and the time-averaged mass density ρ(x, z) was accumulated over all frames in the analysis window. A bin resolution of 0.25 × 0.25 Å was used, fine enough to resolve the layered structure near the surface while keeping the bulk region smooth. The bulk liquid density and the position of the liquid-vapor interface were obtained by fitting the centerline density profile, the cut of ρ(x, z) through the middle of the droplet, with the sigmoidal form of de Ruijter et al.:[22]

$$\rho(z) = \frac{1}{2}(\rho_L + \rho_v) - \frac{1}{2}(\rho_L - \rho_v)\tanh\left[\frac{z - z_e}{d_e/2}\right] \quad \text{(S2)}$$

where ρ_L and ρ_v are the bulk liquid and vapor densities, z_e is the position of the equimolar surface, and d_e measures the interface thickness. The vapor density was set to zero, and the fit was restricted to the region above the first interfacial peak to avoid biasing the bulk value with interfacial layered water. The liquid-vapor interface was then located as the contour where the field falls to half the fitted bulk density, $\rho_{L/2}$, with linear interpolation between bins so that the interface points were not locked to the grid. The strongly layered region within ~5 Å of the Au was discarded because there the local density oscillates well above and below the bulk value (i.e., it is the entropically affected interface region), so the half-density contour does not define a liquid-vapor interface and the profile departs from the circular form assumed by the Young analysis. The sensitivity of the extracted angle to this cutoff is documented in Table S3. The remaining interface points were fitted to a circle by least squares. The macroscopic contact angle is the angle between the tangent to that circle at its intersection with the Au surface and the surface itself, calculated through the liquid. The contact angle calculation diagnostics are reported in Figures S9 and S10 for the Au(110) and Au(111) facets, respectively

Table S3. Contact angle (°) extracted for each Au facet as a function of the exclusion distance applied above the Au surface before the circular fit.

**Exclusion (Å)**	**Au(001)**	**Au(110)**	**Au(111)**
4.0	19.74°	25.13°	16.20°
4.5	18.47°	24.40°	15.60°
5.0	18.10°	24.10°	14.02°
6.0	17.80°	23.70°	13.90°
7.5	17.54°	23.10°	13.70°

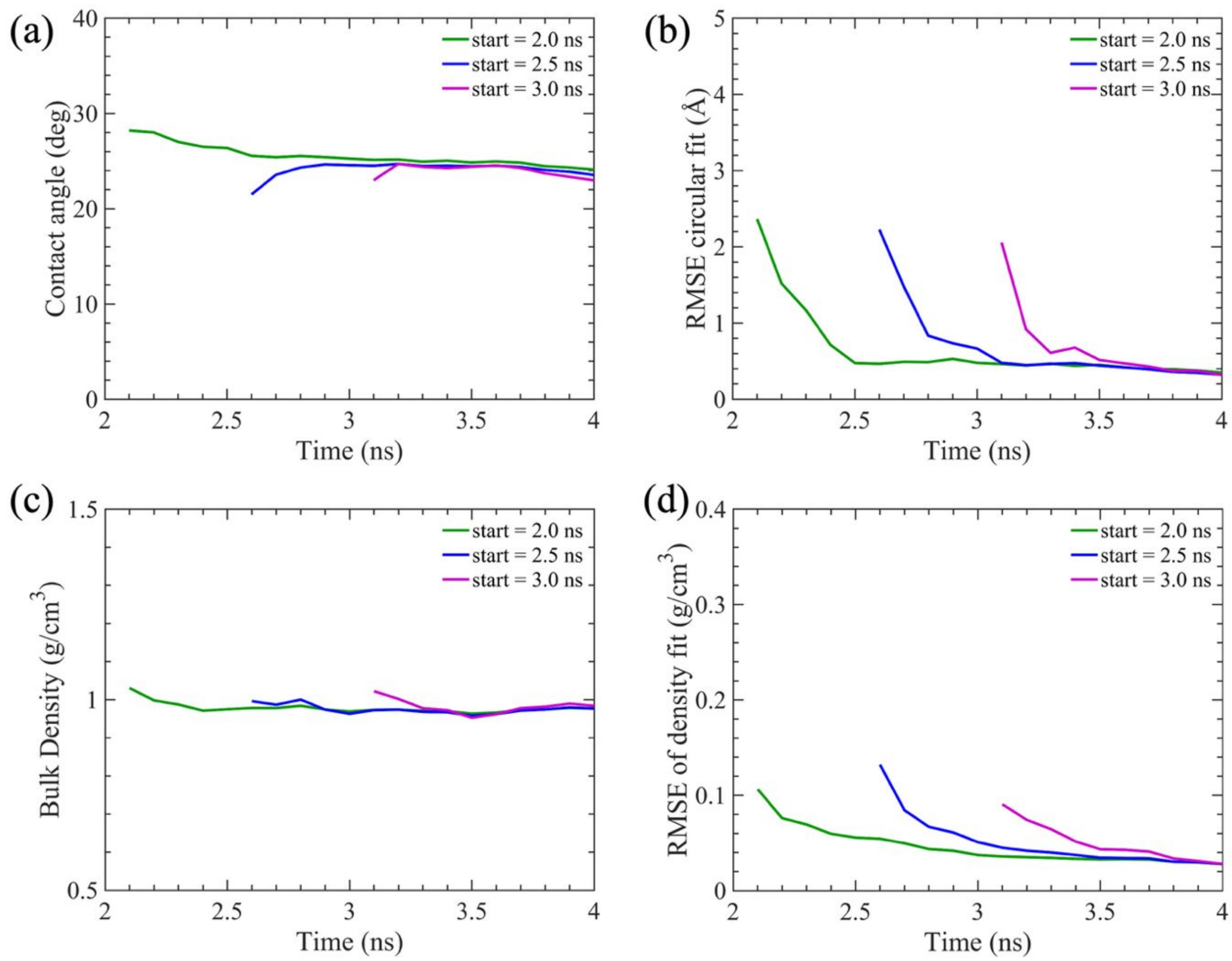

Figure S9. Convergence diagnostics of the contact-angle analysis for the Au(110) system, shown as cumulative averages from five start times (t_{start} = 2.0 to 3.0 ns). (a) Cumulative contact angle, (b) root-mean-square error (RMSE) of the circular fit, (c) cumulative centerline bulk density, and (d) RMSE of the sigmoidal density-profile fit.

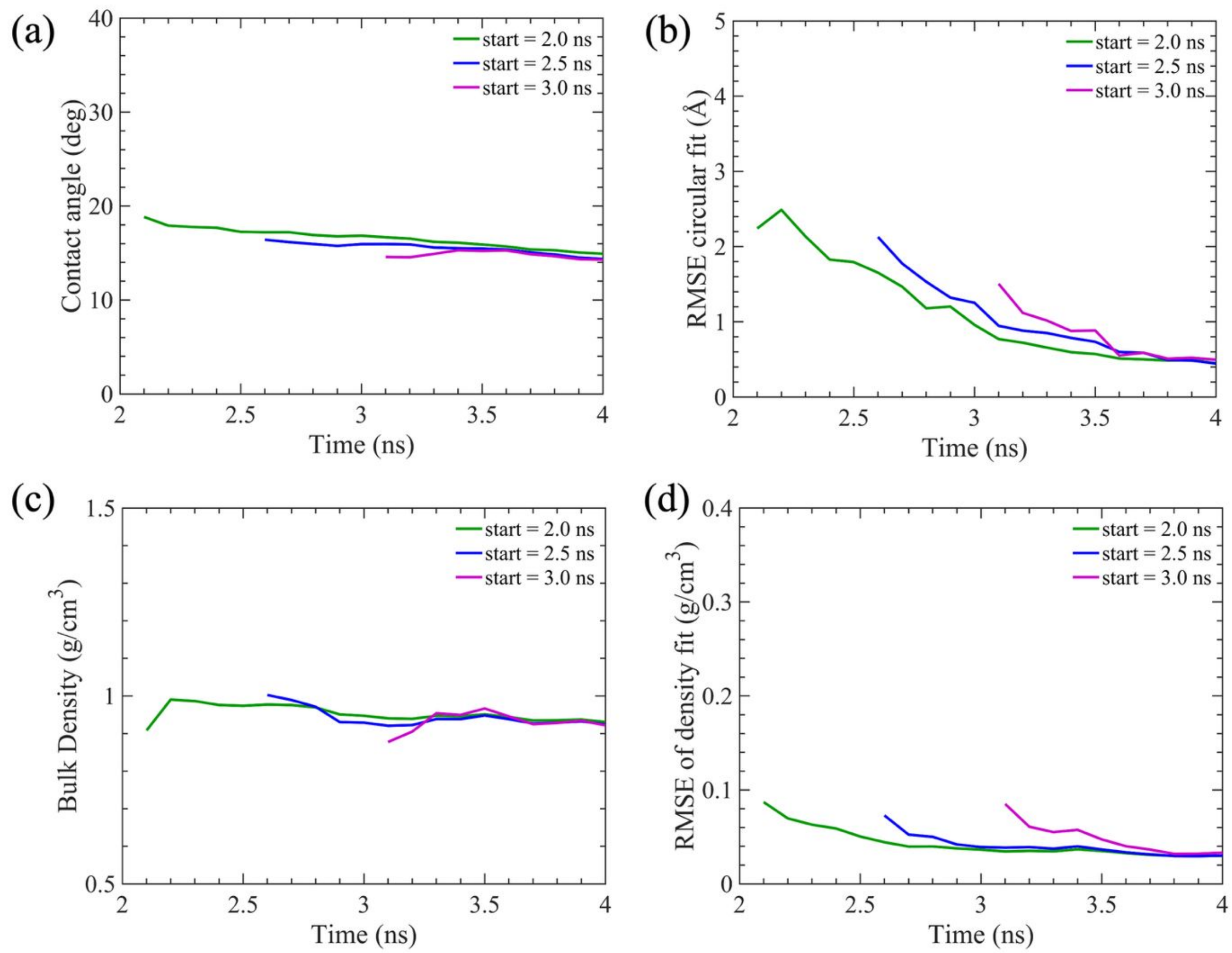

Figure S10. Convergence diagnostics of the contact-angle analysis for the Au(111) system, shown as cumulative averages from five start times (t_{start} = 2.0 to 3.0 ns). (a) Cumulative contact angle, (b) root-mean-square error (RMSE) of the circular fit, (c) cumulative centerline bulk density, and (d) RMSE of the sigmoidal density-profile fit.

291

292 **Section S8. Work of adhesion and surface energy calculations**

293 The work of adhesion W_{ad} between water and each Au surface was computed with the
294 phantom-wall method of Leroy and Müller-Plathe.[23] These calculations used a separate set of flat
295 slab-on-slab systems, one per facet, rather than the cylindrical droplets used for the contact angle.

For each facet, a water film was placed on the Au surface with an initial gap of ~2.65 Å between the topmost Au atom and the lowest water oxygen; the simulation box featured a 200 Å vacuum spacing along the *z*-direction, and the two bottommost Au layers were held fixed to anchor the slab. Each system was relaxed by conjugate-gradient minimization and then equilibrated in the NVT ensemble at 300 K for 12.5 ps using a Nosé-Hoover thermostat with a 25 fs damping constant applied separately to the mobile Au atoms and to the water. All interactions used the ReaxFF/ACKS2 force field with a 10 Å cutoff.

The cross-sectional (*x*–*y*) dimensions of each system are listed in Table S4, and each of the three Au slabs was four atomic layers thick. The *z*-dimension of the simulation box was set to 200 Å to provide sufficient vacuum space above the water slab.

Table S4. Cross-sectional dimensions and composition of the flat slab-on-slab systems used for the phantom-wall work-of-adhesion calculations. Each Au slab is four atomic layers thick, and the box *z*-dimension was 200 Å.

Surface	Lx (Å)	Ly (Å)	Number of Au atoms	Number of water molecules
Au(001)	20.40	20.40	200	139
Au(110)	14.43	20.40	100	118
Au(111)	11.54	19.98	128	123

The phantom wall itself is a mathematical repulsive plane with no mass or charge, implemented with the fix wall/lj126 command in LAMMPS[24] and applied only to the water oxygen atoms, so the Au is unaffected. The wall-oxygen interaction is purely repulsive and follows the Weeks-Chandler-Andersen form; that is, the Lennard-Jones 12-6 potential truncated and shifted at its minimum:

$$E(r) = 4\varepsilon\left[\left(\frac{\sigma}{r}\right)^{12} - \left(\frac{\sigma}{r}\right)^{6}\right] + \varepsilon; r < r_c \quad \text{(S3)}$$

$$E(r) = 0; r > r_c \quad \text{(S4)}$$

where r is the distance from an oxygen atom to the wall plane and the cutoff $r_c = 2^{(1/6)}\sigma$ sits at the LJ minimum, so both the energy and its derivative vanish at the cutoff. The wall parameters were set to the SPC/E oxygen-oxygen values, ε = 0.1554 kcal/mol and σ = 3.166 Å, which give r_c = 3.554 Å. Parameterizing the wall as an oxygen-oxygen interaction provides a physically reasonable repulsive barrier without introducing any artificial attraction between the wall and the liquid. The wall was placed at the start of the traversal ~2.4 Å below the topmost Au atom, roughly 5.1 Å below the lowest water oxygen, far enough that its 3.554 Å interaction range did not yet reach the water, and the force on the system was zero at the first step.

The wall was then advanced upward along z at a constant quasi-static velocity of 0.001 Å/fs, which corresponds to 0.00025 Å per 0.25 fs timestep. As it moved into range, it pushed up on the lowest oxygen atoms, peeling away first the contact layer and then the entire water film from the Au slab. The traversal continued until the oxygen nearest the surface had been lifted at least 10 Å above the top Au layer, beyond the 10 Å ReaxFF cutoff, so that the Au-water interaction

energy had fully decayed. The total z-force exerted by the wall on the water was recorded at every step, and the work of adhesion was obtained from

$$W_{ad} = \left(\frac{1}{A}\right)\int F_{wall}(z)dZ - \frac{P_N \Delta V}{A} \quad \text{(S5)}$$

where A is the interfacial cross-sectional area, P_N is the time-averaged normal pressure, and ΔV is the change in box volume. The force integral was evaluated by the trapezoidal rule over the wall displacement, the pressure-volume correction was negligible for all three facets (below 0.01 mJ/m²), and the raw integral was converted to interfacial units using 1 kcal mol^{-1} $Å^{-2}$ = 694.77 mJ/m². Figure S11 illustrates the method for Au(001), showing the initial Au-water configuration and the state after the wall has pushed the water column clear of the slab.

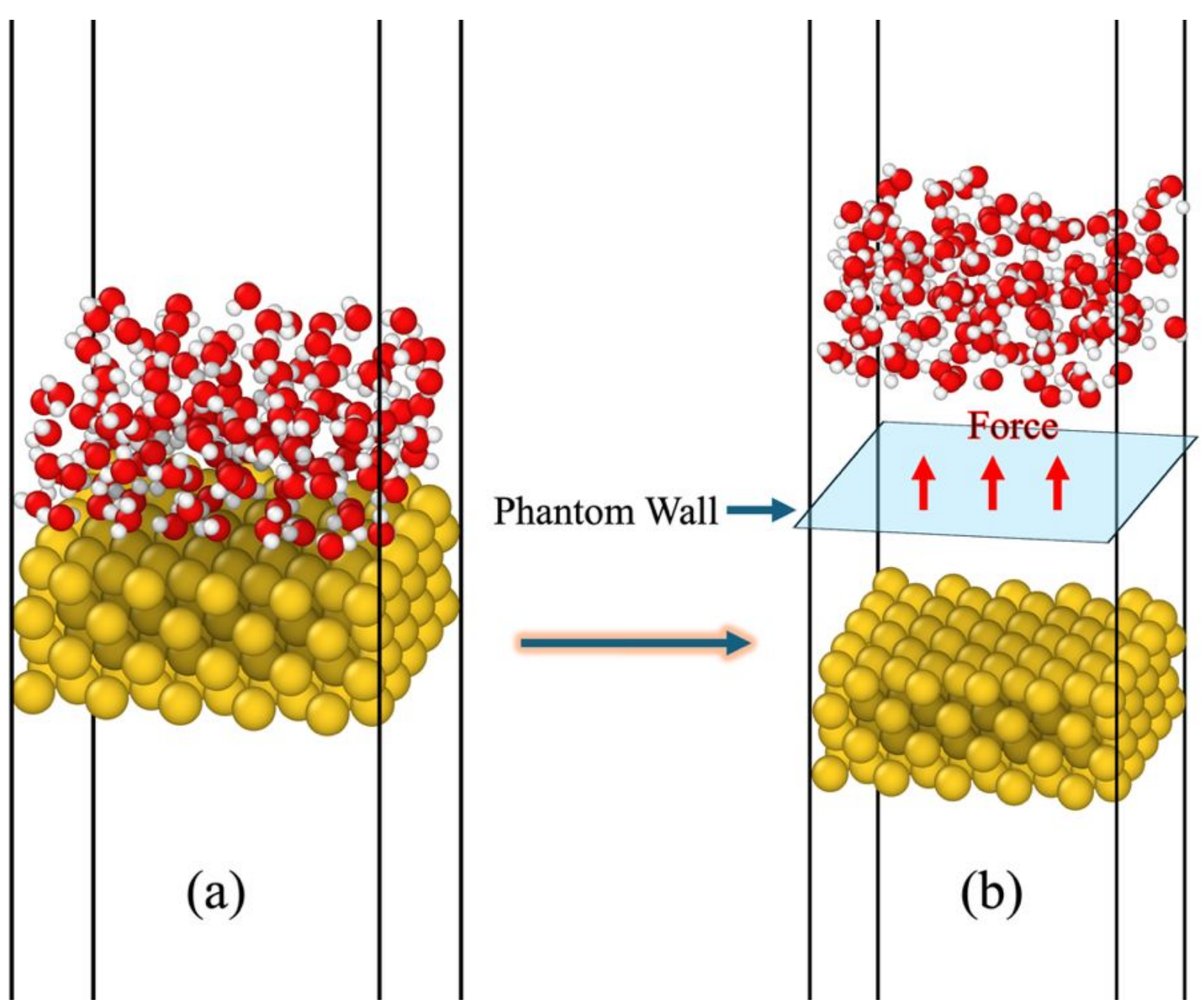

Figure S11. Phantom-wall work-of-adhesion calculation for the Au(001) surface. (a) Initial Au-water configuration and (b) the water column after the phantom wall has lifted it clear of the Au slab.

The clean-surface energies of the three facets were obtained separately by a slab energy-difference method. Both the slab and its periodic reference were built at the ReaxFF equilibrium lattice constant of a_0 = 4.16528 Å, obtained by relaxing a fully periodic bulk cell and confirmed by a residual pressure below 10^{-4} GPa. The reference energy per atom was verified to be independent of cell size across 108, 256, and 500 atoms. Each slab was constructed with the surface normal along z and lateral dimensions of at least 25 Å so that the cell exceeds twice the ReaxFF taper radius, and $E_{periodic}$ was evaluated for this fully periodic cell. Vacuum was then introduced along z without remapping the atomic coordinates; the atoms were relaxed by conjugate-gradient minimization at fixed cell dimensions, and E_{vacuum} was evaluated. The surface energy is:

$$\gamma = \frac{E_{vacuum} - E_{periodic}}{2A} \qquad \text{(S6)}$$

where A is the area of one exposed face, and the factor of two accounts for the two surfaces created. Convergence was verified in three ways. The surface energy varies by less than 0.01 mJ/m² across a threefold change in slab thickness on every facet, spanning 16.7 to 50.0 Å for Au(001), 17.7 to 53.0 Å for Au(110), and 21.6 to 72.1 Å for Au(111). It is converged with respect to vacuum spacing over the range 15 to 50 Å. The periodic reference reproduces the bulk energy per atom to within 1×10^{-4} kcal/mol per atom in every case. The resulting work-of-adhesion and surface-energy values for the three facets are reported in Tables 5 and 6 of the main text.

Section S9. X-ray diffraction characterization of the Au film

The Au-coated glass substrates were characterized by X-ray diffraction to verify the crystallographic texture of the film before the contact angle measurements, using the instrument and scan settings given in Section 4.2 of the main manuscript. Figure S12 shows the resulting diffractogram over the 30° to 60° range in 2θ. A single sharp reflection is present at 2θ = 38.2°, which corresponds to the (111) plane of FCC Au and is assigned following Parkhomenko et al. [25]. No other reflection rises above 0.5% of the (111) intensity anywhere in the scanned range, including the position where the Au(200) reflection would be expected near 44.4°. The film is therefore strongly <111>-textured, which supports the comparison drawn in Section 4 between the measured contact angle and the simulated Au(111) facet.

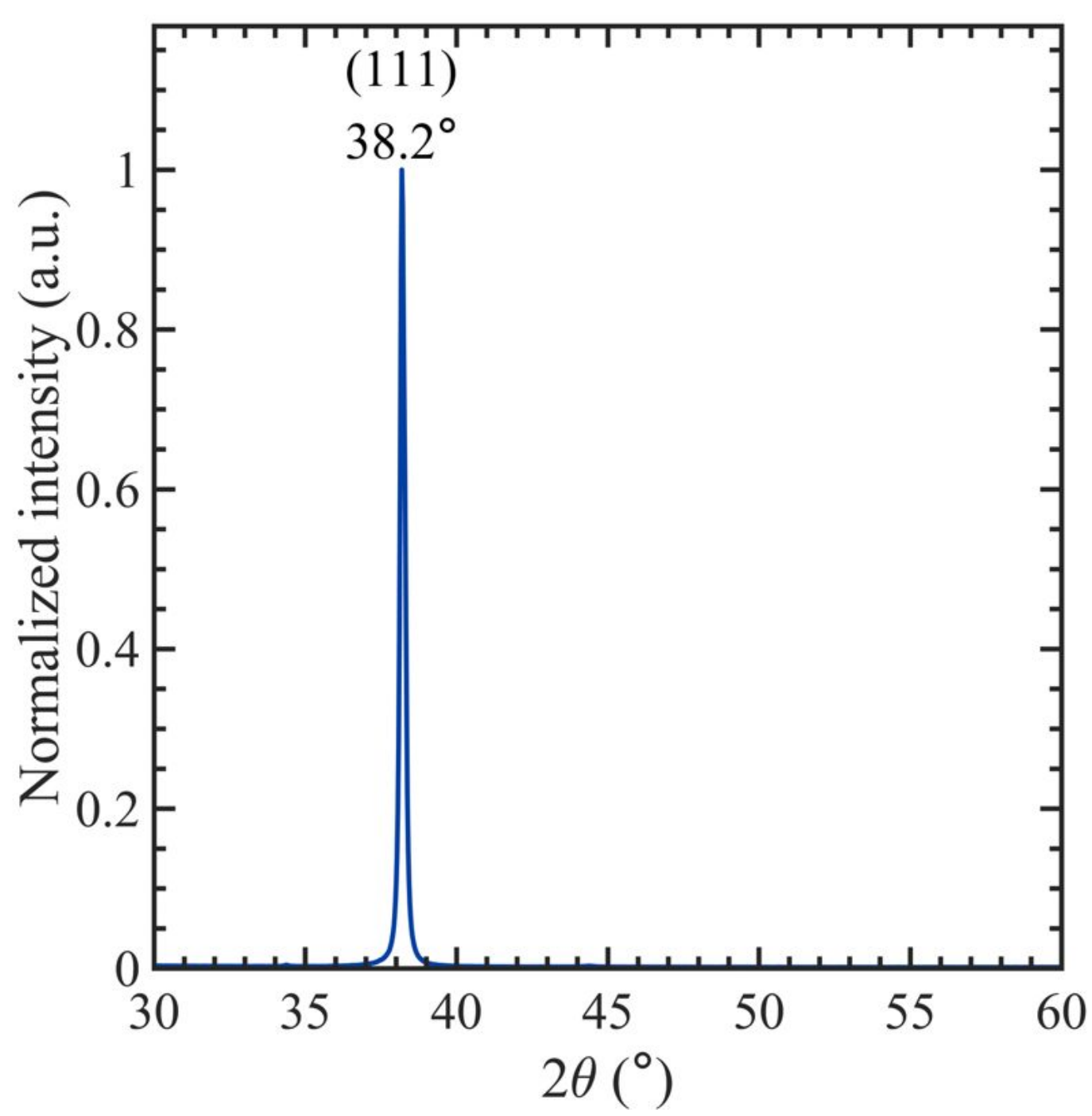

Figure S12. Diffractogram of the gold-coated glass samples. Assignment of the major peak at 38.2° as (111) is supported in the literature by Parkhomenko et. al.[25]

Section S10. ReaxFF Force Field Parameters

```
Reactive MD-force field: water/C/Ca/Ti ACKS2 July 18; Feb 4 2026
 39       ! Number of general parameters
  50.0000 !Overcoordination parameter
   9.5469 !Overcoordination parameter
   1.6725 !Valency angle conjugation parameter
   1.7224 !Triple bond stabilisation parameter
   6.8702 !Triple bond stabilisation parameter
  54.6742 !C2-correction
   1.0588 !Undercoordination parameter
   4.6000 !Triple bond stabilisation parameter
  12.1176 !Undercoordination parameter
  13.3056 !Undercoordination parameter
 -34.5448 !Triple bond stabilization energy
   0.0000 !Lower Taper-radius
  10.0000 !Upper Taper-radius
   2.8793 !Not used
  33.8667 !Valency undercoordination
   6.0891 !Valency angle/lone pair parameter
   1.0563 !Valency angle
   2.0384 !Valency angle parameter
   6.1431 !Not used
   6.9290 !Double bond/angle parameter
   0.3989 !Double bond/angle parameter: overcoord
   3.9954 !Double bond/angle parameter: overcoord
```

```
 -2.4837 !Not used
  8.5385 !Torsion/BO parameter
  6.7491 !Torsion overcoordination
  0.1414 !Torsion overcoordination
 -1.2327 !Conjugation 0 (not used)
  1.1348 !Conjugation
  1.5591 !vdWaals shielding
  0.1000 !Cutoff for bond order (*100)
  1.7602 !Valency angle conjugation parameter
  0.6991 !Overcoordination parameter
 50.0000 !Overcoordination parameter
  1.8512 !Valency/lone pair parameter
548.6451 !Softness
  0.0000 !Cutoff
  5.0000 !Molecular energy (not used)
  0.0000 !Molecular energy (not used)
  0.7903 !Valency angle conjugation parameter
14   ! Nr of atoms; cov.r; valency;a.m;Rvdw;Evdw;gammaEEM;cov.r2;#
         alfa;gammavdW;valency;Eunder;Eover;chiEEM;etaEEM;n.u.
         cov r3;Elp;Heat inc.;n.u.;n.u.;n.u.;n.u.
         ov/un;val1;n.u.;val3,vval4
C    1.3417   4.0000  12.0000   2.0464   0.1064   0.4670   1.1681   4.0000
     9.0000   1.5000   4.0000  40.0000  79.5548   5.3422   4.5000   0.0000
     1.1437   0.0000 181.0000   5.4236  19.2788  13.5366   3.1838   0.0000
    -4.0998   4.8750   1.0564   4.0000   2.9663   1.2500   0.4000  21.3612
H    0.9102   1.0000   1.0080   1.0996   0.1000   0.6683  -0.1000   1.0000
     9.2193   5.5154   1.0000   0.0000 121.1250   4.9673   6.2079   0.0000
```

```
-0.1000   0.0000  55.5000   1.1004   6.8959   0.0003   3.4114   0.0000
-6.5532   3.5000   1.0338   1.0000   2.8793   0.5000   0.1000   1.0000
O    1.2218   2.0000  15.9990   2.2033   0.2841   0.9750   1.0727   6.0000
8.8250 200.0000   4.0000  37.5000 116.0768   8.5000   7.9071   0.0000
0.9049   0.5488  68.0152   2.1943   2.3055   0.0021   5.4479   0.0000
-6.0011   3.6068   1.0493   4.0000   2.9225   0.5000   0.1000   1.0000
N    1.2333   3.0000  14.0000   2.2375   0.1447   1.0000   1.1748   5.0000
9.8626  12.6599   4.0000  30.3181 100.0000   6.0111   6.7037   0.0000
1.0433   0.7872 119.9837   0.7425   6.7920   2.7271   2.2882   0.0000
-2.0000   4.0000   1.0183   4.0000   2.8793   1.0000   0.1000   1.0000
S    1.9405   2.0000  32.0600   2.0677   0.2099   1.0336   1.5479   6.0000
9.9575   4.9055   4.0000  52.9998 112.1416   6.5000   8.2545   0.0000
1.4601   9.7177  71.1843   5.7487  23.2859  12.7147   2.2882   0.0000
-11.0000   2.7466   1.0338   6.2998   2.8793   1.0000   0.1000  10.0000
Mg   1.8315   2.0000  24.3050   2.2464   0.1806   0.5020   1.0000   2.0000
10.9186  27.1205   3.0000  38.0000   0.0000   0.9499   5.6130   0.0000
-1.3000   0.0000 127.9160  49.9248   0.3370   0.0000   2.2882   0.0000
-1.0823   2.3663   1.0564   6.0000   2.9663   1.0000   0.1000  10.0000
P    1.5994   3.0000  30.9738   1.7000   0.1743   1.0000   1.3000   5.0000
9.1909  14.9482   5.0000   0.0000   0.0000   1.6676   7.0946   0.0000
-1.0000  25.0000 125.6300   0.2187  21.4305  15.1425   2.2882   0.0000
-3.9294   3.4831   1.0338   5.0000   2.8793   1.0000   0.1000  10.0000
Na   1.8000   1.0000  22.9898   2.8270   0.1872   0.4000  -1.0000   1.0000
10.0000   2.5000   1.0000   0.0000   0.0000  -0.9871   6.7728   0.0000
-1.0000   0.0000  23.0445 100.0000   1.0000   0.0000   2.2882   0.0000
-2.5000   3.9900   1.0338   8.0000   2.5791   1.0000   0.1000  10.0000
Ti   2.0254   4.0000  47.8800   2.2105   0.1574   0.6311   0.1000   4.0000
```

```
   12.7041  16.6482   4.0000   0.1000   0.0000  -1.3647   6.8406   0.0000
   -1.0000   0.0000 143.1770  27.6505  -0.0753   0.0064   2.2882   0.0000
  -15.0000   3.8359   1.0338  12.0000   2.2632   1.0000   0.1000  10.0000
Cl  1.7140   1.0000  35.4500   1.9139   0.2000   0.3500  -1.0000   7.0000
   11.5345  10.1330   1.0000   0.0000   0.0000   9.9704   6.1703   0.0000
   -1.0000   1.2769 143.1770   6.2293   5.2294   0.1542   2.2882   0.0000
  -10.2080   2.9867   1.0338   6.2998   2.5791   1.0000   0.1000  10.0000
F   1.2100   1.0000  18.9984   1.8601   0.1200   0.3000  -0.1000   7.0000
   11.5000   7.5000   4.0000   9.2533   0.2000   9.0000  15.0000   0.0000
   -1.0000  35.0000   1.5000   6.9821   4.1799   1.0561   2.2882   0.0000
   -7.3000   2.6656   1.0493   4.0000   2.9225   1.0000   0.1000  10.0000
Ca  1.9058   2.0000  40.0870   2.3698   0.3719   0.6038  -1.0000   2.0000
    9.8681   5.0000   3.0000  38.0000   0.0000  -5.7471   7.3556   0.0000
   -1.3000   0.0000 220.0000  49.9248   0.3370   0.0000   3.7000   0.0000
   -3.9745   3.0069   1.0564   8.0000   2.9663   0.5000   0.1000  10.0000
Au  2.0074   1.0000 196.9665   2.1413   0.3730   0.9754  -1.0000   1.0000
   12.1061   2.1635   1.0000   0.0000   0.0000   3.7756   6.2893   0.0000
   -1.0000   0.0000  92.5070   6.2293   5.2294   0.1542   3.8074   0.0000
  -24.8303   2.9867   1.0338   6.2998   2.5791   0.5000   0.1000  10.0000
X  -0.1000   2.0000   1.0080   2.0000   0.0000   1.0000  -0.1000   6.0000
   10.0000   2.5000   4.0000   0.0000   0.0000  -0.1000  25.0000   0.0000
   -0.1000   0.0000  -2.3700   8.7410  13.3640   0.6690   0.1000   0.0000
  -11.0000   2.7466   1.0338   4.0000   2.8793   1.0000   0.1000  10.0000
66    ! Nr of bonds; Edis1;LPpen;n.u.;pbe1;pbo5;13corr;pbo6
                         pbe2;pbo3;pbo4;Etrip;pbo1;pbo2;ovcorr
 1  1  78.4266 115.3834  68.1631   0.5777  -0.2901   1.0000  34.9989   0.5101
         3.8560  -0.1640   8.2326   1.0000  -0.0585   6.7997   1.0000   0.0000
```

1 2 191.4398 0.0000 0.0000 -0.6539 0.0000 1.0000 6.0000 0.4898
6.3962 1.0000 0.0000 1.0000 -0.0607 6.9960 0.0000 0.0000
2 2 161.2898 0.0000 0.0000 -0.2387 0.0000 1.0000 6.0000 0.6279
13.2089 1.0000 0.0000 1.0000 -0.1674 6.9118 0.0000 0.0000
1 3 170.8787 152.2997 125.6008 0.2914 -0.2305 1.0000 16.7601 0.8645
3.9615 -0.5703 7.4065 1.0000 -0.2514 4.5085 0.0000 0.0000
3 3 222.6268 93.7249 50.8293 0.5588 -0.1000 1.0000 29.7503 0.0125
0.5865 -0.2030 9.1777 1.0000 -0.1357 6.4747 1.0000 0.0000
1 4 163.8300 145.4458 89.6879 -1.3368 -0.3468 1.0000 27.5160 0.1575
0.1817 -0.3114 7.1789 1.0000 -0.2345 4.5111 1.0000 0.0000
3 4 130.8596 169.4551 40.0000 0.3837 -0.1639 1.0000 35.0000 0.2000
1.0000 -0.3579 7.0004 1.0000 -0.1193 6.8773 1.0000 0.0000
4 4 157.9384 82.5526 152.5336 0.4010 -0.1034 1.0000 12.4261 0.5828
0.1578 -0.1509 11.9186 1.0000 -0.0861 5.4271 1.0000 0.0000
2 3 143.3409 0.0000 0.0000 -0.4886 0.0000 1.0000 6.0000 0.3927
1.3588 1.0000 0.0000 0.0000 -0.0781 4.0330 0.0000 0.0000
2 4 210.1187 0.0000 0.0000 -0.3705 0.0000 1.0000 6.0000 0.3284
5.8196 1.0000 0.0000 1.0000 -0.1104 5.5184 0.0000 0.0000
1 5 128.9942 74.5848 55.2528 0.1035 -0.5211 1.0000 18.9617 0.6000
0.2949 -0.2398 8.1175 1.0000 -0.1029 5.6731 1.0000 0.0000
2 5 151.5159 0.0000 0.0000 -0.4721 0.0000 1.0000 6.0000 0.6000
9.4366 1.0000 0.0000 1.0000 -0.0290 7.0050 1.0000 0.0000
3 5 0.0000 0.0000 0.0000 0.5563 -0.4038 1.0000 49.5611 0.6000
0.4259 -0.4577 12.7569 1.0000 -0.1100 7.1145 1.0000 0.0000
4 5 0.0000 0.0000 0.0000 0.4438 -0.2034 1.0000 40.3399 0.6000
0.3296 -0.3153 9.1227 1.0000 -0.1805 5.6864 1.0000 0.0000
5 5 96.1871 93.7006 68.6860 0.0955 -0.4781 1.0000 17.8574 0.6000

0.2723 -0.2373 9.7875 1.0000 -0.0950 6.4757 1.0000 0.0000
2 6 58.6896 0.0000 0.0000 -0.0203 -0.1418 1.0000 13.1260 0.0230
8.2136 -0.1310 0.0000 1.0000 -0.2692 6.4254 0.0000 24.4461
3 6 87.0227 0.0000 43.3991 0.0030 -0.3000 1.0000 36.0000 0.0250
0.0087 -0.2500 12.0000 1.0000 -0.0439 6.6073 1.0000 24.4461
6 6 32.3808 0.0000 0.0000 -0.0076 -0.2000 0.0000 16.0000 0.2641
4.8726 -0.2000 10.0000 1.0000 -0.0729 4.6319 0.0000 0.0000
1 7 110.0000 92.0000 0.0000 0.2171 -0.1418 1.0000 13.1260 0.6000
0.3601 -0.1310 10.7257 1.0000 -0.0869 5.3302 1.0000 0.0000
2 7 0.1466 0.0000 0.0000 0.2250 -0.1418 1.0000 13.1260 0.6000
0.3912 -0.1310 0.0000 1.0000 -0.1029 9.3302 0.0000 0.0000
3 7 201.0058 194.1410 0.0000 1.0000 -0.5000 1.0000 25.0000 0.4873
0.4358 -0.1571 15.8745 1.0000 -0.2431 6.3823 1.0000 0.0000
4 7 130.0000 0.0000 0.0000 0.2171 -0.1418 1.0000 13.1260 0.6000
0.3601 -0.1310 10.7257 1.0000 -0.0869 5.3302 1.0000 0.0000
6 7 0.1000 0.0000 0.0000 0.2500 -0.5000 1.0000 35.0000 0.6000
0.5000 -0.5000 20.0000 1.0000 -0.2000 10.0000 1.0000 0.0000
7 7 0.0000 0.0000 0.0000 0.2171 -0.5000 1.0000 35.0000 0.6000
0.5000 -0.5000 20.0000 1.0000 -0.2000 10.0000 1.0000 0.0000
1 8 0.0000 0.0000 0.0000 -1.0000 -0.3000 1.0000 36.0000 0.7000
10.1151 -0.3500 25.0000 1.0000 -0.1053 8.2003 1.0000 0.0000
2 8 0.0000 0.0000 0.0000 -1.0000 -0.3000 1.0000 36.0000 0.7000
10.1151 -0.3500 25.0000 1.0000 -0.1053 8.2003 1.0000 0.0000
3 8 45.8933 0.0000 0.0000 -0.1511 -0.3000 1.0000 36.0000 0.3105
5.8448 -0.3500 25.0000 1.0000 -0.0659 7.9140 1.0000 0.0000
4 8 0.0000 0.0000 0.0000 -1.0000 -0.3000 1.0000 36.0000 0.7000
10.1151 -0.3500 25.0000 1.0000 -0.1053 8.2003 1.0000 0.0000

5 8 0.0000 0.0000 0.0000 -1.0000 -0.3000 1.0000 36.0000 0.7000
10.1151 -0.3500 25.0000 1.0000 -0.1053 8.2003 1.0000 0.0000
6 8 0.0000 0.0000 0.0000 0.2500 -0.5000 1.0000 35.0000 0.6000
0.5000 -0.5000 20.0000 1.0000 -0.2000 10.0000 1.0000 0.0000
7 8 0.0000 0.0000 0.0000 0.2500 -0.5000 1.0000 35.0000 0.6000
0.5000 -0.5000 20.0000 1.0000 -0.2000 10.0000 1.0000 0.0000
8 8 64.4508 0.0000 0.0000 -0.3738 0.3000 0.0000 25.0000 0.2158
0.9915 -0.4000 12.0000 1.0000 -0.0515 5.0000 0.0000 0.0000
4 6 50.0000 10.0901 0.0000 -1.0000 -0.3000 1.0000 36.0000 0.7058
0.8567 -0.3487 17.4990 1.0000 -0.0794 8.2232 1.0000 0.0000
1 9 0.0000 0.0000 0.0000 -0.2872 -0.3000 1.0000 36.0000 0.0082
1.7973 -0.2500 20.0000 1.0000 -0.2578 6.5219 1.0000 0.0000
2 9 0.0000 0.0000 0.0000 -0.2872 -0.3000 1.0000 36.0000 0.0082
1.7973 -0.2500 20.0000 1.0000 -0.2578 6.5219 1.0000 0.0000
3 9 130.5629 37.6984 0.0000 0.9228 -0.3000 0.0000 36.0000 0.0850
0.1150 -0.2818 16.1571 1.0000 -0.1343 6.8264 0.0000 0.0000
4 9 130.5629 37.6984 0.0000 0.9228 -0.3000 0.0000 36.0000 0.0850
0.1150 -0.2818 16.1571 1.0000 -0.1343 6.8264 0.0000 0.0000
5 9 0.0000 0.0000 0.0000 -0.2872 -0.3000 1.0000 36.0000 0.0082
1.7973 -0.2500 20.0000 1.0000 -0.2578 6.5219 1.0000 0.0000
6 9 0.0000 0.0000 0.0000 -0.2872 -0.3000 1.0000 36.0000 0.0082
1.7973 -0.2500 20.0000 1.0000 -0.2578 6.5219 1.0000 0.0000
7 9 0.0000 0.0000 0.0000 -0.2872 -0.3000 1.0000 36.0000 0.0082
1.7973 -0.2500 20.0000 1.0000 -0.2578 6.5219 1.0000 0.0000
8 9 0.1000 0.0000 0.0000 0.2500 -0.5000 1.0000 35.0000 0.6000
0.5000 -0.5000 20.0000 1.0000 -0.2000 10.0000 1.0000 0.0000
9 9 80.1930 0.0000 0.0000 -0.8469 -0.2000 0.0000 16.0000 0.2022

0.7528 -0.1924 14.9725 1.0000 -0.0885 5.0000 0.0000 0.0000
1 10 0.0000 0.0000 0.0000 0.5000 -0.2000 0.0000 16.0000 0.5000
1.0001 -0.2000 15.0000 1.0000 -0.1000 10.0000 0.0000 0.0000
2 10 98.9788 0.0000 0.0000 -0.0572 -0.2000 0.0000 16.0000 1.1523
2.2822 -0.2000 15.0000 1.0000 -0.1093 5.1686 0.0000 0.0000
3 10 0.0000 0.0000 0.0000 0.5000 -0.2000 0.0000 16.0000 0.5000
1.0001 -0.2000 15.0000 1.0000 -0.1000 10.0000 0.0000 0.0000
4 10 0.0000 0.0000 0.0000 0.5000 -0.2000 0.0000 16.0000 0.5000
1.0001 -0.2000 15.0000 1.0000 -0.1000 10.0000 0.0000 0.0000
5 10 0.0000 0.0000 0.0000 0.5000 -0.2000 0.0000 16.0000 0.5000
1.0001 -0.2000 15.0000 1.0000 -0.1000 10.0000 0.0000 0.0000
6 10 0.0000 0.0000 0.0000 0.5000 -0.2000 0.0000 16.0000 0.5000
1.0001 -0.2000 15.0000 1.0000 -0.1000 10.0000 0.0000 0.0000
7 10 0.0000 0.0000 0.0000 0.5000 -0.2000 0.0000 16.0000 0.5000
1.0001 -0.2000 15.0000 1.0000 -0.1000 10.0000 0.0000 0.0000
8 10 0.0000 0.0000 0.0000 0.5000 -0.2000 0.0000 16.0000 0.5000
1.0001 -0.2000 15.0000 1.0000 -0.1000 10.0000 0.0000 0.0000
9 10 0.0000 0.0000 0.0000 0.5000 -0.2000 0.0000 16.0000 0.5000
1.0001 -0.2000 15.0000 1.0000 -0.1000 10.0000 0.0000 0.0000
10 10 0.2500 0.0000 0.0000 0.1803 -0.2000 0.0000 16.0000 0.3356
0.9228 -0.2000 15.0000 1.0000 -0.1178 5.6715 0.0000 0.0000
1 11 237.8781 0.0000 0.0000 -0.7438 -0.5000 1.0000 35.0000 1.0460
3.6661 -0.2500 15.0000 1.0000 -0.0800 5.4719 1.0000 0.0000
2 11 0.0000 0.0000 0.0000 -0.4643 0.0000 1.0000 6.0000 0.6151
12.3710 1.0000 0.0000 1.0000 -0.1008 8.5980 0.0000 0.0000
3 11 0.0000 0.0000 0.0000 -0.4643 0.0000 1.0000 6.0000 0.6151
12.3710 1.0000 0.0000 1.0000 -0.1008 8.5980 0.0000 0.0000

```
 4 11   0.0000   0.0000   0.0000  -0.4643   0.0000   1.0000   6.0000   0.6151
        12.3710   1.0000   0.0000   1.0000  -0.1008   8.5980   0.0000   0.0000
 5 11   0.0000   0.0000   0.0000  -0.4643   0.0000   1.0000   6.0000   0.6151
        12.3710   1.0000   0.0000   1.0000  -0.1008   8.5980   0.0000   0.0000
11 11 250.0765   0.0000   0.0000   0.2298  -0.3500   1.0000  25.0000   0.8427
         0.1167  -0.2500  15.0000   1.0000  -0.1506   7.3516   1.0000   0.0000
 1 12   0.0000   0.0000   0.0000  -0.0203  -0.1418   1.0000  13.1260   0.0230
         8.2136  -0.2500  20.0000   1.0000  -0.2692   6.4254   0.0000  24.4461
 2 12   0.0000   0.0000   0.0000  -0.0203  -0.1418   1.0000  13.1260   0.0230
         8.2136  -0.2500  20.0000   1.0000  -0.2692   6.4254   0.0000  24.4461
 3 12  49.4055   0.0000   0.0000   0.9603  -0.3000   0.0000  36.0000   0.0025
         0.4232  -0.2500  12.0000   1.0000  -0.1619   9.6512   0.0000  24.4461
12 12  22.8272   0.0000   0.0000   0.6166  -0.2000   0.0000  16.0000   0.8225
         1.0000  -0.2000  10.0000   1.0000  -0.0831   4.2291   0.0000   0.0000
 1 13  85.5942   0.0000   0.0000   0.3650  -0.2000   0.0000  16.0000   0.1488
         0.1726  -0.2000  15.0000   1.0000  -0.1380   5.9607   0.0000   0.0000
 2 13 126.5955   0.0000   0.0000   0.2800  -0.2000   0.0000  16.0000   0.3725
         0.0651  -0.2000  15.0000   1.0000  -0.1479   7.1445   0.0000   0.0000
 3 13 102.2246   0.0000   0.0000  -0.0394  -0.2000   1.0000  16.0000  -0.0145
         0.1377  -0.2000  15.0000   1.0000  -0.2213   5.5636   1.0000   0.0000
13 13 142.6814   0.0000   0.0000  -0.0100  -0.2000   0.0000  16.0000   0.3663
         0.2903  -0.2000  15.0000   1.0000  -0.1517   5.2066   0.0000   0.0000
29    ! Nr of off-diagonal terms; Ediss;Ro;gamma;rsigma;rpi;rpi2
 1  2   0.1240   1.6326   9.8721   1.1578  -1.0000  -1.0000
 2  3   0.0295   1.3181  10.1225   0.9069  -1.0000  -1.0000
 2  4   0.1294   1.3025   9.8751   1.0415  -1.0000  -1.0000
 1  3   0.2287   2.0452   9.0166   1.4046   1.1866   1.0375
```

```
 1  4   0.2000   1.8828   9.7673   1.3387   1.2578   1.1539
 3  4   0.1001   2.3274   9.0974   1.5236   1.0493   1.2531
 1  5   0.1408   1.8161   9.9393   1.7986   1.3021   1.4031
 2  5   0.0895   1.6239  10.0104   1.4640  -1.0000  -1.0000
 3  5   0.1022   1.9887  10.0605   1.5799   1.4000  -1.0000
 4  5   0.1505   1.9000  10.5104   1.8000   1.4000  -1.0000
 2  6   0.0100   1.6000  13.2979   1.8670  -1.0000  -1.0000
 3  6   0.0809   1.7000  11.4606   1.5177  -1.0000  -1.0000
 3  7   0.0534   1.7520  10.4281   1.8000   1.4498  -1.0000
 6  7   0.1801   1.8566   9.8498   0.1000  -1.0000  -1.0000
 3  8   0.0825   1.5904  11.3396   1.5905  -1.0000  -1.0000
 2  9   0.1750   1.7939  13.5000   0.0100  -1.0000  -1.0000
 3  9   0.1200   1.8000  10.5000   1.6526   1.4718  -1.0000
 1  9   0.2950   2.2000  11.0937   0.0100  -1.0000  -1.0000
 2 10   0.0376   1.6671   9.6285   1.2123  -1.0000  -1.0000
 3 10   0.1945   2.2766  11.2353  -1.0000  -1.0000  -1.0000
 1 11   0.1071   1.6243  11.0402   1.3176  -1.0000  -1.0000
 2 11   0.0431   1.7204  10.3632   0.5386  -1.0000  -1.0000
 4  9   0.1200   1.8000  10.5000   1.6526   1.4718  -1.0000
 1 12   0.2000   1.5000  14.0000   0.0010   0.0010  -1.0000
 2 12   0.0100   1.0610   9.7343   0.0010   0.0010  -1.0000
 3 12   0.1515   1.8913  12.5160   2.0022  -1.0000  -1.0000
 1 13   0.1910   2.0244   9.6929   1.8040  -1.0000  -1.0000
 2 13   0.1815   1.8645   9.0000   1.4797  -1.0000  -1.0000
 3 13   0.2364   2.0404  10.1555   1.8879  -1.0000  -1.0000
112    ! Nr of angles;at1;at2;at3;Thetao,o;ka;kb;pv1;pv2
 1  1  1  74.4118  38.0306   0.9605   0.0000   0.0100  36.6918   2.3203
```

636	1	1	2	67.2765	20.1739	3.3306	0.0000	0.0100	0.0000	1.1630
637	2	1	2	74.7224	38.7524	2.1423	0.0000	0.8474	0.0000	1.3144
638	1	2	2	0.0000	0.0000	6.0000	0.0000	0.0000	0.0000	1.0400
639	1	2	1	0.0000	3.4110	7.7350	0.0000	0.0000	0.0000	1.0400
640	2	2	2	0.0000	27.9213	5.8635	0.0000	0.0000	0.0000	1.0400
641	1	1	3	61.1001	38.5283	0.4917	0.0000	4.2373	0.0000	2.1649
642	3	1	3	72.0708	27.2877	2.3068	-10.4517	0.1000	0.0000	1.6107
643	1	1	4	64.7353	38.2645	1.1478	0.0000	1.1834	0.0000	2.8465
644	3	1	4	81.0672	41.9015	0.4878	0.0000	1.1019	0.0000	1.0000
645	4	1	4	89.7621	43.0000	0.5895	0.0000	1.1155	0.0000	1.0000
646	2	1	3	61.3200	29.1602	0.9036	0.0000	1.8063	0.0000	1.4867
647	2	1	4	68.7361	36.7162	1.6697	0.0000	0.2000	0.0000	3.0000
648	1	2	4	0.0000	0.0019	6.3000	0.0000	0.0000	0.0000	1.0400
649	1	3	1	74.2533	41.5372	0.4237	0.0000	2.3660	0.0000	1.0319
650	1	3	3	82.8809	21.0869	3.0902	0.0000	4.5310	20.1072	1.0105
651	1	3	4	70.3730	45.0000	1.4731	0.0000	2.9000	0.0000	2.4464
652	3	3	3	77.3022	33.4558	1.4033	0.0000	3.9048	0.0000	1.0000
653	3	3	4	77.0669	27.6795	1.6466	0.0000	2.9000	0.0000	1.5085
654	4	3	4	68.8583	40.4712	1.8369	0.0000	3.0072	0.0000	1.5773
655	1	3	2	90.0000	34.3702	0.4674	0.0000	0.6101	0.0000	1.0013
656	2	3	3	90.0000	26.3185	8.0000	0.0000	0.8615	0.0000	1.0579
657	2	3	4	68.3253	36.1953	7.5000	0.0000	0.1000	0.0000	1.0000
658	2	3	2	80.7909	19.0967	1.0887	0.0000	2.0594	0.0000	1.7720
659	1	4	1	71.2077	14.1180	3.3944	0.0000	2.8702	0.0000	1.2651
660	1	4	3	76.1064	23.7583	1.6308	0.0000	2.8701	0.0000	1.6732
661	1	4	4	71.3624	12.8120	3.1458	0.0000	2.8701	0.0000	1.1896
662	3	4	3	74.2922	23.0742	2.6248	-18.0069	3.0701	0.0000	1.6278

663	3	4	4	74.0840	31.1381	1.5175	-0.9193	3.0117	0.0000	1.3541
664	4	4	4	76.0945	32.1176	1.7767	0.0000	2.9983	0.0000	1.9677
665	1	4	2	69.1892	14.8553	2.7174	0.0000	0.2025	0.0000	1.3071
666	2	4	3	74.5555	45.0000	1.1948	0.0000	0.3956	0.0000	3.0000
667	2	4	4	78.8758	45.0000	0.5964	0.0000	0.5437	0.0000	1.0000
668	2	4	2	81.5738	7.0792	7.5000	0.0000	0.1000	0.0000	1.0000
669	1	2	3	0.0000	21.4989	1.0000	0.0000	0.1000	0.0000	1.1358
670	1	2	4	0.0000	0.0100	2.4974	0.0000	0.0000	0.0000	1.3777
671	1	2	5	0.0000	15.0000	3.0000	0.0000	0.0000	0.0000	1.0400
672	3	2	3	0.0000	15.0000	0.5640	0.0000	0.0000	0.0000	1.0400
673	3	2	4	0.0000	1.0235	0.1000	0.0000	0.0000	0.0000	3.0000
674	4	2	4	0.0000	0.0100	1.3170	0.0000	0.0000	0.0000	2.1165
675	2	2	3	0.0000	2.0000	0.0839	0.0000	0.0000	0.0000	2.9374
676	2	2	4	0.0000	0.0019	6.0000	0.0000	0.0000	0.0000	1.0400
677	1	1	5	74.4180	33.4273	1.7018	0.1463	0.5000	0.0000	1.6178
678	1	5	1	79.7037	28.2036	1.7073	0.1463	0.5000	0.0000	1.6453
679	2	1	5	63.3289	29.4225	2.1326	0.0000	0.5000	0.0000	3.0000
680	1	5	2	85.9449	38.3109	1.2492	0.0000	0.5000	0.0000	1.1000
681	1	5	5	85.6645	40.0000	2.9274	0.1463	0.5000	0.0000	1.3830
682	2	5	2	83.8555	5.1317	0.4377	0.0000	0.5000	0.0000	3.0000
683	2	5	5	97.0064	32.1121	2.0242	0.0000	0.5000	0.0000	2.8568
684	3	5	3	81.0926	30.2268	6.4132	-5.4471	2.5968	0.0000	3.0000
685	1	5	3	70.0000	35.0000	3.4223	0.0000	1.3550	0.0000	1.2002
686	1	3	5	57.3353	41.0012	1.0609	0.0000	1.3000	0.0000	3.0000
687	3	3	5	83.9753	31.0715	3.5590	0.0000	0.8161	0.0000	1.1776
688	2	3	5	89.8843	17.5000	3.3660	0.0000	2.0000	0.0000	2.0734
689	2	6	2	0.0000	49.8261	0.2093	0.0000	2.0870	0.0000	2.2895

690	2	2	6	0.0000	40.0366	3.1505	0.0000	1.1296	0.0000	1.1110
691	6	2	6	0.0000	0.5047	0.8000	0.0000	0.8933	0.0000	4.6650
692	2	6	6	0.0000	8.7037	0.0827	0.0000	3.5597	0.0000	1.1198
693	3	6	3	0.0000	9.2317	0.1000	0.0000	1.0000	0.0000	1.0920
694	6	3	6	0.0008	25.0000	8.0000	0.0000	1.0000	0.0000	3.0000
695	2	3	6	66.0423	5.0000	1.0000	0.0000	1.0000	0.0000	1.2500
696	2	6	3	0.0000	0.5000	0.1000	0.0000	1.0000	0.0000	3.0000
697	3	3	6	70.0000	20.0000	1.0000	0.0000	1.0000	0.0000	1.2500
698	3	7	3	90.0000	18.4167	0.6799	-8.0000	0.1310	0.0000	2.2321
699	2	3	7	72.6004	9.6150	0.8905	0.0000	3.5473	0.0000	1.0400
700	3	3	7	60.0000	40.0000	4.0000	0.0000	1.0000	0.0000	1.0400
701	3	2	7	0.0000	10.0000	1.0000	0.0000	1.0000	0.0000	1.0400
702	6	3	7	41.0995	3.2207	7.3523	0.0000	0.1101	0.0000	1.0947
703	7	3	7	62.1312	7.5931	0.1000	0.0000	0.5154	0.0000	2.1744
704	1	3	7	74.1394	8.5687	1.7132	0.0000	-0.6553	0.0000	2.2323
705	2	7	3	75.0000	25.0000	2.0000	0.0000	1.0000	0.0000	1.2500
706	3	7	7	70.0000	25.0000	2.0000	0.0000	1.0000	0.0000	1.2500
707	3	9	3	90.0000	30.4624	2.1468	0.0000	0.0500	0.0000	1.9485
708	9	3	9	90.0000	5.7486	5.0000	0.0000	2.0000	0.0000	1.1000
709	3	3	9	62.9344	15.0215	4.3743	0.0000	0.6168	0.0000	1.1673
710	3	9	9	33.7127	8.0623	3.4580	0.0000	0.0500	0.0000	2.6065
711	2	3	9	90.0000	9.7766	8.0000	0.0000	0.0505	0.0000	1.7257
712	1	3	9	90.0000	11.2108	1.4880	0.0000	0.5386	0.0000	2.1105
713	3	2	10	0.0000	0.0100	0.0100	0.0000	0.0000	0.0000	1.1456
714	11	1	11	77.8443	49.0744	5.9913	0.0000	0.7835	0.0000	2.3020
715	1	11	1	0.0000	19.9962	3.2299	0.0000	2.1012	0.0000	1.1537
716	1	11	11	0.0000	25.0000	1.0000	0.0000	1.0000	0.0000	1.0400

717	11	1	2	69.6421	10.0000	2.0000	0.0000	1.0000	0.0000	1.0400
718	4	9	4	90.0000	30.4624	2.1468	0.0000	0.0500	0.0000	1.9485
719	3	9	4	90.0000	30.4624	2.1468	0.0000	0.0500	0.0000	1.9485
720	9	4	9	90.0000	5.7486	5.0000	0.0000	2.0000	0.0000	1.1000
721	3	4	9	62.9344	15.0215	4.3743	0.0000	0.6168	0.0000	1.1673
722	4	3	9	62.9344	15.0215	4.3743	0.0000	0.6168	0.0000	1.1673
723	4	4	9	62.9344	15.0215	4.3743	0.0000	0.6168	0.0000	1.1673
724	4	4	9	62.9344	15.0215	4.3743	0.0000	0.6168	0.0000	1.1673
725	4	9	9	33.7127	8.0623	3.4580	0.0000	0.0500	0.0000	2.6065
726	2	4	9	90.0000	9.7766	8.0000	0.0000	0.0505	0.0000	1.7257
727	1	4	9	90.0000	11.2108	1.4880	0.0000	0.5386	0.0000	2.1105
728	3	12	3	90.0000	5.2360	6.0000	0.0000	1.9491	0.0000	1.0000
729	12	3	12	27.0723	4.9264	1.7778	0.0000	0.3851	0.0000	1.4855
730	2	3	12	1.0000	1.5989	6.0000	0.0000	0.6668	0.0000	1.0000
731	3	3	12	90.0000	10.0000	1.0000	0.0000	1.0000	0.0000	2.0000
732	1	3	12	70.0000	0.0000	1.0000	0.0000	1.0000	0.0000	2.0000
733	1	1	13	58.3918	13.9641	2.0300	0.0000	1.2404	0.0000	2.2787
734	2	1	13	76.5969	12.6012	2.4132	0.0000	0.8279	0.0000	2.5627
735	1	13	1	71.3861	3.9232	2.1478	0.0000	1.1259	0.0000	2.1341
736	1	13	2	63.1192	38.3129	2.6511	0.0000	1.0152	0.0000	1.0000
737	2	13	2	47.1487	9.2600	2.9275	0.0000	1.3836	0.0000	2.5657
738	1	13	13	20.0000	7.2189	1.6647	0.0000	0.9966	0.0000	1.2857
739	2	13	13	50.6566	3.9009	0.1116	0.0000	0.7627	0.0000	1.1339
740	2	3	13	70.0000	5.2164	4.7207	0.0000	0.3160	0.0000	1.3227
741	3	2	13	1.5321	6.6609	4.1035	0.0000	0.1100	0.0000	1.2249
742	2	13	3	30.0000	10.0000	2.0000	0.0000	1.0000	0.0000	1.2500
743	3	3	13	80.3915	36.3302	1.5083	0.0000	1.0000	0.0000	1.5506

```
 3 13  3  14.1302   7.6175   6.2730   0.0000   0.5000   0.0000   1.0917
13  3 13   0.1000  10.0000   1.0000   0.0000   0.5000   0.0000   1.0000
 3 13 13   0.0100  35.9203   0.5472   0.0000   0.0412   0.0000   1.7535
73    ! Nr of torsions;at1;at2;at3;at4;;V1;V2;V3;V2(BO);vconj;n.u;n
 1  1  1  1   0.0734  56.5103   0.0000  -5.5375  -2.6113   0.0000   0.0000
 1  1  1  2   0.2335  44.4534   0.5000  -5.5234  -3.0000   0.0000   0.0000
 2  1  1  2  -0.0100  59.3937   0.5000  -8.0000  -3.0000   0.0000   0.0000
 1  1  1  3  -0.1605   9.1977  -1.0000  -2.5000  -2.1235   0.0000   0.0000
 2  1  1  3   0.4631  41.1330   0.7425  -7.5125  -1.1040   0.0000   0.0000
 3  1  1  3  -1.0000  30.3068  -1.0000  -8.5000  -0.0100   0.0000   0.0000
 1  1  3  1   1.0000  45.3654  -0.7715  -3.2711  -0.0100   0.0000   0.0000
 1  1  3  2   1.0000 150.0000   1.0000  -6.6407  -0.0100   0.0000   0.0000
 2  1  3  1   1.0000 143.9644   0.6834  -6.1023  -1.1889   0.0000   0.0000
 2  1  3  2  -1.0000  74.4134   1.0000  -3.3595  -3.5000   0.0000   0.0000
 1  1  3  3   1.0000 133.2711   0.5065  -2.5561  -0.0100   0.0000   0.0000
 2  1  3  3   0.2238  53.9090   0.8685  -2.7064  -3.5000   0.0000   0.0000
 3  1  3  1  -1.0000  66.2620   0.7534  -3.3533  -2.2258   0.0000   0.0000
 3  1  3  2   0.6489  19.7090  -1.0000  -3.3466  -0.1540   0.0000   0.0000
 3  1  3  3  -1.0000  86.4981   1.0000  -3.5833  -0.0610   0.0000   0.0000
 1  3  3  1   1.0000   0.1000   1.0000  -6.9024  -0.0100   0.0000   0.0000
 1  3  3  2   0.9093  23.6198  -0.1431  -2.4750  -3.5000   0.0000   0.0000
 2  3  3  2  -1.6122   5.0000  -1.0000  -2.5394  -0.9921   0.0000   0.0000
 1  3  3  3   2.5000   2.1016   0.9647  -2.6000  -0.9972   0.0000   0.0000
 2  3  3  3  -2.5000  75.7606  -0.6120  -7.8633  -1.2407   0.0000   0.0000
 3  3  3  3  -0.5000   5.0000   1.0000  -2.5000  -0.9000   0.0000   0.0000
 1  1  4  2   0.2700  43.1323  -0.4952  -7.6538  -1.9825   0.0000   0.0000
 2  1  4  2  -0.5047  82.9784   0.8701  -7.6680  -2.1051   0.0000   0.0000
```

771	3	1	4	2	0.8306	15.5307	1.0000	-2.5000	-2.5261	0.0000	0.0000
772	3	1	1	4	-0.8051	19.8307	1.0000	-3.7979	-0.9511	0.0000	0.0000
773	4	1	1	4	1.0000	36.1913	1.0000	-3.4095	-1.7241	0.0000	0.0000
774	1	1	4	1	1.0000	32.6616	0.3481	-6.4524	-1.6589	0.0000	0.0000
775	3	1	4	1	-1.0000	-5.0000	1.0000	-2.5000	-1.8038	0.0000	0.0000
776	2	1	1	4	0.7529	50.8010	-0.5000	-4.3471	-1.9000	0.0000	0.0000
777	4	1	4	2	0.3787	13.7301	0.6579	-8.2500	-2.0202	0.0000	0.0000
778	2	1	4	1	-1.0000	76.7186	0.1194	-8.0000	-1.5996	0.0000	0.0000
779	0	1	2	0	0.0000	0.0000	0.0000	0.0000	0.0000	0.0000	0.0000
780	0	2	2	0	0.0000	0.0000	0.0000	0.0000	0.0000	0.0000	0.0000
781	0	2	3	0	0.0000	0.1000	0.0200	-2.5415	0.0000	0.0000	0.0000
782	0	1	1	0	0.0000	60.0000	0.3000	-4.0000	-2.0000	0.0000	0.0000
783	0	3	3	0	0.5511	25.4150	1.1330	-5.1903	-1.0000	0.0000	0.0000
784	0	1	4	0	0.2176	40.4126	0.3535	-3.9875	-2.0051	0.0000	0.0000
785	0	2	4	0	0.0000	0.1032	0.3000	-5.0965	0.0000	0.0000	0.0000
786	0	3	4	0	1.1397	61.3225	0.5139	-3.8507	-2.7831	0.0000	0.0000
787	0	4	4	0	0.7265	44.3155	1.0000	-4.4046	-2.0000	0.0000	0.0000
788	4	1	4	4	-0.0949	8.7582	0.3310	-7.9430	-2.0000	0.0000	0.0000
789	0	1	5	0	4.0885	78.7058	0.1174	-2.1639	0.0000	0.0000	0.0000
790	0	5	5	0	-0.0170	-56.0786	0.6132	-2.2092	0.0000	0.0000	0.0000
791	0	2	5	0	0.0000	0.0000	0.0000	0.0000	0.0000	0.0000	0.0000
792	2	3	5	3	2.5000	2.5000	0.2237	-10.0000	-1.0000	0.0000	0.0000
793	0	3	5	0	-2.5000	50.0000	-0.5000	-10.0000	-1.0000	0.0000	0.0000
794	0	6	6	0	0.0000	0.0000	0.1200	-2.4426	0.0000	0.0000	0.0000
795	0	2	6	0	0.0000	0.0000	0.1200	-2.4847	0.0000	0.0000	0.0000
796	0	3	6	0	0.0000	0.0000	0.1200	-2.4703	0.0000	0.0000	0.0000
797	1	1	1	7	-0.3232	14.3871	0.1823	-9.8682	-1.7255	0.0000	0.0000

```
 7  1  1  7  -0.1452  50.0000  -0.1915  -8.0773  -1.7255   0.0000   0.0000
 0  1  7  0   4.0000  45.8264   0.9000  -4.0000   0.0000   0.0000   0.0000
 0  7  7  0   4.0000  45.8264   0.9000  -4.0000   0.0000   0.0000   0.0000
 2  1  3  7  -1.5000  18.9285   0.3649  -6.1208   0.0000   0.0000   0.0000
 2  3  7  3   1.5000  -1.0000   0.2575  -6.2100   0.0000   0.0000   0.0000
 1  3  7  3  -1.4375  -0.8700   0.9861  -2.5424   0.0000   0.0000   0.0000
 7  3  7  3  -1.5000  21.5086  -1.0000  -4.8869   0.0000   0.0000   0.0000
 2  1  3  9   0.1714  69.9743   0.9170  -7.9557   0.0000   0.0000   0.0000
 1  1  3  9   0.2500  76.5218   1.0000  -2.5503   0.0000   0.0000   0.0000
 3  1  3  9  -0.2500  50.5929  -0.2500  -6.9285   0.0000   0.0000   0.0000
 2  3  9  3  -0.2500   0.0100  -0.5000  -4.6984   0.0000   0.0000   0.0000
 1  1  1 11   0.5000   0.1000   0.4683 -11.5274  -1.7255   0.0000   0.0000
 2  1  1 11   0.0000  49.3871   0.2000 -10.5765  -1.7255   0.0000   0.0000
11  1  1 11  -0.5000  95.4727  -0.2080  -4.8579  -1.7255   0.0000   0.0000
 0  1 11  0   4.0000  45.8264   0.9000  -4.0000   0.0000   0.0000   0.0000
 0 11 11  0   4.0000  45.8264   0.8897  -4.0000   0.0000   0.0000   0.0000
 2  1  4  9   0.1714  69.9743   0.9170  -7.9557   0.0000   0.0000   0.0000
 1  1  4  9   0.2500  76.5218   1.0000  -2.5503   0.0000   0.0000   0.0000
 3  1  4  9  -0.2500  50.5929  -0.2500  -6.9285   0.0000   0.0000   0.0000
 4  1  4  9  -0.2500  50.5929  -0.2500  -6.9285   0.0000   0.0000   0.0000
 4  1  3  9  -0.2500  50.5929  -0.2500  -6.9285   0.0000   0.0000   0.0000
 2  4  9  3  -0.2500   0.0100  -0.5000  -4.6984   0.0000   0.0000   0.0000
 2  4  9  4  -0.2500   0.0100  -0.5000  -4.6984   0.0000   0.0000   0.0000
 0    ! Nr of hydrogen bonds;at1;at2;at3;Rhb;Dehb;vhb1
```

References:

(1) Merabia, S.; Shenogin, S.; Joly, L.; Keblinski, P.; Barrat, J.-L.; Rice, S. A. Heat Transfer from Nanoparticles: A Corresponding State Analysis. *Proc. Natl. Acad. Sci. U. S. A.* **2009**, *106* (36), 15113–15118.

(2) Tay, K. A.; Bresme, F. Wetting Properties of Passivated Metal Nanocrystals at Liquid−Vapor Interfaces: A Computer Simulation Study. *J. Am. Chem. Soc.* **2006**, *128* (43), 14166–14175. https://doi.org/10.1021/ja061901w.

(3) Heinz, H.; Vaia, R. A.; Farmer, B. L.; Naik, R. R. Accurate Simulation of Surfaces and Interfaces of Face-Centered Cubic Metals Using 12−6 and 9−6 Lennard-Jones Potentials. *J. Phys. Chem. C* **2008**, *112* (44), 17281–17290. https://doi.org/10.1021/jp801931d.

(4) Berg, A.; Peter, C.; Johnston, K. Evaluation and Optimization of Interface Force Fields for Water on Gold Surfaces. *J. Chem. Theory Comput.* **2017**, *13* (11), 5610–5623. https://doi.org/10.1021/acs.jctc.7b00612.

(5) Gim, S.; Cho, K. J.; Lim, H.-K.; Kim, H. Structure, Dynamics, and Wettability of Water at Metal Interfaces. *Sci. Rep.* **2019**, *9* (1), 14805. https://doi.org/10.1038/s41598-019-51323-5.

(6) Dou, Y.; Zhigilei, L. V.; Winograd, N.; Garrison, B. J. Explosive Boiling of Water Films Adjacent to Heated Surfaces: A Microscopic Description. *J. Phys. Chem. A* **2001**, *105* (12), 2748–2755. https://doi.org/10.1021/jp003913o.

(7) Ren, P.; Ponder, J. W. Polarizable Atomic Multipole Water Model for Molecular Mechanics Simulation. *J. Phys. Chem. B* **2003**, *107* (24), 5933–5947. https://doi.org/10.1021/jp027815+.

(8) Lee, S. H. Temperature Dependence on Structure and Self-Diffusion of Water: A Molecular Dynamics Simulation Study Using SPC/E Model. *Bull. Korean Chem. Soc.* **2013**, *34* (12), 3800–3804.

(9) Khusnutdinoff, R. M.; Mokshin, A. V. Vibrational Features of Water at the Low-Density/High-Density Liquid Structural Transformations. *Phys. Stat. Mech. Its Appl.* **2012**, *391* (9), 2842–2847. https://doi.org/10.1016/j.physa.2011.12.037.

(10) Walrafen, G. E.; Chu, Y. C.; Piermarini, G. J. Low-Frequency Raman Scattering from Water at High Pressures and High Temperatures. *J. Phys. Chem.* **1996**, *100* (24), 10363–10372. https://doi.org/10.1021/jp960140o.

(11) Walrafen, G. E. Raman Spectral Studies of the Effects of Temperature on Water and Electrolyte Solutions. *J. Chem. Phys.* **1966**, *44* (4), 1546–1558. https://doi.org/10.1063/1.1726891.

(12) Skinner, L. B.; Huang, C.; Schlesinger, D.; Pettersson, L. G. M.; Nilsson, A.; Benmore, C. J. Benchmark Oxygen-Oxygen Pair-Distribution Function of Ambient Water from x-Ray Diffraction Measurements with a Wide Q-Range. *J. Chem. Phys.* **2013**, *138* (7), 074506. https://doi.org/10.1063/1.4790861.

(13) REFPROP. *NIST* **2013**.

(14) Pamato, M. G.; Wood, I. G.; Dobson, D. P.; Hunt, S. A.; Vočadlo, L. The Thermal Expansion of Gold: Point Defect Concentrations and Pre-Melting in a Face-Centred Cubic Metal. *J. Appl. Crystallogr.* **2018**, *51* (2), 470–480. https://doi.org/10.1107/S1600576718002248.

(15) Keith, J. A.; Fantauzzi, D.; Jacob, T.; van Duin, A. C. T. Reactive Forcefield for Simulating Gold Surfaces and Nanoparticles. *Phys. Rev. B* **2010**, *81* (23), 235404. https://doi.org/10.1103/PhysRevB.81.235404.

(16) Kittel, C. *Introduction to Solid State Physics*, 8th Edition.; 2004.
(17) Arblaster, J. W. Thermodynamic Properties of Gold. *J. Phase Equilibria Diffus.* **2016**, *37* (2), 229–245. https://doi.org/10.1007/s11669-016-0449-z.
(18) Ambrosetti, A.; Silvestrelli, P. L. Cohesive Properties of Noble Metals by van Der Waals--Corrected Density Functional Theory: Au, Ag, and Cu as Case Studies. *Phys. Rev. B* **2016**, *94* (4), 045124. https://doi.org/10.1103/PhysRevB.94.045124.
(19) Chan, B.; Yim, W.-L. Accurate Computation of Cohesive Energies for Small to Medium-Sized Gold Clusters. *J. Chem. Theory Comput.* **2013**, *9* (4), 1964–1970. https://doi.org/10.1021/ct400047y.
(20) Paniagua-Guerra, L. E.; Ramos-Alvarado, B. Thermal Transport across Flat and Curved Gold–Water Interfaces: Assessing the Effects of the Interfacial Modeling Parameters. *J. Chem. Phys.* **2023**, *158* (13), 134717. https://doi.org/10.1063/5.0140878.
(21) Ramos-Alvarado, B.; Kumar, S.; Peterson, G. P. Wettability of Graphitic-Carbon and Silicon Surfaces: MD Modeling and Theoretical Analysis. *J. Chem. Phys.* **2015**, *143* (4), 044703. https://doi.org/10.1063/1.4927083.
(22) de Ruijter, M. J.; Blake, T. D.; De Coninck, J. Dynamic Wetting Studied by Molecular Modeling Simulations of Droplet Spreading. *Langmuir* **1999**, *15* (22), 7836–7847. https://doi.org/10.1021/la990171l.
(23) Leroy, F.; dos Santos, D. J. V. A.; Müller-Plathe, F. Interfacial Excess Free Energies of Solid–Liquid Interfaces by Molecular Dynamics Simulation and Thermodynamic Integration. *Macromol. Rapid Commun.* **2009**, *30* (9–10), 864–870. https://doi.org/10.1002/marc.200800746.
(24) Thompson, A. P.; Aktulga, H. M.; Berger, R.; Bolintineanu, D. S.; Brown, W. M.; Crozier, P. S.; in 't Veld, P. J.; Kohlmeyer, A.; Moore, S. G.; Nguyen, T. D.; Shan, R.; Stevens, M. J.; Tranchida, J.; Trott, C.; Plimpton, S. J. LAMMPS - a Flexible Simulation Tool for Particle-Based Materials Modeling at the Atomic, Meso, and Continuum Scales. *Comput. Phys. Commun.* **2022**, *271*, 108171. https://doi.org/10.1016/j.cpc.2021.108171.
(25) Parkhomenko, R.; Alexeyev, A.; Morozova, N.; Igumenov, I. Syntheses, Structures, and Thermal Properties of New Au(III) Organometallic Compounds with Ancillary S-, O-, and/or N-Donor Ligands. *J. Coord. Chem.* **2012**, *65* (18), 3227–3237. https://doi.org/10.1080/00958972.2012.713476.
